# Differential localization of Hessian fly candidate effectors in resistant and susceptible wheat plants

**DOI:** 10.1002/pld3.246

**Published:** 2020-08-14

**Authors:** Zainab Aljbory, Michael J. Aikins, Yoonseong Park, Gerald R. Reeck, Ming‐Shun Chen

**Affiliations:** ^1^ Department of Entomology Kansas State University Manhattan KS USA; ^2^ College of Agriculture Green University of Al Qasim Iraq; ^3^ Department of Biochemistry and Molecular Biophysics Kansas State University Manhattan KS USA; ^4^ Hard Winter Wheat Genetics Research Unit USDA‐ARS Kansas State University Manhattan KS USA

**Keywords:** agroinfiltration, Hessian fly effectors, hypersensitive reaction, immunostaining, *Nicotiana benthamiana*, plant resistance

## Abstract

Hessian fly *Mayetiola destructor* is a notorious pest of wheat. Previous studies suggest that Hessian fly uses effector‐based mechanisms to attack wheat plants during parasitism, but no direct evidence has been reported to support this postulation. Here, we produced recombinant proteins for five Family‐1 candidate effectors and antibodies. Indirect immunostaining and western blots were carried out to examine the localization of Hessian fly Family‐1 proteins in plant and insect tissues. Confocal images revealed that Family‐1 putative effectors were exclusively produced in the basal region of larval salivary glands, which are directly linked to the mandibles’ ducts for effector injection. The five Family‐1 proteins were detected in infested host plants on western blots. Indirect immunostaining of sectioned host tissues around the feeding site revealed strikingly different localization patterns between resistant and susceptible plants. In susceptible plants, the Family‐1 proteins penetrated from the feeding cell into deep tissues, indicative of movement between cells during nutritive cell formation. In contrast, the Hessian fly proteins were primarily limited to the initially attacked cells in resistant plants. The limitation of effectors’ spread in resistant plants was likely due to wall strengthening and rapid hypersensitive cell death. Cell death was found in *Nicotiana benthamiana* in association with hypersensitive reaction triggered by the Family‐1 effector SSGP‐1A2. Our finding represents a significant progress in visualizing insect effectors in host tissues and mechanisms of plant resistance and susceptibility to gall midge pests.

## 1 INTRODUCTION

In plant–parasite interactions, biotrophic parasites deliver effector proteins into host tissues to modulate plant immunity and to reprogram host metabolic pathways (Jones and Dangl, 2006; Torto‐Alalibo et al., 2009; Stuart et al., 2012; Kaloshian and Walling, 2016; Toruño et al., 2016). Gram‐negative bacterial pathogens deliver effector proteins into the cytoplasm of host cells using the Type III secretion system (Park et al., 2017). Parasitic insects produce effector proteins in their salivary glands and inject them into host plants using insect mandibles (Stuart et al., 2012; Kaloshian and Walling, 2016). The purpose of injecting effectors into host plants by parasites is to manipulate plants for the benefit of the parasites, however, specific physiological functions of individual effectors vary widely. For example, the two effectors PthXo1 and AvrZa7 from the bacterial pathogen *Xanthomonas oryzae* induce the expression of genes encoding sugar transporters (Chen, Hou et al., 2010). The root‐knot nematode effector MiPFN3 inhibits actin polymerization (Leelarasamee, Zhang, & Gleason 2018). Protein C002 is required for pea aphid feeding, possibly by overcoming the action of a protein that detects cell wall or membrane damage (Mutti et al., 2008). The effector Mp1 from the aphid *Myzus persicae* reduces the amount of the host protein ‘Vacuolar Protein Sorting Associated protein 52’ (VPS52) (Rodriguez et al., 2017), whereas the effector Me10 from the potato aphid *Macrosiphum euphorbiae* suppresses host immunity by modulating the functions of the 14‐3‐3 protein TFT7 in tomato (Chaudhary et al., 2019). When a parasite effector is recognized by the plant surveillance system, it triggers an acute plant defense response, which is called effector‐triggered immunity (Jones and Dangl, 2006). The parasite effector is called an avirulence (Avr) factor, and the host protein that recognizes the avirulence factor is called resistance (R) protein. This specific recognition between a parasite Avr protein and a host R protein was originally described as a gene‐for‐gene interaction (Flor, 1956).

The gall midge *Mayetiola destructor* Say, also called Hessian fly, is a destructive, parasitic pest of wheat. A single Hessian fly larva can convert a whole wheat seedling into a “gall” by inducing nutritive cells at the feeding site, inhibiting wheat growth while keeping the plant alive (Byers and Gallun, 1972; Harris et al., 2010; Stuart et al., 2012). Unlike other insects with long stylets, the mandibles of Hessian fly larvae are very small, and can hardly punch through a single cell. This apparent physical limitation of small mouthparts does not prevent larvae from obtaining sustained nourishment during feeding. Without long stylets that can reach phloem to secure sufficient nutrient supplies, Hessian fly larvae inject effector proteins into host tissues to induce the formation of nutritive cells (Harris et al., 2006), which makes inaccessible nutrients accessible to Hessian fly larvae and allow cell contents to migrate through compromised cell wall into adjacent cells toward the insect mandibles due to the negative pressure implied through insect sucking (Harris et al., 2006; Grover, 1995; Williams et al., 2011; Khajuria et al., 2013). The salivary glands of a Hessian fly larva are structured for the production and secretion of candidate effectors, with the expanded base region connected directly to the ducts of the mandibles for effector injection (Stuart and Hatchett, 1987). Transcriptomic analyses reveal that salivary glands produce a very high proportion of transcripts encoding Secreted Salivary Gland Proteins (SSGPs) (Chen et al., 2008). Genome sequencing has identified numerous families of genes encoding nearly 2,000 putative effectors (Zhao et al., 2015).

Among these families, Family‐1 (also called SSGP‐1) contains genes with the most abundant transcripts. Over 30% of total transcripts in the salivary glands of first instars are derived from Family‐1 genes (Chen, Liu et al., 2010). Our recent studies investigating other Hessian fly‐related gall midges have revealed that Family‐1 members are also abundant in the barley midge *Mayetiola hordei* and the oat midge *Mayetiola avenae* (Al‐Jbory, El‐Bouhssini et al., 2018) but not in the wheat midge *Sitodiplosis mosellana* (Al‐Jbory, Anderson et al., 2018). There are seven genes in Family‐1, which are named as *SSGP‐1A1*, *SSGP‐1A2*, *SSGP‐1B1*, *SSGP‐1C1*, *SSGP‐1C2*, *SSGP‐1D1*, and *SSGP‐1E1* (Chen, Liu et al., 2010) The signal peptide is almost identical among all members with variation in the mature protein regions (Figure S1). Hessian fly interacts with wheat in a typical gene‐for‐gene relationship (Hatchett and Gallun, 1970). Major dominant wheat genes, *H1* to *H34*, mediate antibiosis resistance, resulting in the death of Hessian fly larvae within a few days (Li et al., 2013; Harris et al., 2010). Mobilization of membrane lipids, strengthening of cell walls, and production of reactive oxygen species appear crucial for wheat resistance during incompatible interactions with Hessian fly (Liu et al., 2007, Liu et al., 2010; Zhu et al., 2012; Khajuria et al., 2013; Bacete et al., 2018). Indications of necrotic lesions have been observed in resistant plants carrying certain R genes (Grover, 1995, Friebe et al., 1990; Hatchett et al., 1993). Signs of cell death at the larval attack site were reported from wheat genotypes carrying the R genes *H6*, *H9*, *H13*, and *H26* (Harris et al., 2010).

Even though numerous effector‐like genes have been identified from the insect (Chen et al., 2008; Zhao et al., 2015), no direct evidence has been reported for their injection into host tissues and their specific roles once they are injected into host tissues. The objective of this study is toward answering questions about effector proteins in the interactions between wheat and Hessian fly using Family‐1 effectors as examples. We produced recombinant proteins and corresponding antibodies for five Family‐1 members: *SSGP‐1A2*, *SSGP‐1B1*, *SSGP‐1C1*, *SSGP‐1D1*, and *SSGP‐1E1* (Figure S1). Immuno‐techniques, including western blots and indirect immunostaining, were used to determine when and which insect tissues produce Family‐1 effectors; whether Family‐1 proteins are injected into host tissues; and where they are located in resistant and susceptible host tissues once they are injected. We also tested whether effectors of Family‐1 can induce hypersensitivity and subsequent cell death via agroinfiltration‐mediated transient expression of SSGP‐1A2 as an example.

## 2 RESULTS

### 2.1 Family‐1 effectors are expressed in insects at different life stages

**FIGURE 1:**
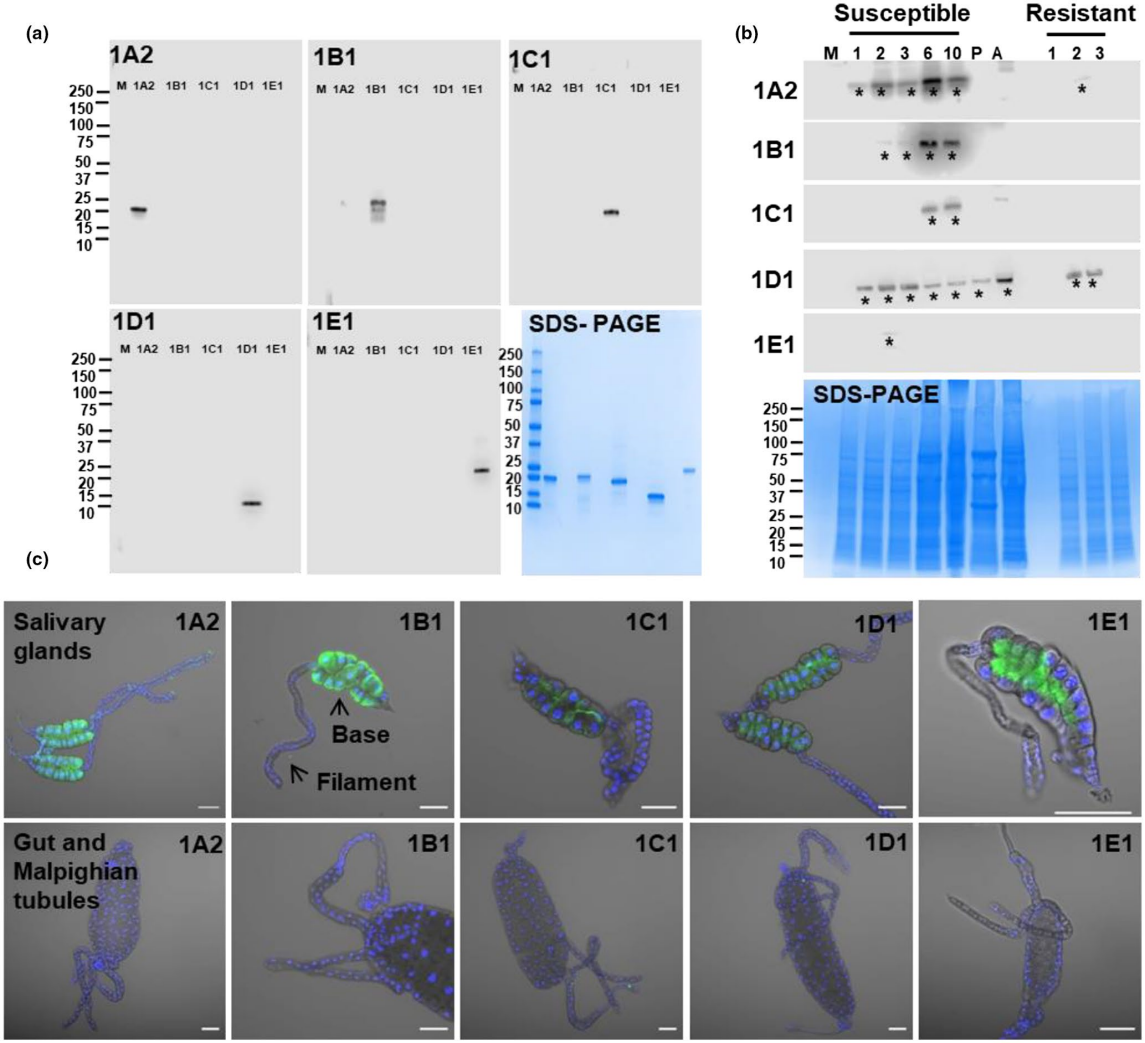
The presence of Family‐1 effectors in different tissues and developmental stages of Hessian fly. (a) The specificity of each antibody to its corresponding antigen. The five recombinant proteins were separated on SDS‐PAGE and transferred onto a nitrocellulose membrane. Individual blots were detected with one of the five antibodies against 1A2, 1B1, 1C1, 1D1, and 1E1. Lanes in each image correspond to molecular markers (M) measured in kDa, and recombinant proteins 1A2, 1B1, 1C1, 1D1, and 1E1 respectively. SDS‐polyacrylamide gel electrophoresis shows the equal loading amount of proteins used for antibody specificity analyses (b). Western blot analyses of extracts from the whole body of insects at different developmental stages. Samples extracted from insects feeding in susceptible plants included first instar larvae on day 1, day 2, and day 3; second instar larvae on day 6 and day 10, puparium (P), and adults (A). Samples extracted from insects feeding in resistant plants included first instar larvae on day 1, day 2, and day 3. No more samples could be obtained at later stages because larvae were dead after 3 days on resistant plants. Bands with expected molecular sizes of each one of the five candidate effectors were marked with an asterisk. Gel images of the SDS page demonstrating equal loadings in each lane for western blots. (c) Indirect immunostaining of different Hessian fly tissues. Salivary glands, gut, and Malpighian tubules were obtained from larvae at different ages, length of dissected larvae ranged between 0.5 mm and 1 mm. Tissues from the ages of larvae that exhibited the highest level of the corresponding protein were presented, day six for 1A2, 1B1, 1C1, 1D1, and day two for 1E1. The immunostaining assays were independently repeated three times for each age. Overlay images were combined results of both antibody and DAPI staining. Other images were derived from immunostaining with individual antibodies as marked on each image. Bars, 50 µm. Confocal images for immunostaining tissues dissected from larvae age 1, 2, 3, 6, and 10 days, and their corresponding controls with a preabsorbed antibody with its respective antigen are given in (Figure S3,1–5)

To determine when Family‐1 putative effectors are expressed in Hessian fly and where they are located among different tissues, specific antibodies were generated against full‐length recombinant mature proteins using the pET28a (+) system (Novagen) (Figure S2). Affinity‐purified antibodies were highly specific to each recombinant protein among the five putative effectors (Figure 1a). Western blots revealed that different Family‐1 members were expressed differently at different developmental stages (Figure 1b). Specifically, SSGP‐1A2 was detected throughout all larval stages with the highest level in 6‐days old larvae fed on susceptible plants, but it was only detected in 2‐days old larvae fed on resistant plants. No protein could be detected in puparium or adults. SSGP‐1B1 was first observed at a low level in 2‐days old larvae, reached the highest level in 6‐days old larvae, and remained detectable in 10‐days old larvae in susceptible plants. No protein bands were detected in larvae that fed on resistant plants. Similar to SSGP‐1A2, no protein could be detected in puparium or adults; however, proteins with unexpected sizes were observed in the sample from adults. SSGP‐1C1 was detected only in 6 to10 days old larvae. SSGP‐1D1 was detected in insects at all stages including puparium and adults. The protein was also detected in 2–3 days old larvae fed on resistant plants. SSGP‐1E1 was only detected in 2‐days old larvae fed on susceptible plants (Asterisk in Figure 1b). The intensities of the western blot bands were largely consistent with the abundance of the corresponding transcripts of the five Family‐1 members as measured by RT‐PCR (Chen, Liu et al., 2010).

### 2.2 Family‐1 proteins are found only in the basal region of salivary glands of actively feeding larvae

Indirect immunostaining was conducted to determine the localization of individual Family‐1 members in insect different tissues. Tissues were obtained from the ages of larvae that exhibited the highest level of the corresponding protein based on western blot results. All effector proteins were localized exclusively in the basal region of salivary glands (Figure 1c) except SSGP‐1E1, which was also detected in a few cells in the foregut (Red circle in Figures S3,3 C5 and S3,4 D5). No signals were detected in other analyzed tissues, including the filamentous region of the salivary gland, the midgut, and Malpighian tubules. Interestingly, even though all five proteins were localized in the basal region cells of salivary glands, different proteins showed different localization patterns within the basal region (Figures 1c; Figure S3). SSGP‐1A2 and SSGP‐1B1 were mainly localized at the peripheral edges of the base region. In contrast, SSGP‐1C1 was localized at the edge (likely the extracellular space) around each cell. SSGP‐1D1 was localized in the middle region of the cells, and SSGP‐1E1 was localized in the central region of the basal portion of the glands. A time‐course analysis revealed that these proteins were present in larvae at ages 1, 2, 3, 6, and 10 days with the highest levels in 3 to 6‐days old larvae (Figure S3).

### 2.3 Family‐1 putative effectors are injected into host tissues

**FIGURE 2:**
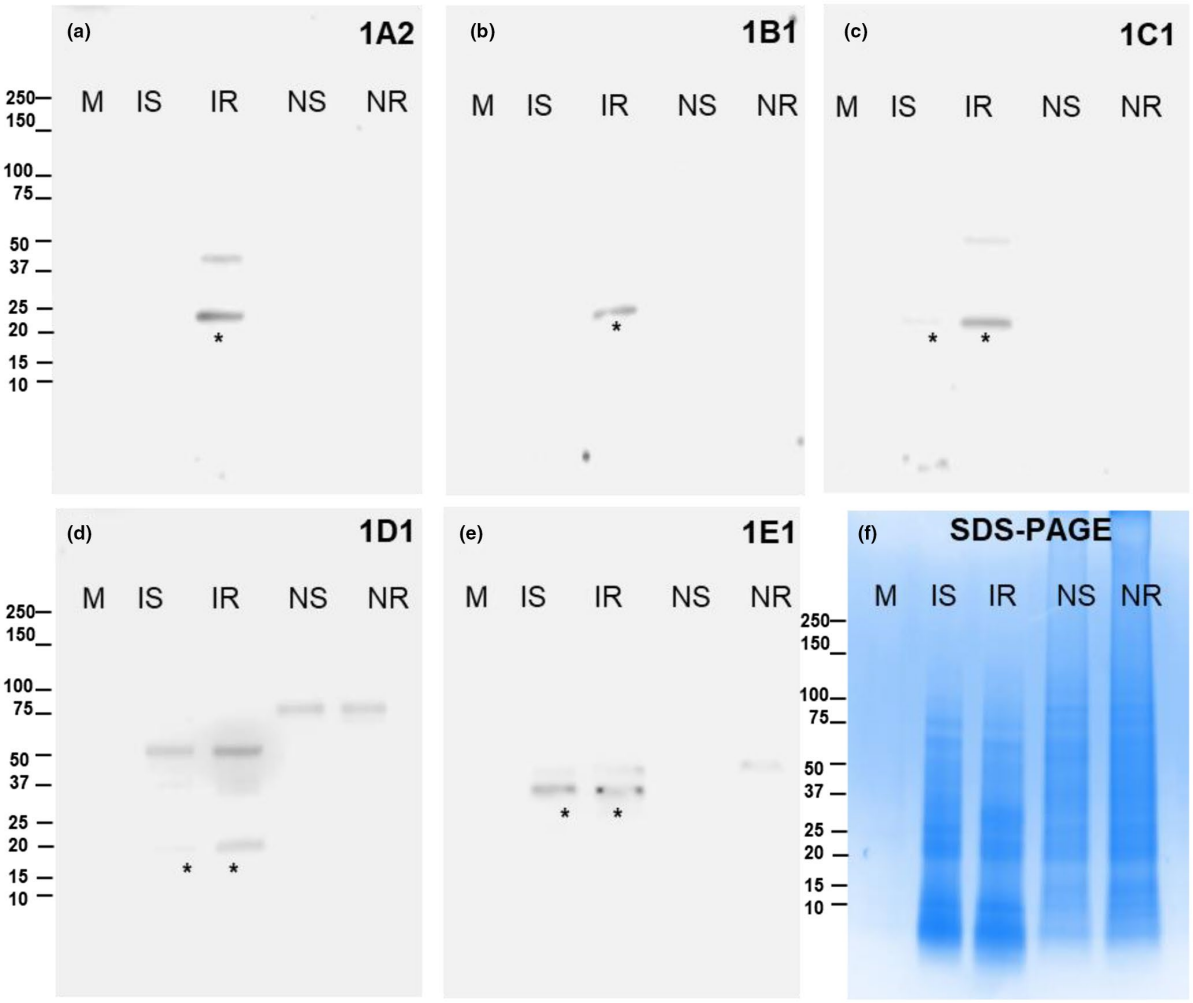
Presence of family‐1 effectors in infested plant tissues. Protein extracts were obtained from infested susceptible and resistant wheat tissues at the feeding site three days after Hessian fly larval infestation. Protein extracts of noninfested susceptible and resistant wheat tissues are also collected as controls. (a–e) western blot analyses of wheat samples obtained from infested resistant plants (IR), infested susceptible (IS), noninfested resistant plants (NR) and noninfested susceptible plants (NS) with antibodies against effector proteins SSGP‐1A2 (a), SSGP‐1B1 (b), SSGP‐1C1 (c), SSGP‐1D1 (d), and SSGP‐1E1 (e) respectively. Bands with expected molecular sizes of each one of the five candidate effectors were marked with an asterisk. (f) A SDS‐polyacrylamide gel electrophoresis shows the equal loading amount of proteins used for the western blot analyses. The letter M represents molecular markers in kDa. Western blots were independently repeated three times for each one of the five candidate effectors

To determine if Family‐1 proteins are injected into wheat tissues by Hessian fly larvae, protein extract from infested and noninfested wheat tissues at the feeding site after removing all Hessian fly larvae were analyzed by western blots. Protein bands with expected molecular weights were detected in samples from infested resistant plants for all five candidate effectors, but only SSGP‐1C1, 1D1, and 1E1 were detected in samples from infested susceptible plants (specific bands are pointed with asterisks in Figure 2). No corresponding bands with expected molecular sizes were detected in either noninfested susceptible plants (NS) or noninfested resistant plants (NR).

### 2.4 Family‐1 effectors spread to host cells surrounding the feeding site in susceptible plants

**FIGURE 3:**
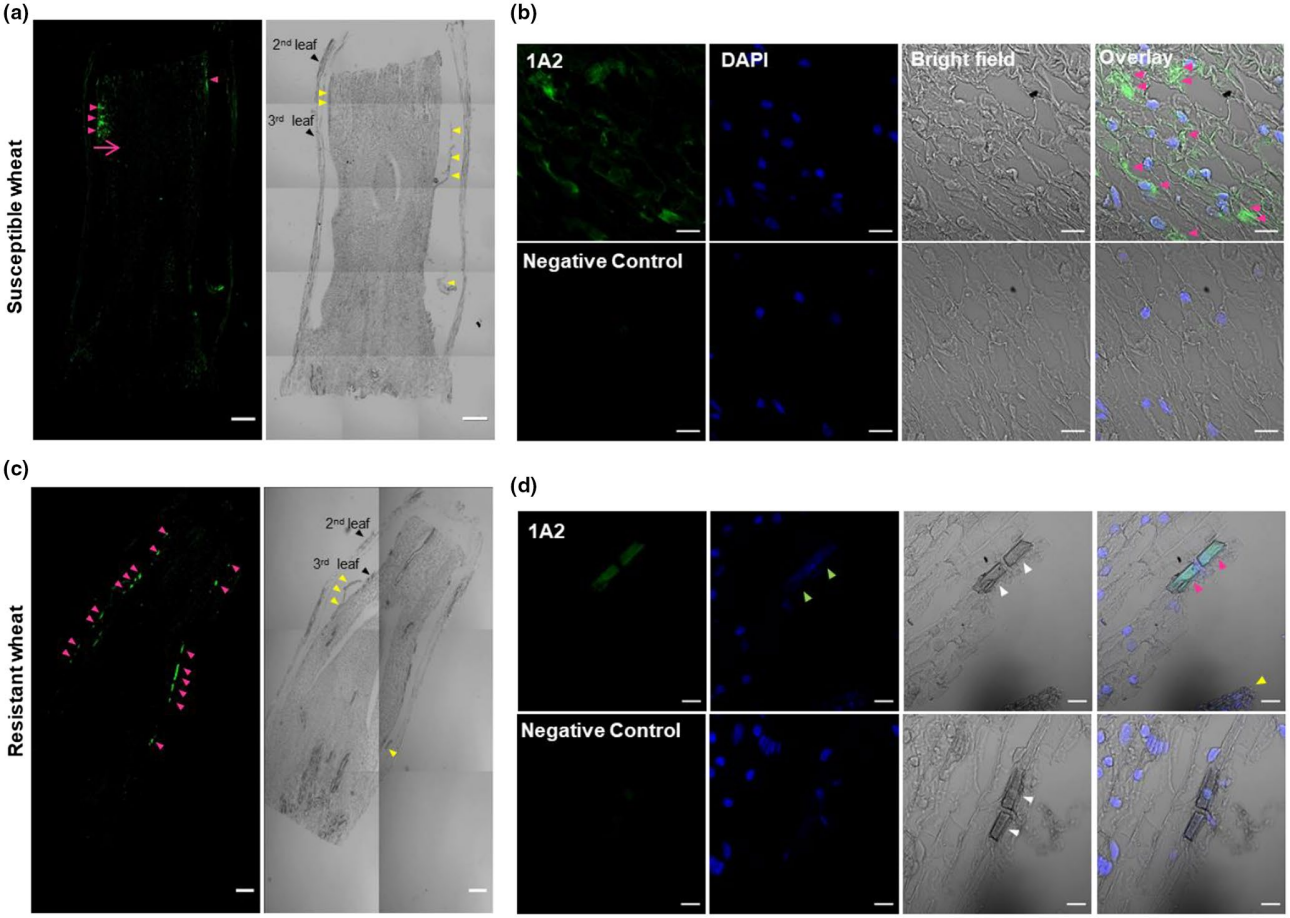
Localization of Family‐1 candidate effectors in wheat tissues three days after Hessian fly larval infestation. Samples of wheat leaf‐sheaths 2–3 mm long at the feeding site with larvae inside were cut to perform immunostaining. Longitudinal paraffin molds were sectioned and stained with the antibody against SSGP‐1A2. (a) and (b) are susceptible plant sections. (a) The whole longitudinal section shows the direction of effector’s penetration. Signal was detected starting from the edge of the attack sheath of the third leaf and spreading into deeper tissues as pointed by a magnate arrow. Larval bodies (yellow arrowheads) were located in the permanent feeding site between the sheaths of the second and third leaves attacking the abaxial side of the third leaf. Magenta arrowheads point to the detected signal at the attack (feeding) sites. Black arrowheads point to the location of the second and third leaves of the wheat stem. Only bright field and antibody staining channels are presented. Bar 200 µm. (b) Magnified image shows scattered signal of 1A2 as pointed by magenta arrowheads in overlay channel. Bar 20 µm. (c) and (d) are resistant plant sections. (c) In the whole longitudinal section, the effector was limited in its localization to the surface of the third leaf‐sheath. (d) In the magnified image, magenta arrowheads point to the signal spots derived from the respective effector. A yellow arrowhead points to the location of the Hessian fly larva. White arrowheads point to dead cells. Green arrowheads point to disappeared nuclei that failed to be stained with DAPI. A preabsorbed antibody with its respective antigen is provided as a negative control in (b) and (d). Immunostaining assays were independently repeated three times for both susceptible and resistant plants. The autofluorescence background in resistant tissues was reduced by quenching protocol. Images were then quantified by subtracting the signal intensity of controls stained with preabsorbed antibody from that of antibody‐stained sections. Data images from five replicates were analyzed and presented in (Figure S5). The remaining antibody staining of family‐1 candidate effectors with their corresponding controls in susceptible and resistant plant sections are presented in (Figures S4,1 and S4,2 respectively). Localizations of family‐1 effectors in resistant lines with resistance genes H6, H21, and H26; and their respective controls are shown in (Figure S4,3–5)

To determine where Family‐1 effectors are located after they are injected into plant tissues, paraffin embedding sections of susceptible wheat plants with and without Hessian fly infestation were stained with each of the five antibodies. As shown in Figure 3a, the effector SSGP‐1A2 was able to penetrate deeper tissues around the feeding site from the edge of the attacked sheath (the direction of effector penetration is pointed by the magenta arrow). After three days, SSGP‐1A2 was dispersed across multiple cells in the surrounding area of the feeding site (representative signals pointed out by magenta arrowheads) (Figure 3b). The same patterns of signal distribution were observed with the other four effectors on immunostaining (Figure S4,1). Typical nuclei were stained with DAPI in infested wheat cells (Figure 3b; Figure S4,1). No signals could be detected in the negative controls nor noninfested plants with any of the five antibodies under the same conditions (Figure 3b, Figure S4,1).

### 2.5 Family‐1 effectors are limited to the originally attacked cells in resistant plants

Similar immunostaining was performed to determine the localization of Family‐1 effectors in resistant plants containing the R gene *H13*. Unlike the dispersed signals observed in infested susceptible plants, strong signals of SSGP‐1A2 (pointed out by magenta arrowheads) were limited to the surface of a few cells at the attack sites (nearby larval bodies are marked by yellow arrowheads) (Figure 3c). Three days after the initial larval attack, SSGP‐1A2 signal became stronger but was only limited to the originally attacked cell and unable to spread into deeper tissues (Figure 3d). The attacked cells with strong signals were dead, showing abnormal structures (white arrowheads) with nuclei that could not be stained with DAPI (green arrowheads). Similar patterns of signal distribution were observed in sections stained with antibodies against the other four Family‐1 members (Figure S4,2). When similar immunostaining was carried out on other resistant plants, each carrying a different resistance gene, including *H6*, *H21*, or *H26*, similar patterns of effector localization were observed (Figures S4,3–S4,5). Again, no signals could be detected in the negative controls nor noninfested plants carrying different resistance genes under the same conditions (Figure 3d; Figures S4,2–S4,5).

### 2.6 Cell death and cell‐wall strengthening are associated with the inability of Family‐1 effectors to penetrate deeper tissues in resistant plants

**FIGURE 4:**
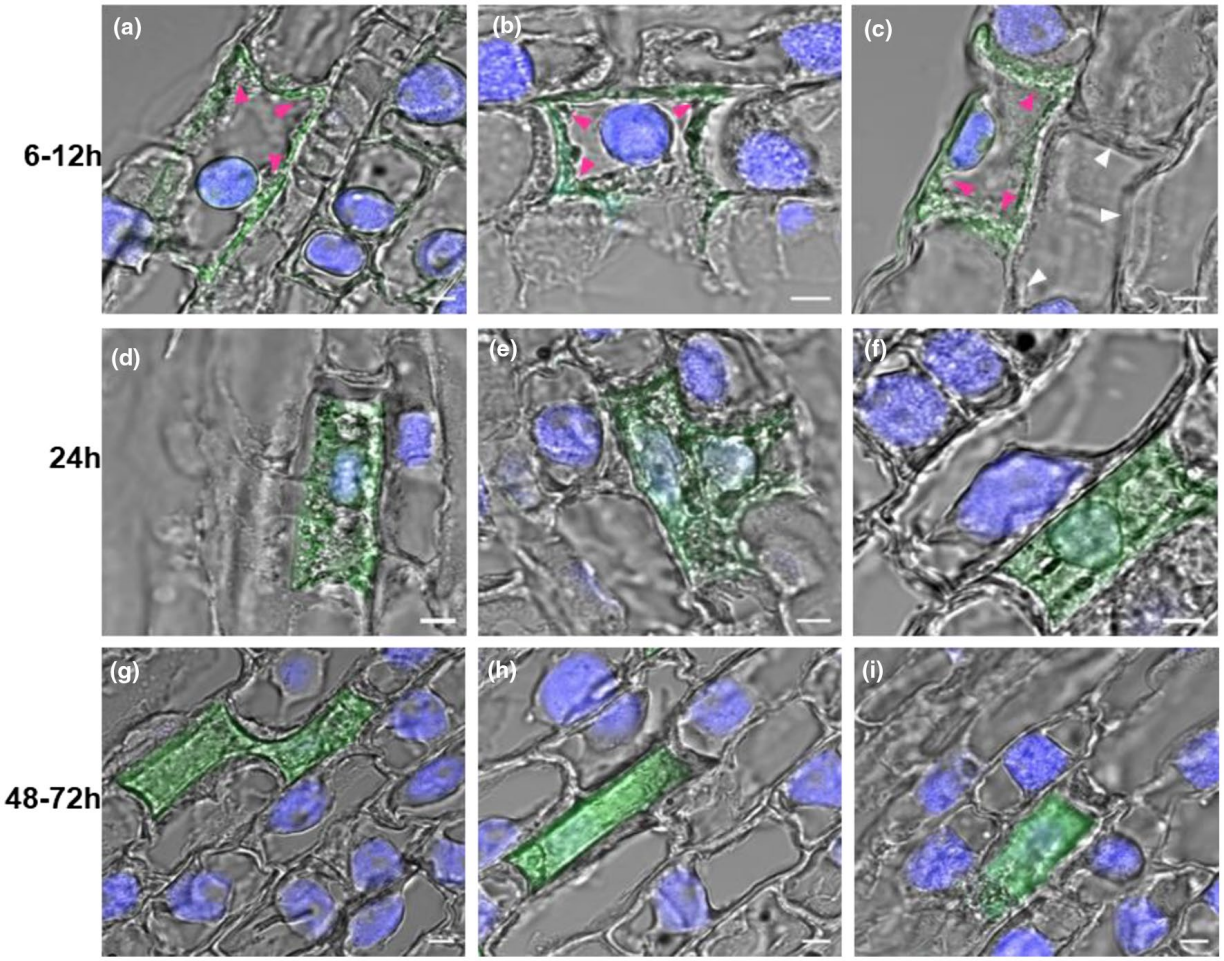
Cell‐wall strengthening and cell death are associated with an inability of Family‐1 effectors to spread to other adjacent cells in resistant plants. Indirect immunostaining of paraffin‐embedded wheat tissue sections at the attack site of resistant plants with an antibody against the SSGP‐1A2 effector was tested in a time course. Graphs (a)–(c) were samples collected at 6h to 12 hr after the initial Hessian fly larval attack. Graphs (d)–(f) were samples collected 24 hr after the initial larval attack. Graphs (g)–(i) were samples collected 48 hr to 72 hr after the initial attack. Bars, 5 µm. Magenta arrowheads are pointing to the effector signal in the extracellular spaces around the attacked cells. White arrowheads are pointing to the walls of un‐attacked adjacent cells

Paraffin‐embedded sections of plant tissues near the attack site collected at different time points after the initial larval attack were immunostained and examined (Figure 4). From 6 to 12 hr after the initial attack, Family‐1 effectors were concentrated in the extracellular spaces (pointed out by magenta arrowheads) around the attacked cells. Essentially no signal was observed in the cytoplasm of the attacked cells nor in un‐attacked cells (Figure 4a–c). The cell wall of the attacked cells became thicker than that of adjacent cells (pointed out by white arrowheads) based on images from the confocal microscopy (Figure 4c). Nuclei of the attacked cells were stainable by DAPI similar to the adjacent nonattacked cells. At 24 hr, effectors had spread all over the attacked cells, and nuclei became shrunk and deformed as revealed by DAPI staining (Figure 4d–f). At 48–72 hr, effectors spread throughout the attacked cells. Nuclei and other subcellular organelles disappeared completely, and there were no signs of cell viability (Figure 4g–i).

**FIGURE 5:**
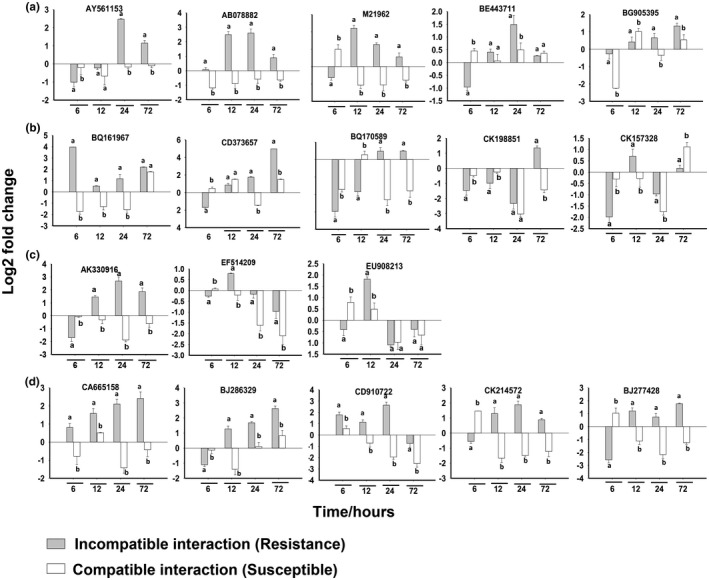
Mechanisms behind cell death in wheat resistant tissues. rt‐PCR results of representative marker genes associated with Hypersensitive response HR and Cell‐wall strengthening. Samples collected at different time points 6, 12, 24, and 72 hr. Data represent means ± SE of three biological replicates, the letters a and b indicate significant differences between the tested groups (*p* ≤ .05, Student’s *t* test). (a) Oxidases genes; Ta. *(Triticum aestivum)* NADPH oxidase (AY561153), Ta. alternative oxidase (AB078882), Ta. oxalate oxidases (M21962), Ta. glycolate oxidases (BE443711), and Ta. amine oxidases (BG905395). (b) Genes of class III peroxide; Ta. peroxidases with accession numbers BQ161967, CD373657, BQ170589, CK198851, and CK157328. (c) Marker genes; 3‐hydroxy‐3‐methylglutaryl CoA reductase Tahsr203 (AK330916), wheat membrane proteins TaHIR1 (EF514209), and TaHIR3 (EU908213). (d) Genes of cell wall fortification; Ta. xyloglucan fucosyltransferase (CA665158), Ta. epicuticular wax synthetase (BJ286329), Ta. xylanase inhibitor (CD910722), Ta. xyloglucan endotransglycosylase (CK214572), and Ta. beta‐1,3‐glucanase (BJ277428)

The death of the attacked cells was likely due to hypersensitive reactions. As shown in Figure 5a, the two hypersensitive response markers, NADPH‐dependent oxidase (AY561153) and alternative oxidase (AB078882), were strongly upregulated specifically in the attacked resistant plants based on RT‐PCR results. Several other genes participating in hypersensitive reactions, including genes encoding oxalate oxidases (M21962), glycolate oxidases (BE443711), and amine oxidases (BG905395) (Figure 5a). Several genes encoding class III peroxidases (BQ161967, CD373657, BQ170589, CK198851, CK157328), were also significantly upregulated in attacked resistant plants, but the expression of these genes were either not changed or downregulated in attacked susceptible plants (Figure 5b). In addition, RT‐PCR revealed that marker genes associated with HR during incompatible plant‐pathogen interactions (Pontier et al., 1994; Yu et al., 2008; Zhang et al., 2009) were also upregulated in resistant wheat infested with Hessian fly (Figure 5c). These markers included 3‐hydroxy‐3‐methylglutaryl CoA reductase, a wheat homolog of gene Tahsr203 (AK330916); and two wheat membrane proteins TaHIR1 (EF514209) and TaHIR3 (EU908213).

Cell‐wall strengthening in attacked cells was likely one of the reasons that Family‐1 effectors did not penetrate adjacent cells. Cell wall was thicker in infested cells than the adjacent noninfested cells a few hours after larvae attack (Figure 4c). Similar to the strong induction of hypersensitive genes, genes encoding xyloglucan fucosyltransferase (CA665158) and epicuticular wax synthetase (BJ286329) were strongly induced specifically in attacked resistant plants (Figure 5d). Both xyloglucan fucosyltransferase and epicuticular wax synthetase are enzymes for the strengthening of cell walls (Khajuria et al., 2013). Other cell‐wall‐related genes, including xylanase inhibitor (CD910722), xyloglucan endotransglycosylase (CK214572) and beta‐1,3‐glucanase (BJ277428), were also induced in attacked resistant plants (Figure 5d).

### 2.7 The Effector SSGP‐1A2 induces cell death and triggers plant defense in *Nicotiana benthamiana*

**FIGURE 6:**
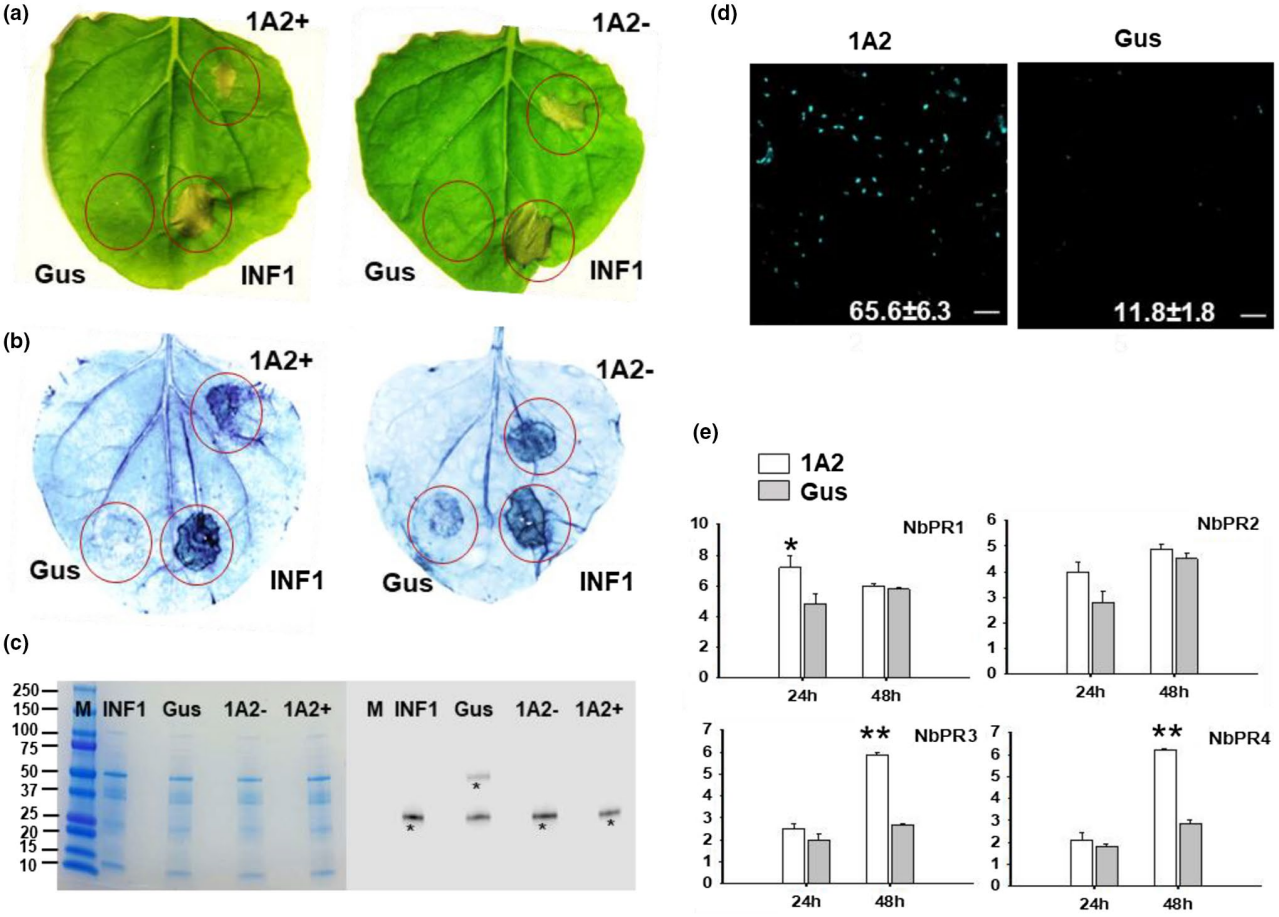
Hessian fly effector SSGP‐1A2 induces hypersensitive reaction (HR) and subsequent cell death in infiltrated *Nicotiana benthamiana* leaves and affects plant defense response. (a) Leaves of *N. benthamiana* were infiltrated with *Agrobacterium tumefaciens* carrying Gus, INF1, 1A2+ mature protein with a signal peptide, and 1A2− without signal peptide. The leaves were photographed 5 days after inoculation. INF1 is a positive control that induces HR in *N. benthamiana* leaves. Gus, B‐glucuronidase, is a negative control that did not induce HR in *N. benthamiana* leaves. The infiltration assay was independently repeated three times, with four plant replicates. (b) Dead cells in the treated leaves were stained with Trypan Blue. (c) Proteins of the target genes expressed in *N. benthamiana* leaves were detected by Western blot. Samples were collected 48 hr after inoculation with Gus, INF1, and 1A2. Bands with expected molecular sizes of each one of the expressed proteins were marked with an asterisk. A SDS‐polyacrylamide gel electrophoresis shows the equal loading amount of proteins used for the Western blot analyses. The letter M represents Molecular marker in kDa. (d) Aniline blue staining of *N. benthamiana* leaves shows callose deposition spots in areas transfected with Gus or 1A2. Photographs were taken 48 hr after inoculation. Numbers indicate means ± SE of the number of callose spots in six individual leaf discs (*p* = 1.89E‐05. Student’s t‐test). Bars, 20 µm. (e) Expression of the defense‐related genes NbPR1, NbPR2, NbPR3, and NbPR4 following transient expression of Gus and 1A2 in *N. benthamiana* leaves. Data represent means ± SE of three biological replicates. Asterisks above the columns indicate significant differences compared with the negative control (*p* < .01, Student’s *t* test)

To test whether Family‐1 effectors can induce hypersensitive responses and subsequent cell death, agroinfiltration‐mediated transient expression of SSGP‐1A2 in *N. benthamiana* leaves was performed (Figure 6). Our results revealed that SSGP‐1A2 (with and without the signal peptide) induced a Hypersensitive response in infiltrated *N. benthamiana* leaves, along with the positive control elicitin INF1 from *Phytophthora infestans* (Ricci et al., 1992). Whereas the negative control, B‐glucuronidase, did not induce any response (Kamoun et al. 2003) (Figure 6a). Six to eight days after infiltration with SSGP‐1A2, cell death was visible at the infiltration sites (Figure S7). Cell death in infiltrated leaves was further confirmed by trypan blue staining (Figure 6b). Significant amounts of callose deposition in cells of SSGP‐1A2 infiltrated leaves were observed based on staining with aniline blue (*p* = 1.89E‐05) (Figure 6d). Several pathogen‐related genes (PR‐genes) were upregulated in response to SSGP‐1A2 infiltration (Figure 6e). Specifically, the expression level of NbPR1 increased 24h after infiltration (*p* = .035), and the expression level of NbPR3 and NbPR4 increased significantly 48h after infiltration (*p* = .00021, and *p* = .00051 respectively). However, the expression level of NbPR2 did not change significantly (*p* = .06).

## 3 DISCUSSION

Despite the considerable effort in studying effectors’ roles in plant–insect interactions, the mechanisms of an effector‐based attack on host plants by parasitic insects are still fragmented. One of the difficulties for studying insect effectors is that it is hard to detect them in host tissues due to the small amounts of insect proteins in host plants (Wang et al., 2018). So far only a handful of effector proteins from insect and pathogen species have been directly visualized in infested plants, including the pea aphid effector Armet in infested *fava bean* (Wang et al., 2015), aphid effectors Mp10, MpOS‐D1, MpC002, and MpPIntO1 in infested *Arabidopsis thaliana* (Mugford et al, 2016), an endo‐β‐1,4‐glucanase and an EF‐hand calcium‐binding protein from the brown planthopper in infested rice plants (Ye et al., 2017; Ji et al., 2017), the low molecular weight whitefly salivary protein Bt56 in tobacco plants (Xu et al., 2019), and the effector AvrRxo1 in rice tissues from *Xanthomonas oryzae* (Shidore et al. 2017).

In this study, we chose the most abundant putative effectors of Hessian fly for the detection of their presence in infested host plants based on our previous study (Chen et al., 2008). We also adopted two different sampling methods for western blots and immunostaining. For western blots, we infested wheat seedlings heavily, with average larval density ~200 Hessian fly larvae per plant. For immunostaining, we infested wheat seedlings at lower density, with an average 5 larvae per plant. With these two different approaches, we were able to detect the presence of Family‐1 effectors in infested wheat on western blots and visualize their localization in wheat tissues via immunostaining.

The fact that all five proteins were either exclusively or predominantly expressed in the base region of salivary glands from actively feeding larvae is consistent with their roles as effector proteins. Based on morphological changes of the salivary glands during Hessian fly development, the basal region is believed to be responsible for producing effectors that are injected into host plants during feeding, whereas the filament region plays roles in internal physiology of the insect (Stuart and Hatchett, 1987). Interestingly, the five Family‐1 effectors were detected in different locations or cells in the basal region, indicating that different candidate effectors have different expression regulatory mechanisms or even different secretion pathways. Similar observations of different effectors in different regions of salivary glands were reported in other insects. For example, two aphid effectors, C002 and Armet, are localized in different subsets of secretory cells of the principal salivary glands, which could facilitate different regulations for expressing different effectors with different roles (Mutti et al., 2008; Wang et al., 2015).

Western blots detected Family‐1 effectors in wheat tissues at the feeding site three days after infestation. This indicates that the Family‐1 proteins were indeed injected into wheat tissues. Interestingly, the five proteins were either at a very low level or absent in the susceptible plant tissues compared with that in resistant plants (Figure 2). This phenomenon might be because effectors were confined in the initially attacked cells in resistant plants as observed in immunostaining (Figure 3c), which facilitated the extraction of the effectors for western analysis under our experimental conditions. In comparison, effectors were spread into deep tissues in susceptible plants and might be harder for the extraction of these proteins for western blots. Alternatively or in addition, some of the effector proteins spreading to plant tissues might have been degraded by the plant defense system. This postulation also explains the inconsistency between very low detected levels of Family‐1 proteins in larvae feeding in resistant plants on western blots and strong signals in infested resistant plant cells visualized by indirect immunostaining (Figures 1b and 3d). Effector proteins might have accumulated to high levels in resistant plant cells without being degraded due to plant cell death, whereas stressed larvae in resistant plants produced fewer effector proteins at later times.

In susceptible plants, effector proteins were initially localized in the attacked cells where a permanent feeding site is established by the first instar larva, and then gradually spread into adjacent cells of deeper tissues around the attack site (Figure 3a,b). The ability of Family‐1 effectors to spread into nonattacked cells, the conservation of the protein sequences among different gall midges (Al‐Jbory, El‐Bouhssini et al., 2018), and the high levels of gene expression in the salivary glands (Chen, Liu et al., 2010) indicate that these effectors play important roles in Hessian fly larval feeding and/or host manipulation. Yet the specific functions of the Family‐1 effectors remain to be revealed.

Hessian fly larvae need to maintain host plants alive to sustain their nourishment. Scanned sections from susceptible plants repeatedly revealed that cells of the infested area maintained their normal appearances with normal DAPI staining of nuclei (Figure 3b; Figure S4,1). Biotrophic pathogens can produce factors to suppress cell death (Panstruga, 2003). Many effector proteins of type III secretion system (TTSS) of phytopathogenic bacteria have been shown to block cell death in host plants like AvrPtoB (Abramovitch et al., 2003) and HopPtoD2 (Espinosa et al., 2003). In the case of Hessian fly, infested susceptible seedlings remain alive without apparent secondary infestation from surrounding microorganisms even though plants are seriously damaged physically due to Hessian fly attack and subsequent feeding (Cartwright et al., 1959). The prolonged‐expression of Family‐1 effectors correlated with the longevity of attacked susceptible plants and larval feeding activity (Figure 1b). When plants die (after Hessian fly larvae do not need to ingest nutrients), all Family‐1 effectors except one are no longer expressed, suggesting possible roles of these effectors in the suppression of host cell death and preventing host cells from secondary infection. Filamentous pathogens secrete waves of effectors during stages of infection to suppress immune recognition and promote cell survival (Toruño et al., 2016).

In contrast with the spread of effector proteins into deeper tissues in susceptible plants, effector proteins were limited to the originally attacked cells in resistant plants carrying different resistance genes (Figures 3c; Figure S4,2–5). The prevention of effectors from spreading was likely due to a combination of cell wall strengthening and subsequent cell death. Shortly, after larval attack thickened walls were observed (Figure 4a–c) around the attack cells, which is consistent with the induction of genes involved in cell wall biosynthesis and fortification (Figure 5d) (William et al., 2011; Zhu et al., 2012; Khajuria et al., 2013). In two to three days, dramatic subcellular changes in the attacked cells suggest apparent death (Figure 4d–i), likely due to hypersensitive reactions (Figures 5a–c and 6 **)** (Liu et al., 2010).

Hypersensitivity elicitation ability of parasite effectors is often verified in the nonhost plant *N. benthamiana* (Naessens et al., 2015; Chen et al., 2018; Shangguan et al., 2018). In this study, temporary expression of the effector SSGP‐1A2 in *N. benthamiana* resulted in moderate elicitation of hypersensitive responses (Figure 6; Figure S7), suggesting that the effector could trigger defense reactions under certain conditions. Consistent with this possibility, several PR genes including Nb‐PR1, Nb‐PR3, and Nb‐PR4 were up‐regulated upon infiltration with SSGP‐1A2. Nb‐PR3 and Nb‐PR4 are known as key components in the Jasmonic acid (JA) defense pathway. Thus, the induction of the JA pathway by a Family‐1 effector may indicate effectors’ involvement in triggering plant resistance. Major gene‐mediated wheat resistance to Hessian fly is also involved in hypersensitive reactions (Liu et al., 2010).

## 4 CONCLUSION

In conclusion, our new approach via confocal imaging presents for the first time visualized evidence to identify Hessian fly effectors from both insect and host tissues. The exclusive presence of Family‐1 effectors in salivary glands supports their putative function as specialized tissues to produce and secret effector proteins (Chen et al., 2008). In infested plant tissues, the striking differences in effector localization between resistant and susceptible plants provide insight into the molecular mechanisms of host resistance and susceptibility to the Hessian fly pest. In attacked susceptible plants, effectors spread to deeper tissues from the feeding site, and presumably interact with wheat proteins to manipulate host plants to favor insect parasitism. In attacked resistant plants, fortification of the cell wall and cell death due to hypersensitive reaction prevent the spread of the effectors to nonattacked cells, thus preventing the plant from being manipulated. Our results provided a foundation and directions for future studies to elucidate detailed molecular steps for fly‐induced susceptibility and resistance in wheat plants.

## 5 MATERIALS AND METHODS

### 5.1 Insect and plant materials

Hessian fly biotype GP, derived from a Kansas population (Chen et al., 2009), was used in this study. The insect population has been maintained on the susceptible wheat cultivar Newton. All insects were maintained on wheat seedlings in growth chambers at 20°C and 12:12 L:D hr (day/night) photoperiod. Salivary glands, guts, and Malpighian tubules were dissected from 1, 2, and 3 days old larvae of the first instar, 6 and 10 days old larvae of the second instar in phosphate‐buffered saline. The dissected tissues were processed immediately for indirect immunostaining.

Two wheat cultivars 'Newton' and 'Molly' were used in most experiments including western blot analyses and indirect immunostaining. Newton is a winter wheat line without a resistance gene to Hessian fly, whereas Molly is a 7‐time‐backcrossing isogenic line of Newton and contains the resistance gene *H13* (Patterson et al 1994). Three other wheat cultivars including ‘Caldwell’, ‘Hamlet’, and ‘KS92WGRC26’ containing Hessian fly resistance genes *H6*, *H21*, and *H26*, respectively, were also used in some experiments as mentioned in the respective sections. Wheat seeds were planted in flats containing PRO‐MIX ‘BX’ potting mix (Hummert Inc.). Wheat seedlings were maintained in a growth chamber programmed at 20°C with a photoperiod of 12:12 (L:D) hr. When wheat seedlings reached the 1.5 leaf stage (stage 11 on Zadoks scales), plants were infested with Hessian fly females by confining the flies in a cheesecloth tent. After 4–5 days, eggs hatched into neonates that migrated into wheat plants. Noninfested plants under the same conditions were used as negative controls. For protein extracts from wheat tissues, plant tissues were collected after three days of larval feeding.

### 5.2 Recombinant protein production and purification

Recombinant proteins for the five Family‐1 candidate effectors, SSGP‐1A2, SSGP‐1B1, SSGP‐1C1, SSGP‐1D1, and SSGP‐1E1, were produced using the expression vector pET system (Novagen, Inc). cDNA inserts encoding only mature proteins were obtained by PCR amplification using the primer sets listed in Table S1. PCR products were digested with respective restriction enzymes NdeI, NheI, and BamHI, re‐purified via agarose gels, and then ligated into pET‐28a. After confirming the right orientation of insert by sequencing, the expression constructs were then transformed into chemically competent cells of the bacterial strain BL21(DE3) (Novagen, Inc).

For recombinant expression, a single colony was inoculated into 2 ml LB medium and incubated at 37°C overnight in a shaking incubator. Bacterial cells were collected by centrifugation, and pellets were resuspended in 2 ml fresh medium, which was then used to inoculate 50 ml medium in Erlenmeyer flask. The culture was incubated in a shaking (250 rpm) incubator at 37°C for four hours, and then one mM of IPTG (Isopropyl‐Beta‐d‐Thiogalactopyranoside) was added to the cultures to induce protein production. Recombinant proteins were analyzed on a 12% precasted SDS polyacrylamide gel (SDS‐PAGE) from Life Technologies.

To isolate and purify recombinant proteins, bacterial cells were lysed using the PopCulture Reagent (Novagen, Inc.). PopCulture Reagent was added to the bacteria culture in a 1:9 ratio for cell lysis. The mixture was then mixed well and incubated for 10 min at room temperature, followed by sonication with a microtip at the settings of power level 2–3, duty 20–30% for 8–10 bursts. Total proteins were then analyzed on SDS‐PAGE, and the level of each recombinant protein was visualized by an extra band at the location of expected molecular size by comparing it with protein extracts with an empty vector under the same conditions. The recombinant proteins were then purified using Ni‐NTA His‐bind resin (Novagen, Inc) following the protocol provided by the manufacturer. Resin‐purified proteins were quantified by a Bradford Protein Assay (Bio‐Rad), and their purity was examined on SDS‐PAGE. The protein solution was dialyzed against 4 liters of phosphate‐buffered saline using dialysis Cassettes (Thermo Scientific Slide‐A‐Lyzer) to remove imidazole. Protein samples were lyophilized using Flexi‐Dry MP Lyophilizer.

### 5.3 Antibody production

For antigen preparation, the His‐tag was removed from the recombinant protein by digestion with the protease Thrombin (Novagen.Inc.) following the procedure provided by the manufacturer. After complete digestion as judged by SDS‐PAGE, thrombin was removed using streptavidin, while His‐tag was separated by passing the mixture through a Ni‐NTA His‐bind resin column. After removing His‐tag, the mature recombinant protein was concentrated, dialyzed, and quantified. Each purified recombinant protein was then sent for antibody production via a commercial contract with GenScript. A rabbit polyclonal antibody was generated against each Family‐1 effector. Each antibody was affinity‐purified with the corresponding antigen. Cross‐reactivity of different antibodies to different recombinant candidate effectors was tested by western blots (Figure 1a).

### 5.4 Immunostaining of insect tissues

Insect tissues including salivary glands, guts, and Malpighian tubules were dissected from Hessian fly larvae at different ages in phosphate‐buffered saline. The tissues were mounted onto concave glass slides. Staining was conducted following the protocol described by Šimo et al. (2009). Briefly, dissected tissues were fixed in 4% paraformaldehyde in PBS overnight. The fixed tissues were washed three times in PBS containing 1% Triton X‐100 (PBST). Tissues were dehydrated in methanol/PBS series 20%, 40%, 60%, 80%, 100% each for 30 min, then incubated in 1xPBS, 0.2% Triton X‐100, 20% DMSO Dimethyl sulfoxide, 0.3 M glycine for 10 min. Tissues were blocked in 1xPBS, 0.2% TritonX‐100, 10% DMSO, 6% M normal goat serum (Sigma) for 60 min. The tissues were then incubated in 1:4,000 dilution primary antibody for 2 days. After several washes with 1xPBS/0.2% Tween‐20 with 10 µg/ml heparin, the tissues were incubated overnight with 1:1,000 dilution secondary antibodies (Alexa fluor 488, Molecular Probes). After three washes with 1xPBS, 0.2% Tween‐20, tissues were mounted in glycerol. Negative controls were included with tissue samples stained with a preabsorbed antibody with its respective antigen under the same conditions.

### 5.5 Protein extraction from insect and plant tissues and western blots

For western blots with insect protein extracts, whole bodies of Hessian fly at different developmental stages were collected and frozen in liquid nitrogen immediately. The frozen insects were ground to powder with a high‐speed electric motor. Equal amounts of ground samples (200 mg) were solubilized into a cold TCA‐2ME‐acetone solution. Protein precipitates were collected by centrifugation. After washing with cold TCA‐2ME‐acetone solution, the protein precipitates were air‐dried and then dissolved into R2D2 buffer containing 7 M urea, 2 M thiourea, 2% 3‐[(3‐cholamidopropyl) dimethyl‐ammonio]‐1‐propane‐sulfonate, 2% N‐decyl‐N,N‐dimethyl‐3‐ammonio‐1‐propane‐sulfonate, 20 mM dithiothreitol, 8 mM Tris(2‐carboxyethyl) phosphine). The samples were stored at −20°C until later use for western blot analyses.

For wheat protein extracts, 10 mm wheat sheaths at the feeding/attack site were collected, and leaf‐sheaths were soaked in 10 ml TE‐SDS buffer (50 mM Tris and 2 mM EDTA, pH 8.0, with 0.1% SDS). Hessian fly larvae that fell into the buffer were removed and the solution containing proteins without any insects was transferred to a new tube. Control samples without infestation were collected in the same way. The solution with proteins was frozen in liquid nitrogen immediately. After several collections following the same way, solutions containing proteins were combined, dialyzed against DI water, lyophilized, and dissolved into sample buffer.

Proteins dissolved in sample buffer were boiled for denaturation. Equal amounts of samples were loaded onto a 12% precasted SDS‐PAGE (Life Technologies). The samples were separated by running the gel at 80 volts for 60 minutes. After separation, proteins on the gel were transferred onto a nitrocellulose membrane using an electric device from Thermo Fisher. After transfer, the membrane was blocked with 5% milk at room temperature for 1 hr, and incubated with an antibody at 1:10,000 dilution in PBST buffer for two hours. The membrane was then washed with 1% PBST for 3 hr with a buffer change every 30 minutes. The membrane was then incubated for 1 hr with an HRP‐conjugated secondary antibody at 1:1,000 dilution (Amersham, GE Healthcare Life Sciences). The membrane was washed again with PBST buffer for 3 hr with a buffer change every 30 minutes. Chemiluminescence was developed using a WesternSure^(R)^ PREMIUM Chemiluminescent Substrate. Images were visualized with a C‐DiGit Blot Scanner (Li‐Cor).

### 5.6 Paraffin embedding sections of wheat tissues and immunostaining

Resistant and susceptible wheat seedlings containing three days old larvae were collected. Approximately 2–3 mm wheat stems at the feeding/attack region along with the inside of Hessian fly larvae were cut and collected. The wheat stems were fixed in 4% paraformaldehyde overnight. Dehydration steps were carried out with 70%, 95%, and 100% ethanol concentrations, and then samples were transferred to chloroform. After that, samples were placed for paraffin penetration overnight. Solidified samples were sectioned using a microtome. Five mm paraffin sections were placed on slides and glued with gelatin. Antibody staining was carried out with the same protocol as described in the section of the immunostaining of insect tissues. Antibodies preabsorbed with respective antigens were used as negative controls under the same conditions. Once the slides became ready for mounting, autofluorescence background from wheat tissues was suppressed by incubating the sectioned slides for 5 minutes with quench reagents from a Vector^®^ TrueVIEW^®^ Autofluorescence Quenching Kit (Vector laboratories). Slides were then freshly scanned using confocal microscopy.

### 5.7 Confocal microscopy and image analyses

Confocal microscopy was performed with Carl Zeiss 700, an inverted microscope outfitted. Oil immersion objective 40x (1.4 NA Oil) used in this study, green and blue fluorescence was observed by excitation at 488 and 405 nm respectively. Images were processed using Zen 2.3 (Blue edition Zeiss).

### 5.8 RNA extraction and real‐time quantitative PCR (qPCR)

Wheat sheaths with approximately 10 mm long at the attack/feeding site were collected and frozen in liquid nitrogen immediately. The frozen tissues were ground and immediately put into TRI reagent™ (Molecular Research Center, Inc.). Total RNA was extracted according to the procedure provided by the manufacturer. RNA quality and integrity were assessed using a Bioanalyzer TapeStation (Agilent Technologies). DNase‐treated RNA was used as the template for cDNA synthesis using an Oligo‐dT primer with a SuperScript™ First‐Strand Synthesis kit (Invitrogen) following the manufacturer’s guidelines. Samples were then treated with RNase H (Invitrogen). cDNA was quantified on a Nanodrop2000c (NanoDrop Technologies) spectrophotometer, and samples were diluted to 20 ng/µl to ensure equal amounts of cDNA template. qRT‐PCR was conducted with an SYBR green kit (Bio‐Rad) on a StepOnePlus™ Real‐Time PCR System (Applied Biosystems). Three biological replications, each with two technical replications, were carried for each qPCR analysis. All PCR reactions were carried out in a 20 µl solution using the following program: 95°C for 10 min for denaturation, 40 cycles with each at 95°C for 30 s, 60°C for 15 s and 72°C for 15 s. At the end of each PCR reaction, a melt curve was generated to confirm a single peak and rule out the possibility of nonspecific product formation. Relative fold‐changes of transcript abundance were calculated using the comparative 2−ΔΔCT method (Livak and Schmittgen, 2001). Primers were designed using Primer3 and were listed in (Table S1). The gene encoding the 60S ribosomal protein L21 (RPL21) was used as an endogenous control.

### 5.9 Infiltration of *Agrobacterium tumefaciens* expressing SSGP‐1A2 on *N. benthamiana* leaves

Constructs expressing the Family‐1 candidate effector SSGP‐1A2 and the positive control INF1 were made using a combination of the entry vector pENTR/D‐TOPO (Invitrogen) and the destination vector pEarleyGate 201 (with the HA epitope tag) together with the LR Clonase reaction enzyme mix (Invitrogen). Specifically, cDNA fragments encoding 1A2 with/without the signal peptide, INF1 cDNA, and cDNA for Gus were first cloned by PCR into pENTR/D‐TOPO. The pENTRY constructs with correct targets 1A2, INF1, and Gus were then recombined in the destination vector pEarleyGate 201 (with the HA epitope tag) using LR Clonase reaction enzyme mix (Invitrogen). After orientation and sequence confirmation, the destination constructs were transformed into *Agrobacterium tumefaciens* GV3101 by electroporation. Positive clones were grown in liquid LB media with 50 µg/ml rifampicin and 50 µg/ml kanamycin. Overnight cultures were harvested, washed three times, and then resuspended in infiltration buffer (10 mM MES, pH 5.7, 10 mM MgCl2, and 150 µM acetosyringone) to 0.5 OD600. Suspension culture was incubated for two hours at room temperature, and then infiltrated into *N. benthamiana* leaves at age 4–5 weeks. Tobacco plants were grown in a growth chamber at 24°C/20°C at 14 hr light‐10 hr dark. Infiltrated leaves were monitored daily for hypersensitive responses and cell death symptoms. Infiltration assays were independently repeated three times, with four plant replicates in each assay. Inoculation was carried out on 2–3 leaves on each plant each time.

To confirm the expression of target proteins, infiltrated leaves were collected 48 hr after agroinfitraion. Leaf pieces were ground to a fine powder in mortar and pestle with liquid nitrogen. About 0.5 mg of the powder was then dissolved into one ml extraction buffer (25 mM Tris‐Cl, 5 mM EDTA, 10 mM DTT, and 1% SDS). The samples were then vortexed for one minute and centrifuged at 2,800 *g* for 15 min at 4°C. Supernatants from these samples were collected and used for western blot analysis. Proteins were detected by immunoblotting using an anti‐HA antibody.

To visualize hypersensitive reaction and cell death, leaves were collected and stained with trypan blue after 72h of infiltration. The collected leaves were boiled for five minutes in a glass beaker containing 50 ml staining solution (10 ml lactic acid, 10 ml phenol, 10 ml glycerol, 10 ml H_2_O and 10 mg trypan blue (Sigma‐Aldrich) with 50 ml 96% ethanol (ratio 1:1)). After the green color vanished completely, the leaves were then transferred to a destaining solution (Lacto‐phenol: ethanol 1:2) for overnight. The leaves were then scanned on a 1200 scanner.

To visualize callose deposition, leaf disks were cut and soaked in 70%, 95%, and 100% ethanol, respectively, for two hours each. The leaf discs were then rehydrated in distill water and stained for one hour in aniline blue solution (0.05% aniline blue in 70 mM K‐phosphate buffer at pH 9). The leaf discs were mounted onto a glass slide in 80% glycerol and scanned on a fluorescence microscope Zeiss 700. Scanned images were analyzed and quantified using the ImageJ software programs.

To analyze defense gene expression after 1A2 infiltration, leaves were collected at 24 and 48 hr after infiltration. The leaves were then ground to fine powder in a mortar with a pestle in liquid nitrogen. Total RNA isolation and RT‐PCR were conducted as described previously. Plant defense‐related genes tested in this study included Nb‐PR1, Nb‐PR2, Nb‐PR3, and Nb‐PR4. Nb‐Actin was used as an endogenous control. Primers used were listed in Table S1. Each assay was repeated three times.

## CONFLICTS OF INTEREST

The authors declare no conflicts of interest.

## AUTHOR CONTRIBUTIONS

M.S.C. and Z.A. conceived the research; M.S.C. and G.R.R. supervised the experiments; Z.A. performed the experiments and analyzed the data; M.J.A. and Y.P. provided technical assistance to Z.A.; Z.A. and M.S.C. wrote the article with contributions of all the authors; M.S.C. agrees to serve as the author responsible for contact and ensures communication. This work is joint effort between USDA‐ARS and Kansas State University.

## Supporting information

Fig S1‐S7‐Table S1
