# Supplementary material for: Differential localization of Hessian fly candidate effectors in resistant and susceptible wheat plants
Source: Plant Direct. 2020 Aug 14;4(8):e00246. doi: 10.1002/pld3.246 (PMC7428492; doi:10.1002/pld3.246)
Supplement: Supplementary file 1 — Fig S1‐S7‐Table S1 [file PLD3-4-e00246-s001.pdf]

Figure S1. Sequence alignment of members of SSGP-1 family.

A: An amino acid sequence alignment. The predicted cut site for the signal peptide is indicated by an arrow. Black shaded residues are identical ones while gay shaded residues are similar in nature.

```
SSGP_1D1    1 MSKFLLAFAVIAVCLVAAQAGETDHSADKHG---PGN-----------------------
SSGP_1C2    1 MSKFLLAFAIIAVCLVAAQAAVHS----SHSTAAHAG-----------------------
SSGP_1C1    1 MSKFFLAFAIIAVCLVAAQA-VHS----SHSTAAHAG-----------------------
SSGP_1A1    1 MSKFLLAFAVIAVCLVAAQAAVTKHPAGKKSPAKPAS-----------------------
SSGP_1A2    1 MSKFLLAFAVIAVCLVAAQAAVTKHPAGKKSPAKPAS-----------------------
SSGP_1B1    1 MSKFLLAFAVIAVCLVAAQAA--KPKKGKKPAAAPAG-----------------------
SSGP_1E1    1 MSKFFLAFAVIAVCLVAAQAVQEPQASLPQPHSAPASLPQPHSAPLHQPAPQTVHSSSAS

SSGP_1D1   35 -------------------------------------------ANHAAKKPHTAQPSTYD
SSGP_1C2   34 -------------------------------------------NIH--AATHTAQLSLAD
SSGP_1C1   33 -------------------------------------------KTHEPAKTQTAQPGVGD
SSGP_1A1   38 -------------------------------------------PTH--AAAPTAQSNPDD
SSGP_1A2   38 -------------------------------------------PTH--AAAPTAQSNPDD
SSGP_1B1   36 -------------------------------------------PT----SDPAVEPSSGD
SSGP_1E1   61 LRWDQLAQPAGGYRGMPTRTEPPKTRGDGSSTTTIAKKCRHGNAPSAVAAPYTNQHSLGE

SSGP_1D1   52 F-LDIGEDFADA----WAVL---RGNLGGQNG----AGKPAVAS--------ASAGKIGN
SSGP_1C2   49 D-LDEDEHWFDASDKDFESYFEDDNG-------------PNEA------AVPA----GKP
SSGP_1C1   50 H-LDDGEHYFDASDKDFESYFEDDNE-------------PNEA------AVPA----GKP
SSGP_1A1   53 Q-FD------DLSMLDWEAILADDSDFGNSNG----PATPAAA--------PAKPSKGKP
SSGP_1A2   53 Q-FD------DLSMLDWEAILADDSDFGNSNG----PATPAAA--------PAKPSKGKP
SSGP_1B1   49 L-SD------DQTI-NWDALLAADSNAGDANGLDEAATKPTAAKGPKTAGKPAKPSKAKP
SSGP_1E1  121 YSVEDDEDWYDALDENLESL-------------------PRNVNGHHEAAKP-------P

SSGP_1D1   92 QKTASPPHGHASPTKPAPHKK
SSGP_1C2   85 TK---PLKAKPSPHGKGHQKK
SSGP_1C1   86 TK---PLKAKTSPKGKGHQKK
SSGP_1A1   94 KKADSPKSPKPAPKKAASKKK
SSGP_1A2   94 KKADSPKSPKPAPKKAASKKK
SSGP_1B1  101 KKADSPKKAKPAPKKK-----
SSGP_1E1  155 NTGKHQATGSSKPHGQAKHRK
```

B: A nucleotide sequence alignment of transcripts

```
SSGP-1B1      ACAGTTCAATTCGAACCATTAATTCTTTCAATATCCACTGGAACATCCAA-AAACGAAAA   59
SSGP-1D1      ACAGTTCGAATTGAGCCGTTAATTCTTTCAATATCCACTGGAACATCCAA-AAACGAAAA   59
SSGP-1A2      ACAGTTCAATTCGAACCATTAATACTTTCAATATCCACTGGAACATCCAA-AAACGAAAA   59
SSGP-1A1      ACAGTTCAATTCGAACCATTAATACTTTCAATATCCACTGGAACATCCAA-AAACGAAAA   59
SSGP-1E1      ACAGTTCAATTCGAACCATTAATTCTTTCGATATCCACTGGAACATCCAA-AAACGAAAA   59
SSGP-1C1      ACAGTTCCAATTGAACCGTTTATTCTTTCGATATCCACTGGAACATCCAA-AAACGAAAA   59
SSGP-1C2      ACAGTTCGAATTAAACCGTTAATTCTTTCAATATCCACTGGAACATCCAAAAAACGAAAA   60
              ******* * *  * ** ** ** ***** ******************** *********

SSGP-1B1      TGTCAAAATTTTTACTAGCTTTCGCCGTCATCGCCGTCTGCCTTGTCGCAGCTCAGGCTG   119
SSGP-1D1      TGTCAAAATTTTTACTAGCTTTCGCCGTCATCGCTGTCTGCCTTGTTGCAGCTCAGGCTG   119
SSGP-1A2      TGTCAAAATTTTTACTAGCTTTCGCCGTCATCGCCGTCTG--------------------   99
SSGP-1A1      TGTCAAAATTTTTACTAGCTTTCGCCGTCATCGCCGTCTGCCTTGTTGCAGCTCAGGTAA   119
SSGP-1E1      TGTCAAAATTTTTCTTAGCTTTCGCCGTCATCGCCGTCTGCCTTGTAGCAGCTCAGGCTG   119
SSGP-1C1      TGTCAAAATTTTTCTTAGCTTTCGCCATCATCGCCGTCTGCCTTGTTGCAGCTCAGGCTG   119
SSGP-1C2      TGTCAAAATTTTTATTAGCTTTCGCCATCATCGCCGTCTGCCTTGTTGCAGCTCAGGCTG   120
              *************  *********** ******* *****
```

```
SSGP-1B1      CTAAACCTAAAAAAGGCAAAAAGCCCGCAGCTGCACCGGCAGGTCCAACCTCTGATCCAG  179
SSGP-1D1      GAGAAACAGATCATTCAGCAGACAAACATGGTCCGGGAA---------------------  158
SSGP-1A2      ------------------------------------------------------------  99
SSGP-1A1      ATAACATAAAATTTTCAACACTCATTTTATTGTGTGGCTGGATTCC-ACTTTGGTTTCTT  178
SSGP-1E1      TACAGGAACCACAAGCATCGCTTCCCCAACCACATTCTGCACCAGCATCGCTTCCCCAAC  179
SSGP-1C1      T---ACACAGCAGCCATTCCACAGCTGCACACGCTGGTAAAACCCATGAGCCAGCAAAAA  176
SSGP-1C2      CTGTACACAGCAGCCATTCCACAGCTGCACACGCAGGAAATATCCA------TGCAGCAA  174

SSGP-1B1      CAGT--------------------CGAG-CCAA-------------GCTCAGGTGACCTT  205
SSGP-1D1      ------------------------------------------------------------  158
SSGP-1A2      -----------------------------------------------------CCTTGTT  106
SSGP-1A1      CCCTGTC------ATTTCTCCAATTTAA-TTAAAAT---------CTATCAATTTATTTA  222
SSGP-1E1      CACATTCTGCACCACTTCACCAACCAGCACCGCAAACAGTCCATTCTAGCTCCGCATCGT  239
SSGP-1C1      CGCAAACAGCCCAACCAGGC-------------------------GTAGGTGA-------  204
SSGP-1C2      CGCATACAGCCCAACTAAGC-------------------------CTAGCTGA-------  202

SSGP-1B1      T-----CTGATGACCAAACCAT--------------------------------------  222
SSGP-1D1      ------ATGCAAACCATGCTGCAAAGAAAC---------------CGC---A--------  186
SSGP-1A2      G-----CAGCTCAGGCTGCTGTAACTAAACATCCAGCAGGAAAAAAGT---CCCCAGCTA  158
SSGP-1A1      T-----TTCATAAGGCTGCTGTAACTAAACATCCAGCAGGAAAAAAGT---CCCCAGCTA  274
SSGP-1E1      TGAGGTGGGATCAGCTTGCGCAACCGGCAGGTGGCTATAGAGGAATGCCAACGAGGACTG  299
SSGP-1C1      ------------------------------------------------------------  204
SSGP-1C2      ------------------------------------------------------------  202

SSGP-1B1      ------AAATTG-------GGATGCACTTT---TAGCTGCCG------------------  248
SSGP-1D1      ----------------------------------CACAGCCC------------------  194
SSGP-1A2      AACCAGCAAGTCCAACCCATGCAGCAGCGC---CAACAGCCC------------------  197
SSGP-1A1      AACCAGCAAGTCCAACCCATGCAGCAGCGC---CAACAGCCC------------------  313
SSGP-1E1      AACCACCGAAAACAAGAGGGGACGGATCATCAACAACAACAATCGCAAAAAAATGTCGTC  359
SSGP-1C1      ------------------------------------------------------------  204
SSGP-1C2      ------------------------------------------------------------  202

SSGP-1B1      ---------------------------------ACTCAAATGCGGGAGATGCT-------  268
SSGP-1D1      ---------------------------------AACCAAGCACATATGACTTTCTTGACA  221
SSGP-1A2      ---------------------------------AATCAAACCCAGATGACCAATTTGACG  224
SSGP-1A1      ---------------------------------AATCAAACCCAGATGACCAATTTGACG  340
SSGP-1E1      ACGGAAATGCACCCAGTGCTGTAGCAGCACCGTACACAAACCAACATAGCCTAGGGGAAT  419
SSGP-1C1      ------------------------------------------------------------  204
SSGP-1C2      ------------------------------------------------------------  202

SSGP-1B1      -----AATGGGCTTGATGAAGCGGCAACGAAACC------------------GACAGCCG  305
SSGP-1D1      TAG---GCGAGGACTTTGCAGACGCATGGGCAGTACTTCGCGGAAATTTAGGAGGGCAGA  278
SSGP-1A2      ATCTTTCCATGCTAGATTGGGAGGCTATTCTAGCAGACGATTCAGATTTCGGAAATTCTA  284
SSGP-1A1      ATCTTTCCATGCTAGATTGGGAGGCTATTCTAGCAGACGATTCAGATTTCGGAAATTCTA  400
SSGP-1E1      ATTCTGTCGAAGATGATGAGGACTGGTACGACGCATTGGACGAAAATTTGGAATCACTTC  479
SSGP-1C1      -TCATCTTGACGATGGTGAGCACTATTTCGACGCATCGGACAAAGATTTCGAATCATATT  263
SSGP-1C2      -CGATCTTGACGAAGATGAGCACTGGTTCGACGCATCGGACAAAGATTTCGAATCATATT  261
                              *
SSGP-1B1      CAAAAGGACCAAAGACGGCCGGAAAACCAGCTAAACCATCAAAGGCAAAACCAAAGAAGG  365
SSGP-1D1      ATGGAGCAGGCAAACCGGCAGTTGCATCAGCTTCAGCTGGCAAAATTGGAAACCAAAAGA  338
SSGP-1A2      ATGGACCTGCCACACCAGCAGCTGCACCAGCTAAACCATCAAAAGGAAAACCAAAGAAGG  344
SSGP-1A1      ATGGACCTGCCACACCAGCAGCTGCACCAGCTAAACCATCAAAAGGAAAACCAAAGAAGG  460
SSGP-1E1      CCCGAAATGTTAATGGGCATCATGAAGCAGCCAAAC------------CACCCAATACTG  527
SSGP-1C1      TCGAAGATGATAATGAGCCTAATGAAGCAGCCGTAC------------CGGCAGGAAAAC  311
SSGP-1C2      TCGAAGATGATAATGGCCCTAATGAAGCAGCCGTAC------------CGGCAGGAAAAC  309
                  *      *             * ****   *                     *
SSGP-1B1      CCG---------------ATTCACCAAAAAAAGCAAAACCAGCCCCAAAGAAGAAGTGAT  410
SSGP-1D1      CGGCCTCACCACCACATGGGCACGCATCACCAACAAAGCCAGCCCCACATAAAAAGTGAT  398
SSGP-1A2      CCGATTCACCAAAATCACCAAAACCAGCCCCAAAAAAAGCAGCCTCAAAGAAGAAGTGAT  404
SSGP-1A1      CCGATTCACCAAAATCACCAAAACCAGCCCCAAAAAAAGCAGCCTCAAAGAAGAAGTGAT  520
SSGP-1E1      GAAAACACCAGGCGACCGGATCATCAAAACCACATGGGCAAGCAAAACATAG--------  579
SSGP-1C1      CAACAAAACCATTGAAGGCCAAAACATCGCCAAAAGGAAAAGGCCACCAAAAAAAATGAT  371
SSGP-1C2      CAACAAAACCATTGAAGGCCAAACCATCGCCACATGGAAAAGGCCACCAAAAAAAGTAAT  369
```

```
                                      **     *        **      * *
SSGP-1B1      CATTTCATTCAATTGAAAGAACATTCGGAGACGTCGTGT--AATCAAAATTAAATAGTTA  468
SSGP-1D1      CATTTCATTCAATTGAAAGAACATTTGGAGACGTCGTGT--AACCAAAATTAAACATTTA  456
SSGP-1A2      CATTTCATTCAATTGAAAGAACATTCGGAGACGTCGTGT--AATCAAAATTAAATAGTTA  462
SSGP-1A1      CATTTCATTCAATTGAAAGAACATTCGGAGACGTCGTGT--AATCAAAATTAAATAGTTA  578
SSGP-1E1      ----------------------------------AAAGTGATCATTTCATTTAATTGAAA  605
SSGP-1C1      CATTTCATTCAATTGAAAGAACATTTGGAGGCGTCGCGTGTAACCAAAATTATATAGTTA  431
SSGP-1C2      CATTTCATTCAATTGAAAGAACATTTGGAGGCGTCGCGTGTAACCAAAATTAAATAGTTA  429
                                                   **         ***  *     *
SSGP-1B1      TACGCTCTCATATTTTCAATTTGTCAATTGAAATCAATTCTTGAGATAAAATTCTTGAAT  528
SSGP-1D1      TATGCTCTCATATTTTCAATTTGTCAATTTAAATCAATTCTTGCAATTAAATTTTTGAAT  516
SSGP-1A2      TACGCTCTCATATTTTCAATTTGTCTATTGAAATCAATTCTTGAGATTAAATTCTTGAAT  522
SSGP-1A1      TACGCTCTCATATTTTCAATTTGTCTATTGAAATCAATTCTTGAGATTAAATTCTTGAAT  638
SSGP-1E1      GTACATCTCATATTTTCAAATTGTCCATTGAAATCGATTCTTGCAATTAAATTCTTGAAT  665
SSGP-1C1      TACACTCTCATATTTTCAATTTGTCTATTGAAATCAATTCTTGAAATTAAATTCTTGAAT  491
SSGP-1C2      TATGCTCTCATAAATTCAATTTGTCAATTGAAATCAATTCTTGAAATTAAATTCTTGAAT  489
                   *******  ***** ***** *** ***** *******  ** ***** ******
SSGP-1B1      GTAAAAAAAAAAAT----------AATAATTGAGTATTTTATTCAGAACAAT-CTCCCCC  577
SSGP-1D1      GTAAAAAAAAAAAT----------T------GAGTATTTTATTCAGAACAATCTCCCCTC  560
SSGP-1A2      GTAAAAAAATAAAT----------AATT---GAGTATTTTATTCAGAAAAATC-TCCCCC  568
SSGP-1A1      GTAAAAAAATAAAT----------AA----------------------------------  654
SSGP-1E1      GGAAA---------------AAAAAATAATTGAGTATTTTATTCAGAACGATCT-CCACC  709
SSGP-1C1      GTAAAAAAAAAA------------AATAATTGAGTATTTTATTCAGAACAATCT-CCACC  538
SSGP-1C2      GTAAAAAAAAAAATAAAATAAAATAATAATTGAGTATTTTATTCAGAACGATCT-CCACC  548
              * ***
SSGP-1B1      GTTCTTATCCATCCATAGGTAATCTTTTTAGTT------ATCATTTTGTTAAAATCATGA  631
SSGP-1D1      ATTCTTATCCATCCATAGGTAATCTATTTAGTT------ATTATTTTGTTCAAATCATGA  614
SSGP-1A2      ATTTTCATTCTTCCATAGGTAATCTCTTTAGTT------ATTATTTTGTTCAAATCATGA  622
SSGP-1A1      ------------------------------------------------------------  654
SSGP-1E1      ATTGTTATTCATCAATAGGTAATCTCTTTAGGGTAATCTTTTTAGGTGTTTAAATCATGT  769
SSGP-1C1      ATTCTTATTCATCCATAGGTAATCT------CTTTAGTTATAATTTTGTACAAATCATGA  592
SSGP-1C2      ATTCTTATTCTTCCATAGGATAAGGTCTTTCCTTTAGTTGTTATTTTGTTCAAATCATAA  608

SSGP-1B1      ACAAGTTCGAGAATCT    647
SSGP-1D1      ACAAGTTCGATGATTT    630
SSGP-1A2      ACAAGTTCAAGAATTT    638
SSGP-1A1      ----------------    654
SSGP-1E1      ACAAGTTCCAATATTT    785
SSGP-1C1      ACAAGTTCGATGATTA    608
SSGP-1C2      ACAAGTTCGAAAATTT    624
```

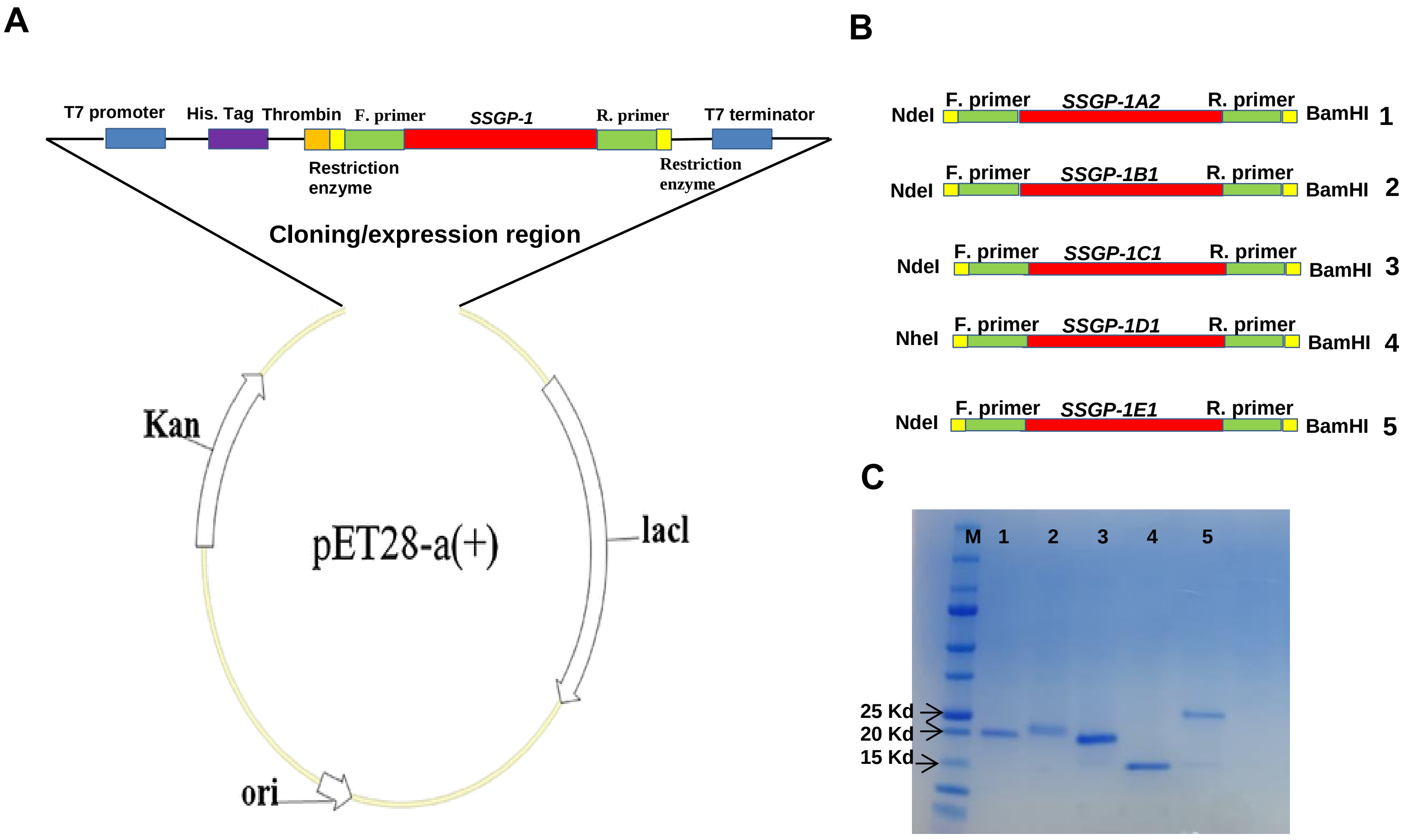

Figure S2. The expression system and strategy for production of recombinant proteins. **Panel A.** The pET28a (+) vector was used for recombinant protein production. The vector uses the T7 promoter to drive transcription of inserts coding for recombinant proteins, and the T7 terminator for preventing transcription beyond the insert. Two selection markers are present in this vector, Kanamycin for antibiotic selection and β-galactosidase (lac) for white/blue colony selection. Three restriction enzyme sites (Bam HI, NdeI, NheI) in the poly-linker were used for cloning inserts. Each recombinant protein carries a His tag at the N-terminal. **Panel B.** Insert and cloning sites. Each insert was PCR amplified with a primer pair listed in Table S1. Each primer carried a unique restriction site for insertion. PCR products for each gene were double-digested with the restriction enzymes listed on the two ends of the insert. The double-digested insert was ligated into the vector that was also double-digested with the same two restriction enzymes. **Panel C.** Purified recombinant proteins on an SDS-PAGE. Lanes 1-5 were SSGP-1A2, SSGP-1B1, SSGP-1C1, SSGP-1D1, and SSGP-1E1 respectively. M represents the Molecular markers.

Figure S3. Localization of Family-1 effectors in different tissues at different larval ages detected through immunostaining.

S3,1: Salivary glands, gut, and Malpighian tubules were obtained from **one day** old larvae. Control represents tissues stained with pre-absorbed antibody with its respective antigen. Individual channels for each one of these dissected tissues with their corresponding controls are displayed. The green color indicates signals from antibody staining. The blue indicates DAPI staining for nucleus. Overlay indicates combined image of both antibody and DAPI staining, A1 to A5 are tissues staining with five different antibodies as indicated above each picture. Bars, 20µm.

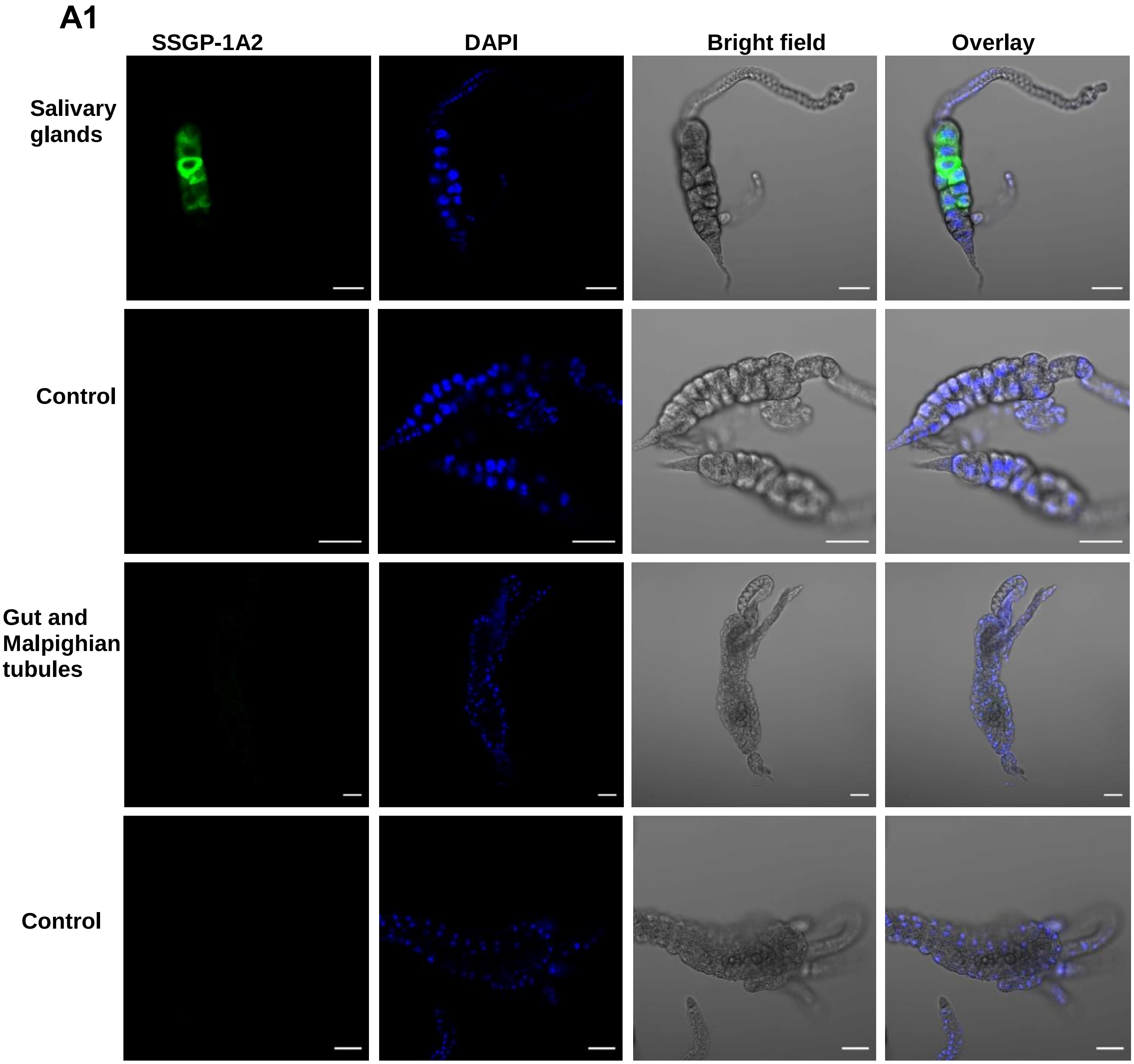

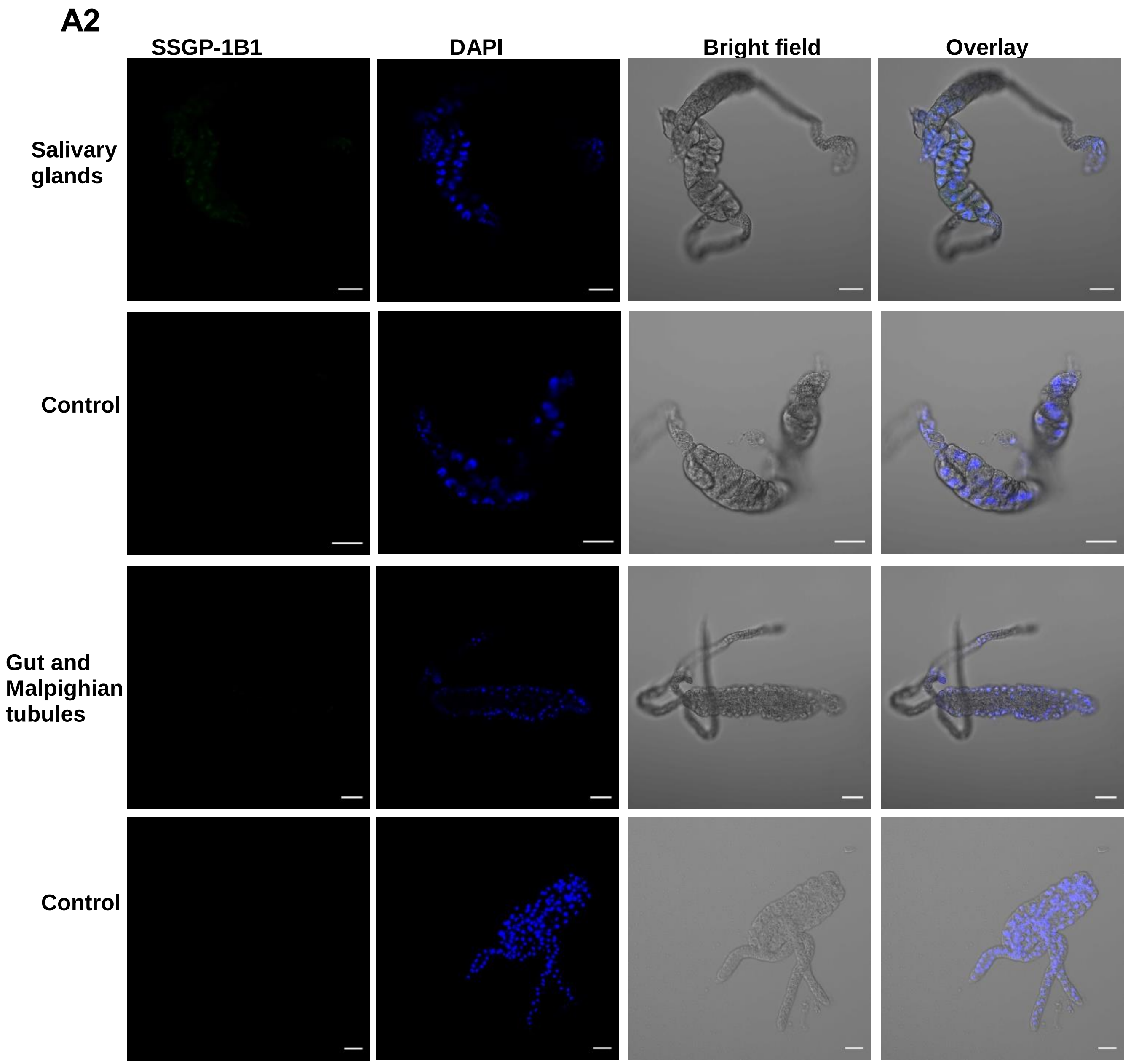
A2
SSGP-1B1
DAPI
Bright field
Overlay
Salivary glands
Control
Gut and Malpighian tubules
Control

A3

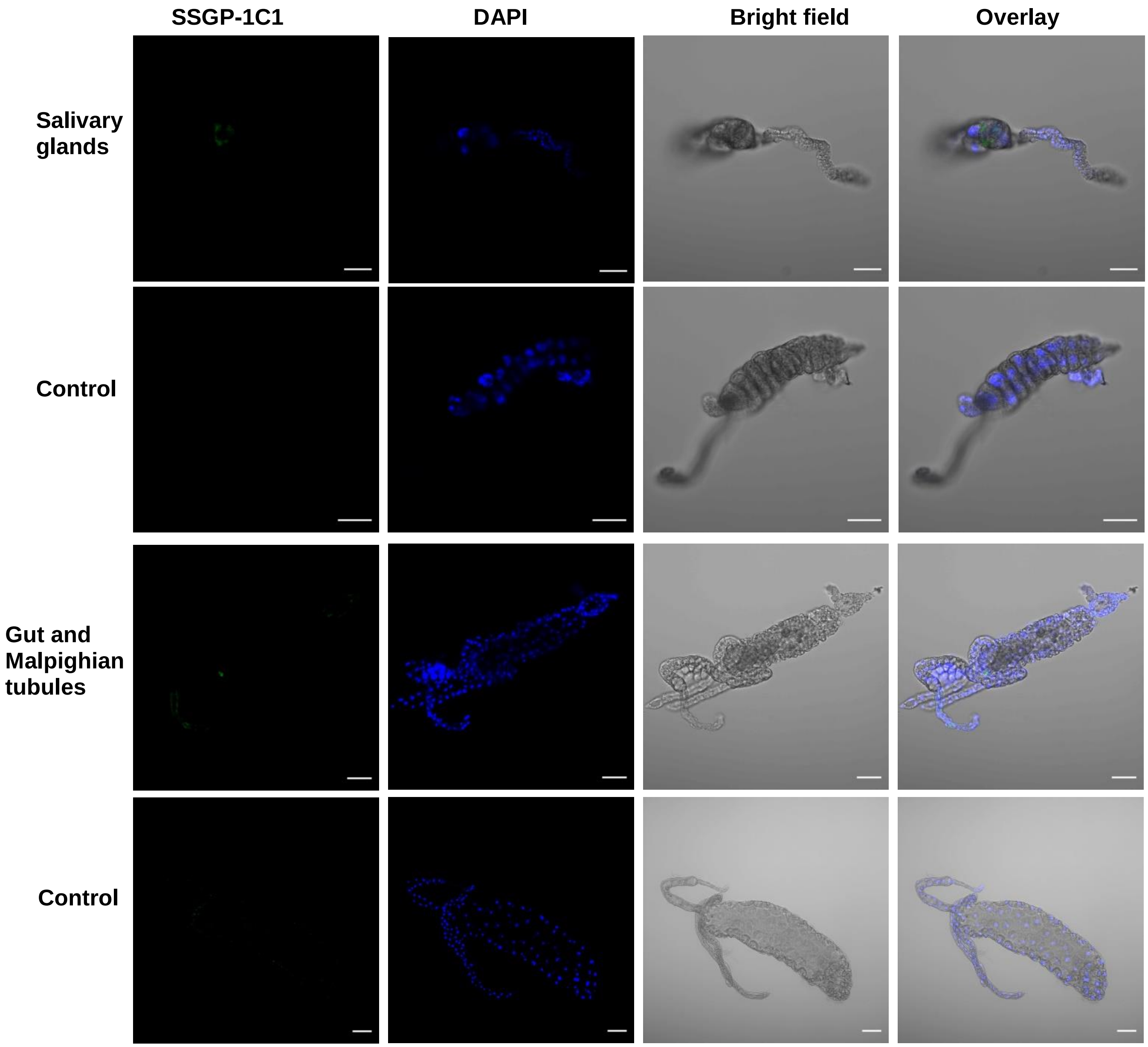
SSGP-1C1
DAPI
Bright field
Overlay
Salivary glands
Control
Gut and Malpighian tubules
Control

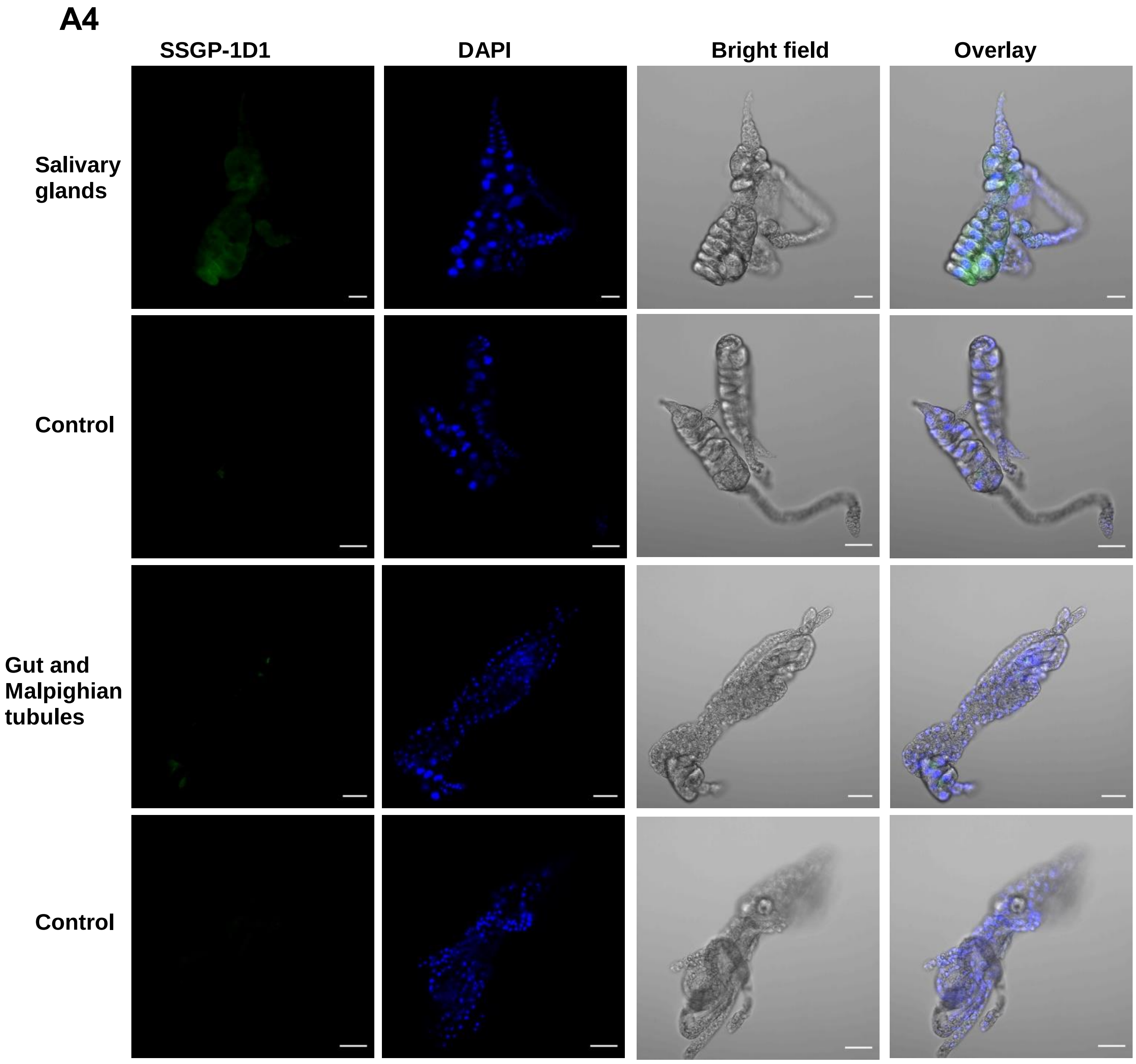

A4
SSGP-1D1
DAPI
Bright field
Overlay
Salivary glands
Control
Gut and Malpighian tubules
Control

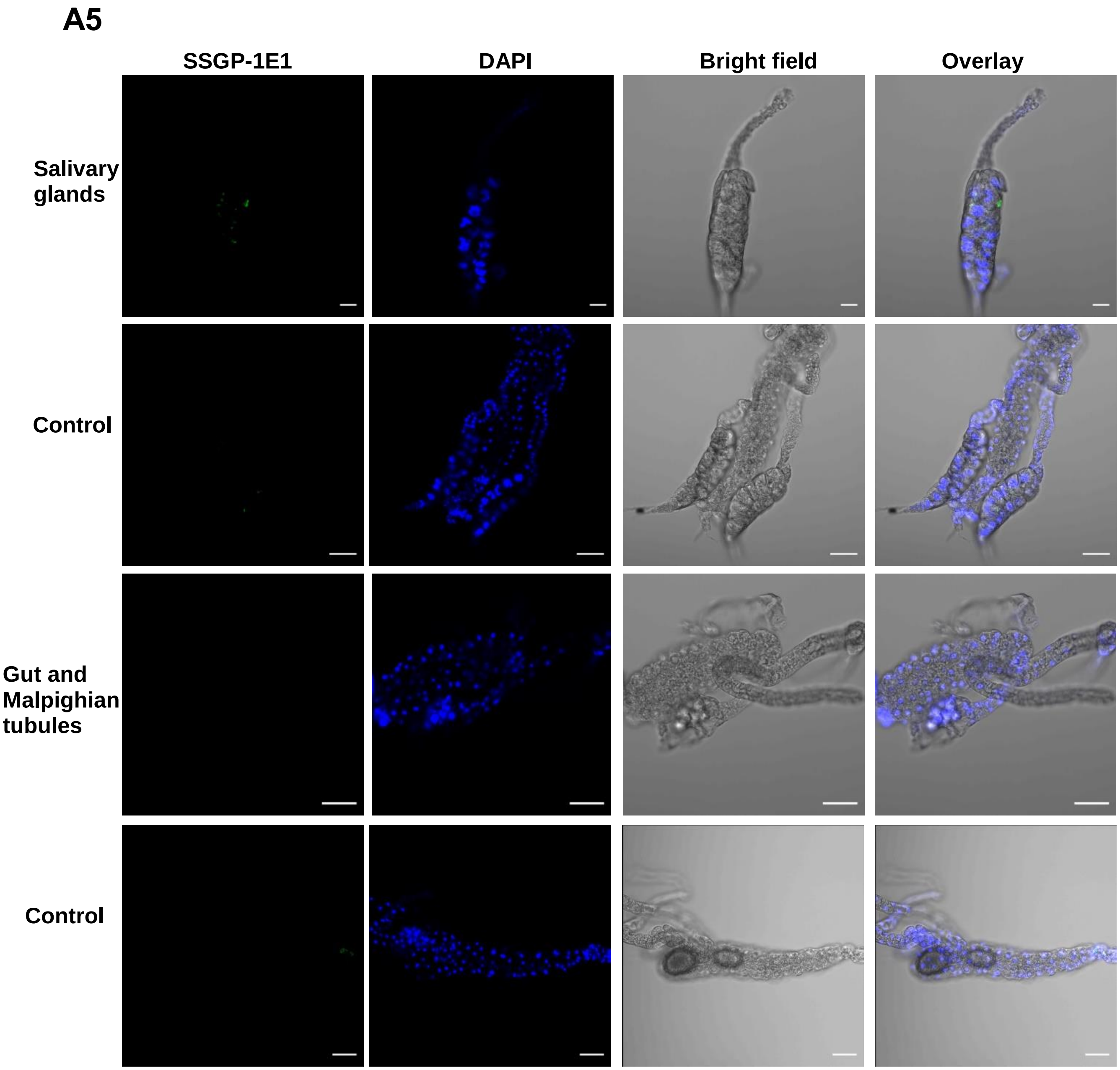
A5
SSGP-1E1
DAPI
Bright field
Overlay
Salivary glands
Control
Gut and Malpighian tubules
Control

S3,2: B1 to B5 are tissues staining from **two days** old larvae with five different antibodies and their respective controls. Denotations are the same as S3,1.

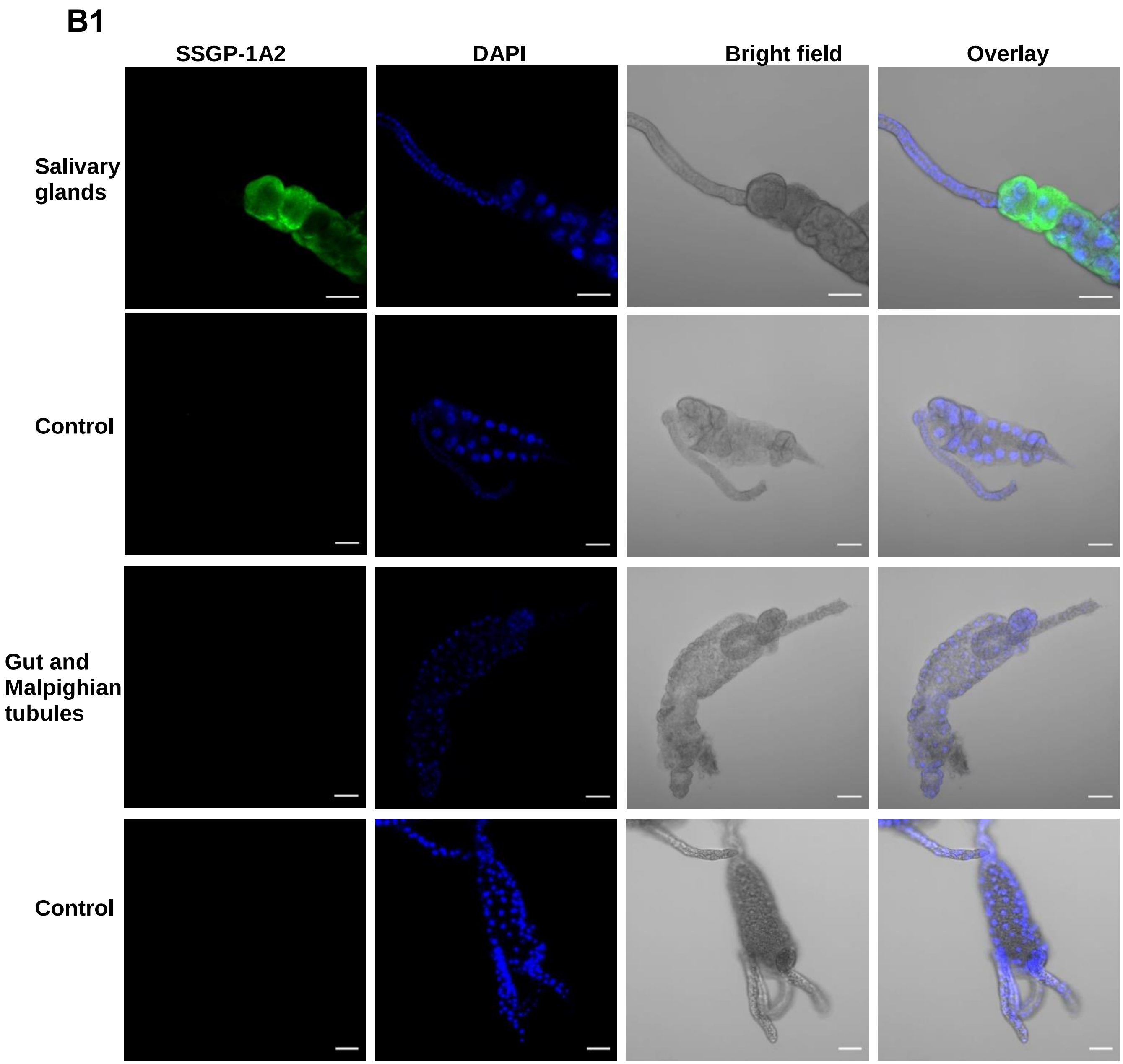

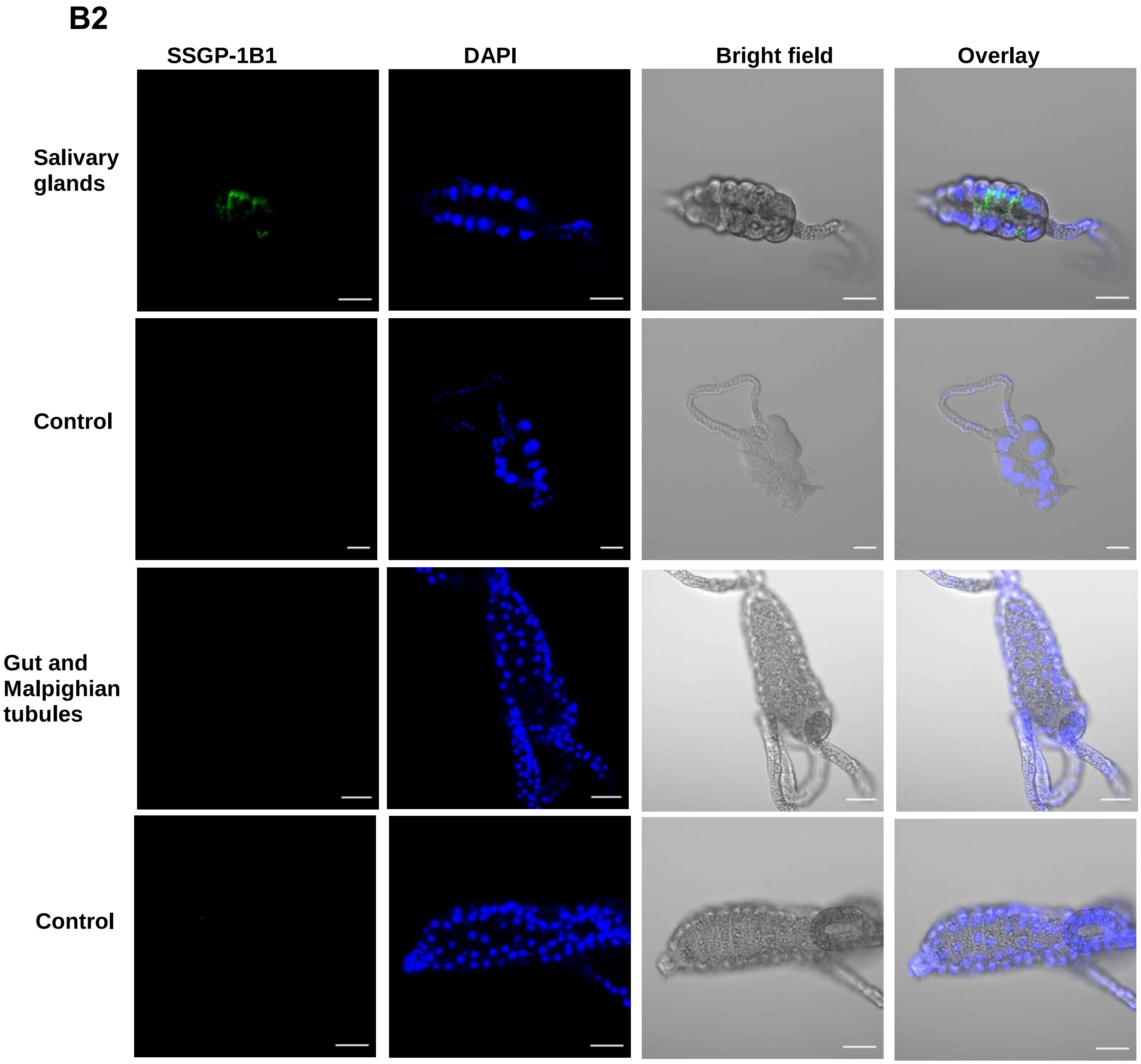
B2
SSGP-1B1
DAPI
Bright field
Overlay
Salivary glands
Control
Gut and Malpighian tubules
Control

B3

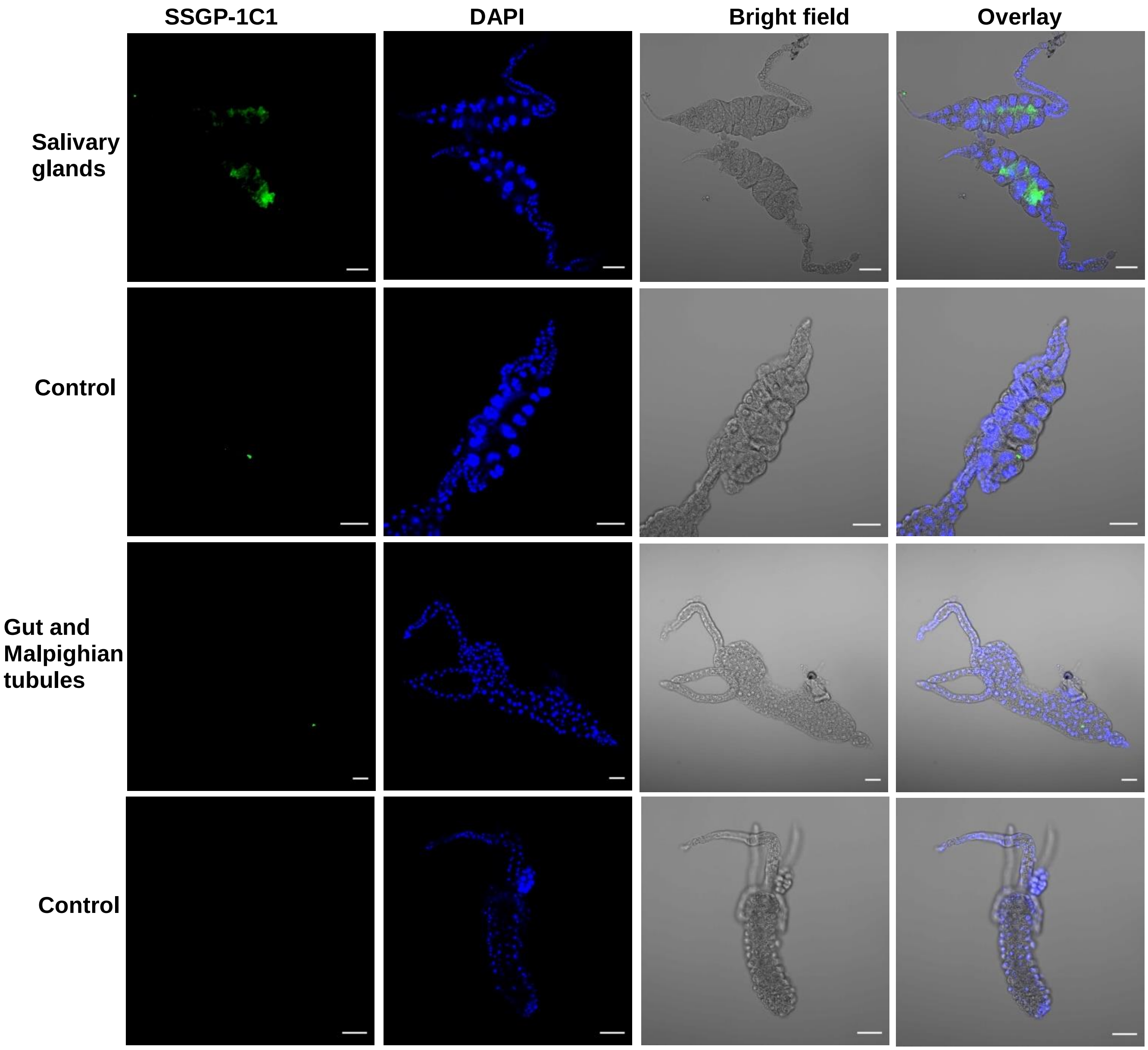
SSGP-1C1
DAPI
Bright field
Overlay
Salivary glands
Control
Gut and Malpighian tubules
Control

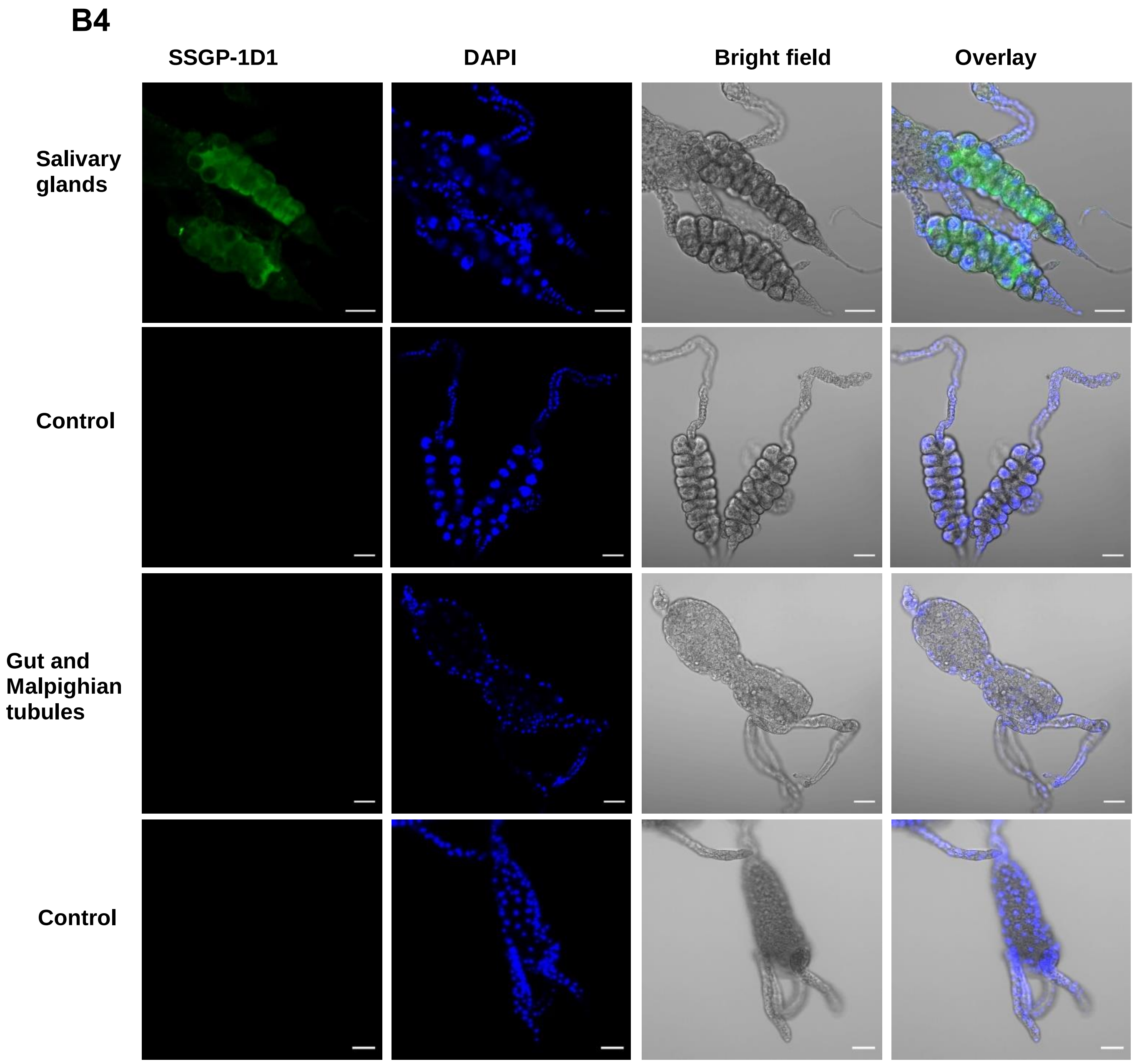
B4
SSGP-1D1
DAPI
Bright field
Overlay
Salivary glands
Control
Gut and Malpighian tubules
Control

B5

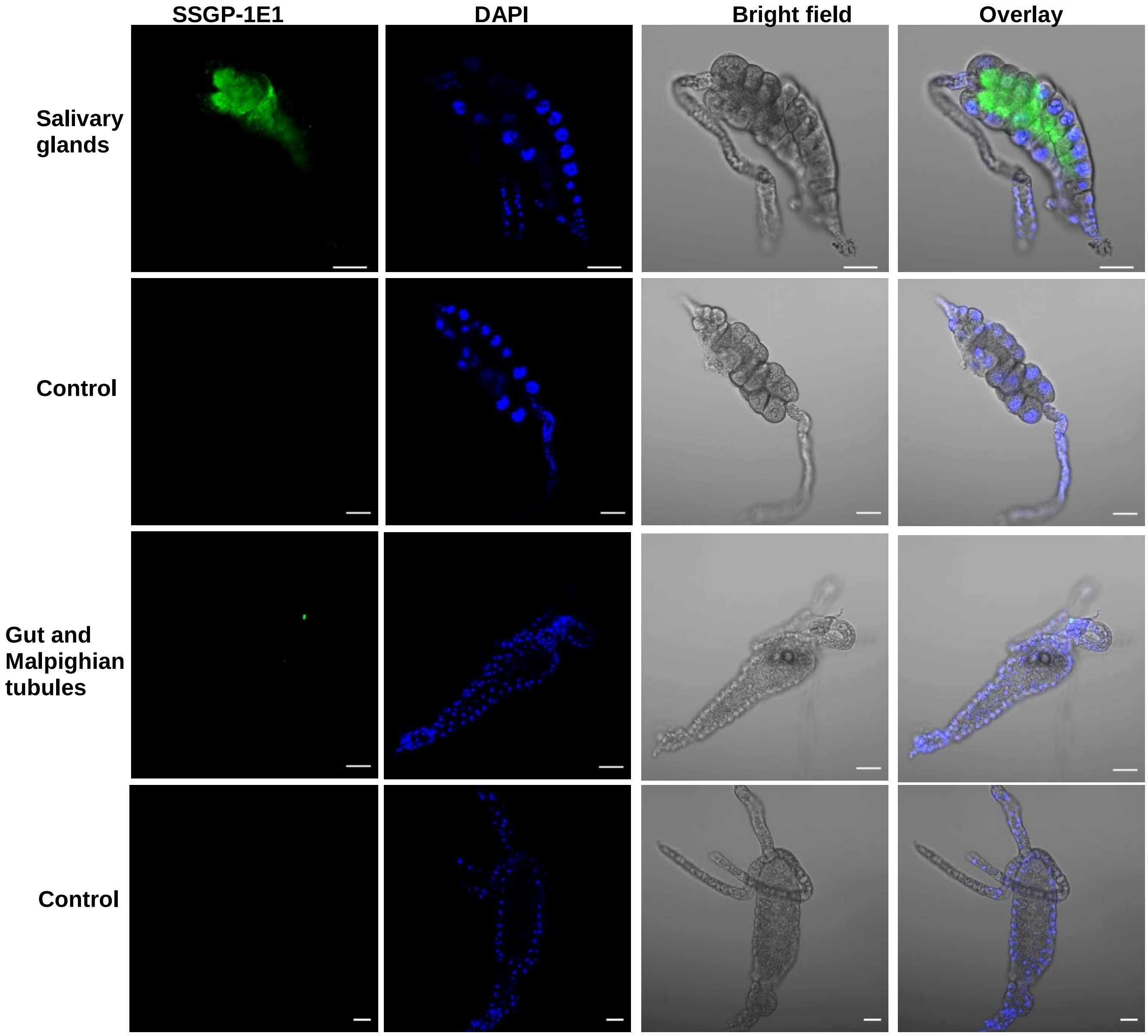
SSGP-1E1
DAPI
Bright field
Overlay
Salivary glands
Control
Gut and Malpighian tubules
Control

S3,3: C1 to C5 are tissues staining from **three days** old larvae with five different antibodies and their respective controls. Denotations are the same as S3,1. Red circle points to the signal of SSGP-1E1 in a few cells in the foregut.

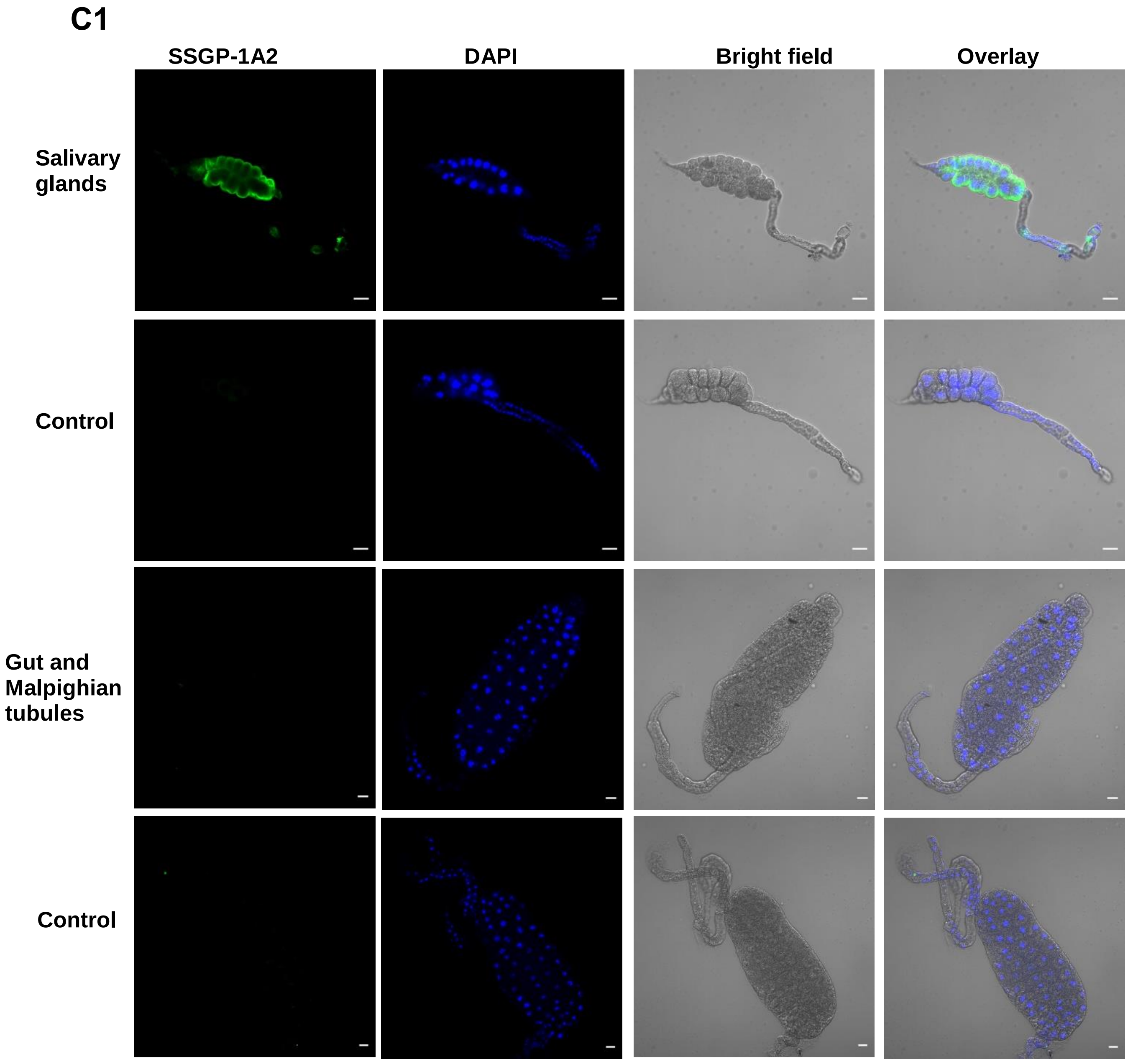

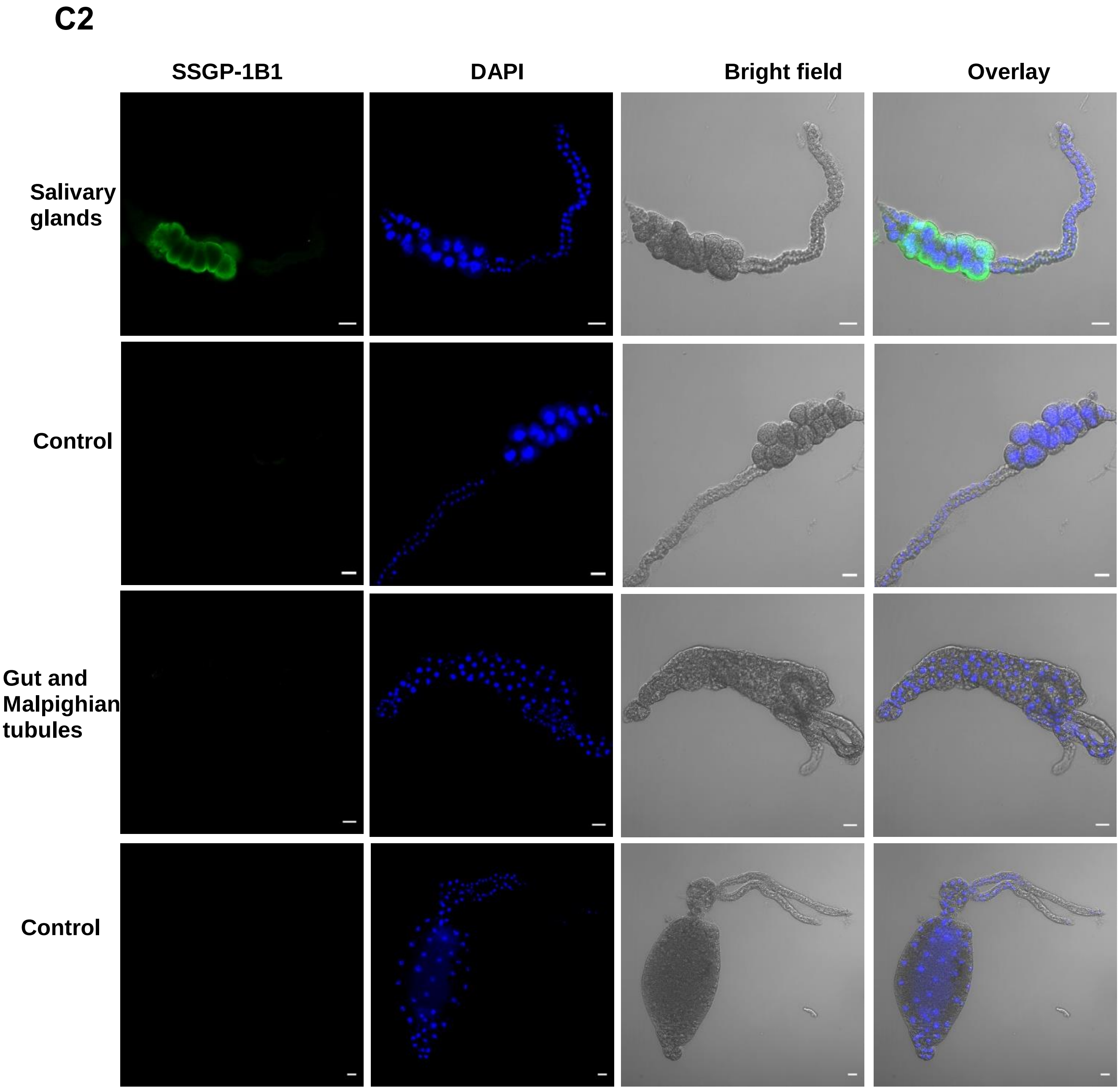
C2
SSGP-1B1
DAPI
Bright field
Overlay
Salivary glands
Control
Gut and Malpighian tubules
Control

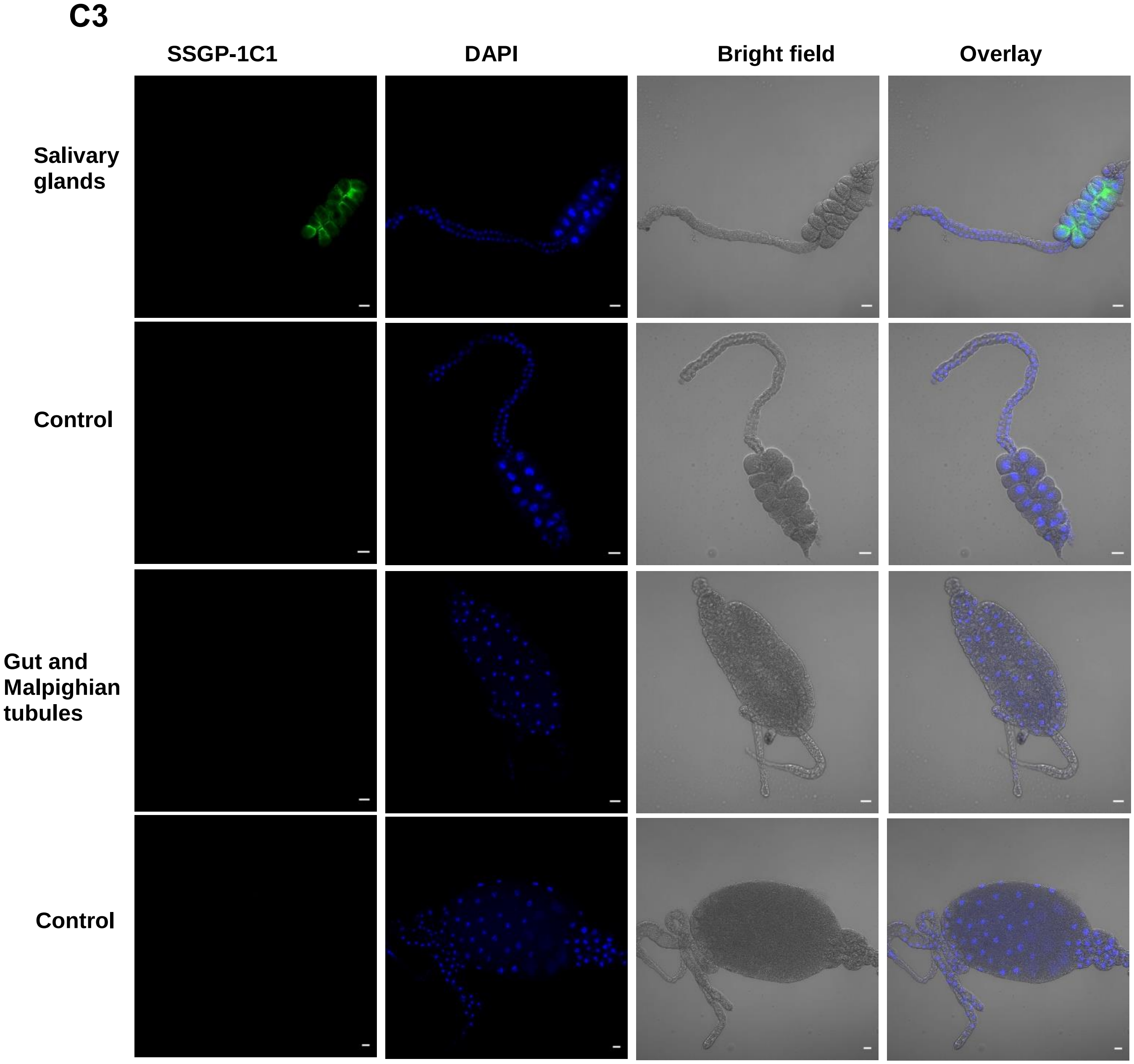
C3
SSGP-1C1
DAPI
Bright field
Overlay
Salivary glands
Control
Gut and Malpighian tubules
Control

C4

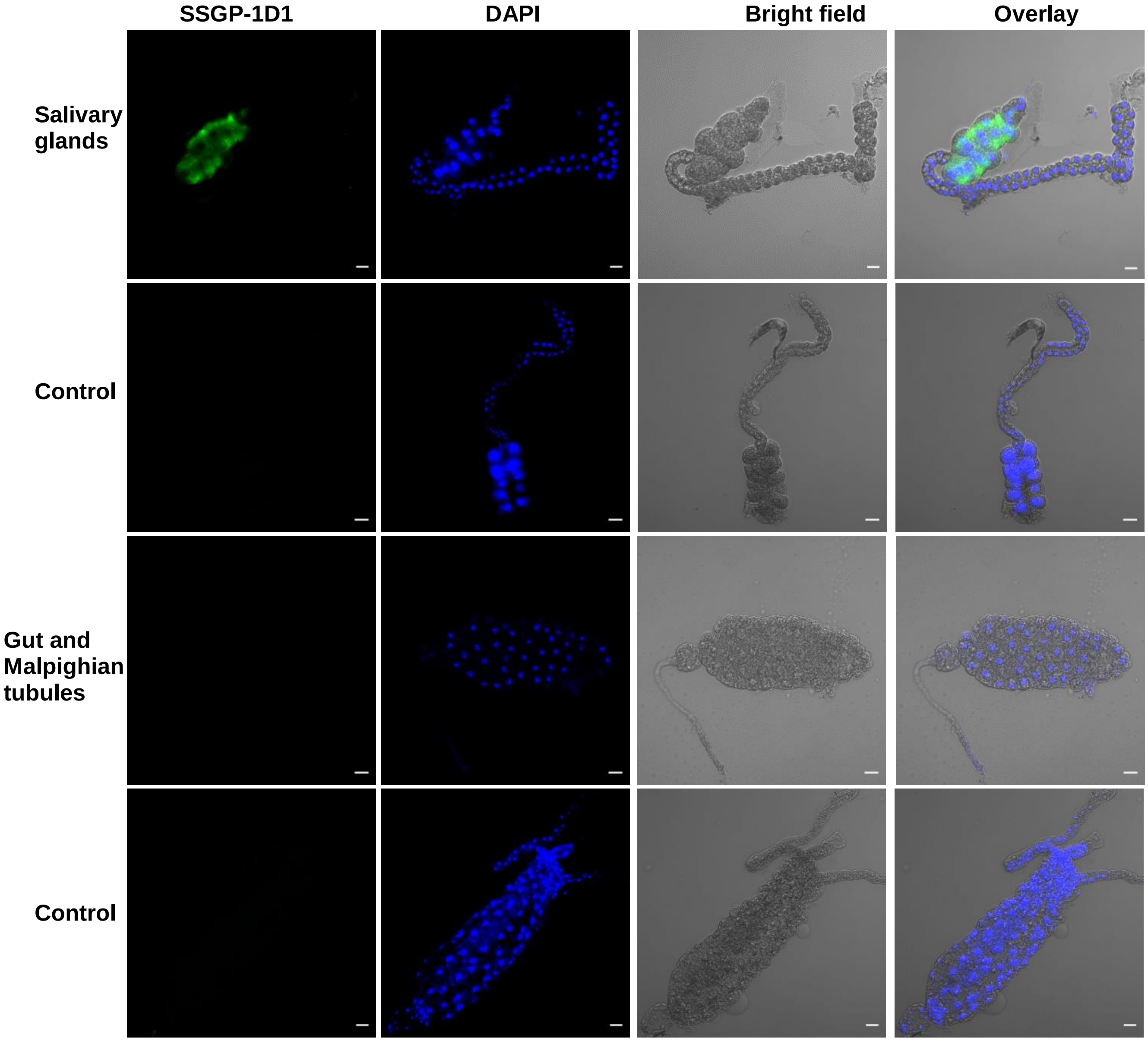
SSGP-1D1
DAPI
Bright field
Overlay
Salivary glands
Control
Gut and Malpighian tubules
Control

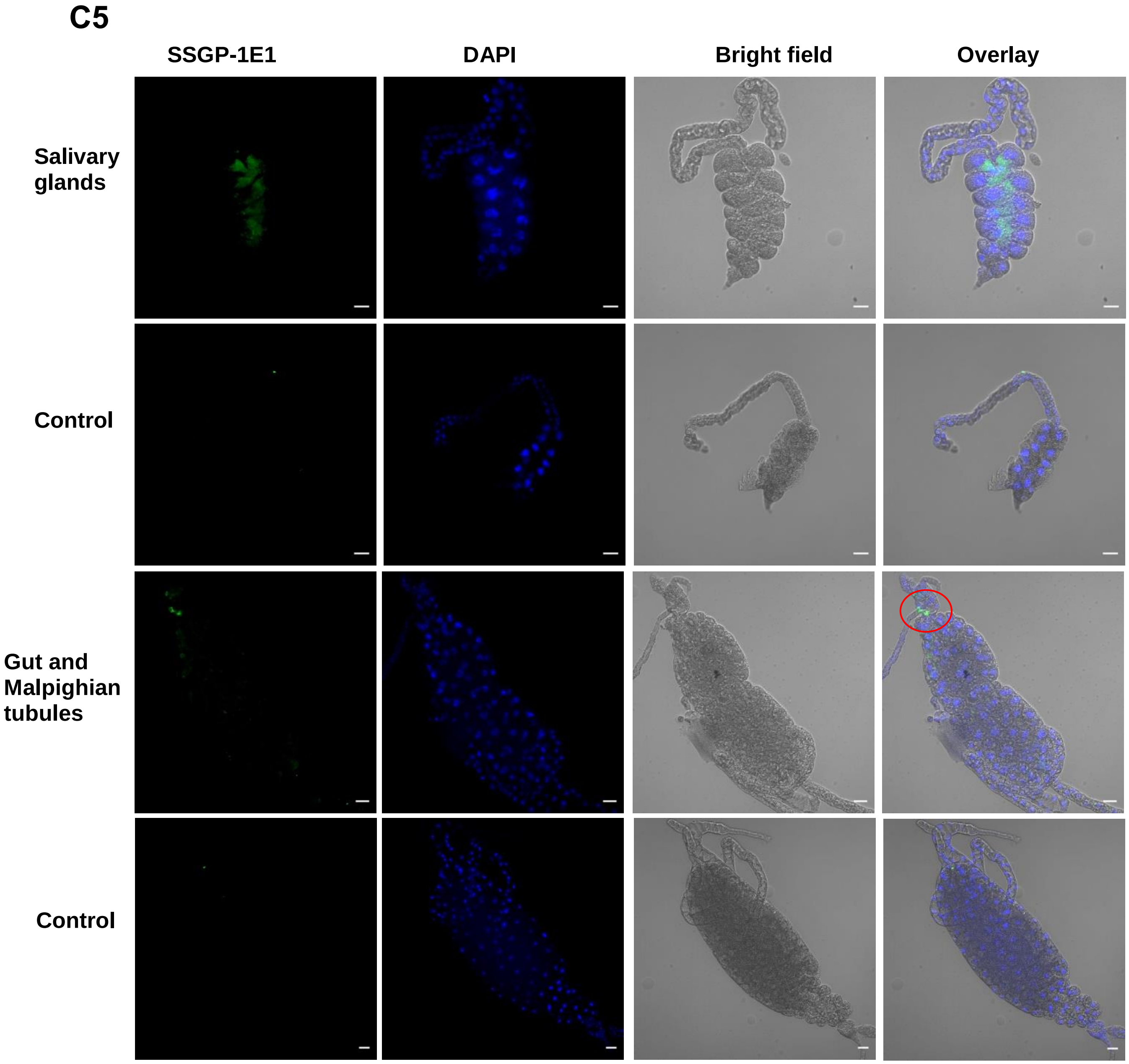
C5
SSGP-1E1
DAPI
Bright field
Overlay
Salivary glands
Control
Gut and Malpighian tubules
Control

S3,4: D1 to D5 are tissues staining from **six days** old larvae with five different antibodies and their respective controls. Denotations are the same as S3,1. Red circle points to the signal of SSGP-1E1 in a few cells in the foregut.

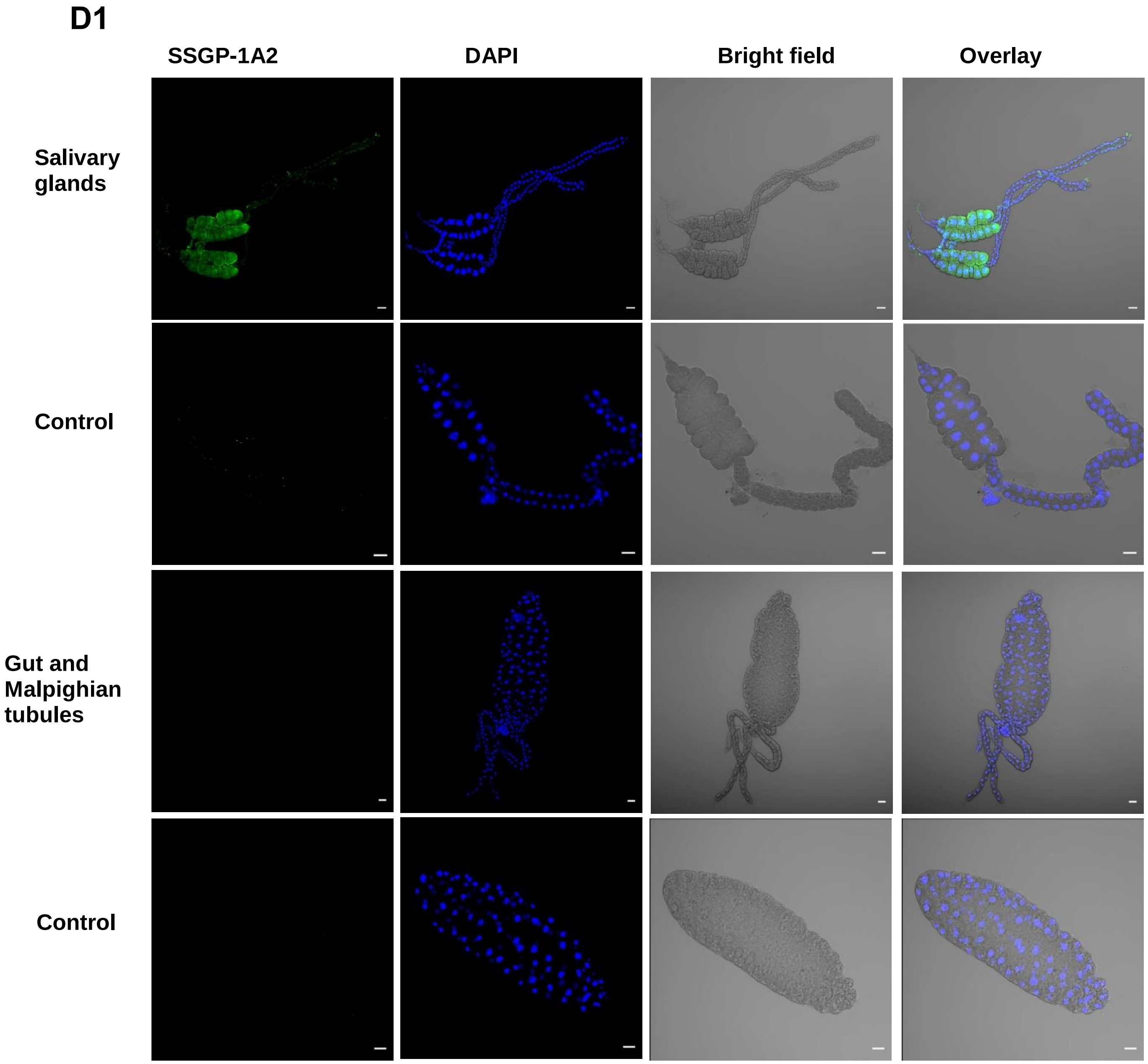

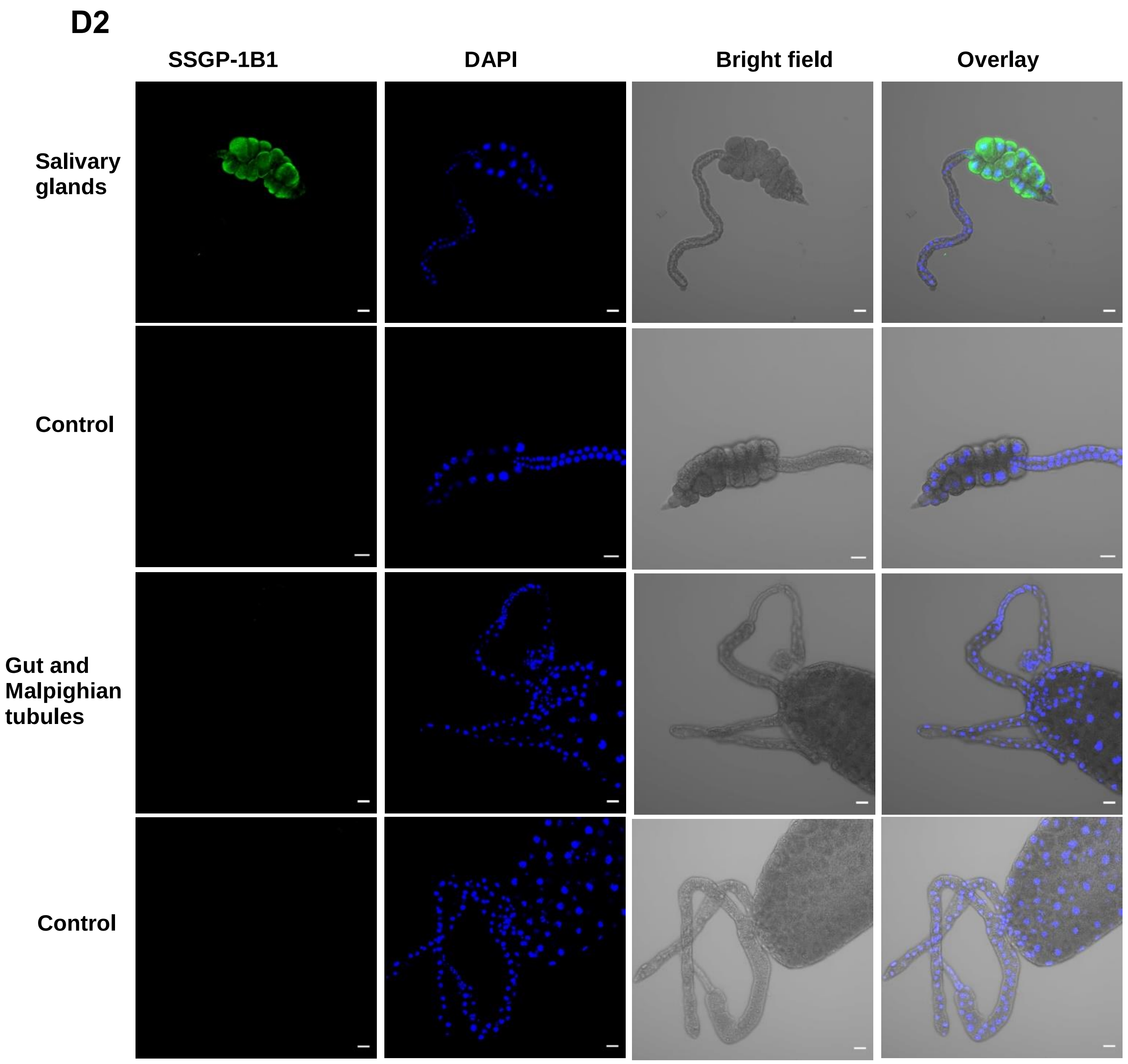
D2
SSGP-1B1
DAPI
Bright field
Overlay
Salivary glands
Control
Gut and Malpighian tubules
Control

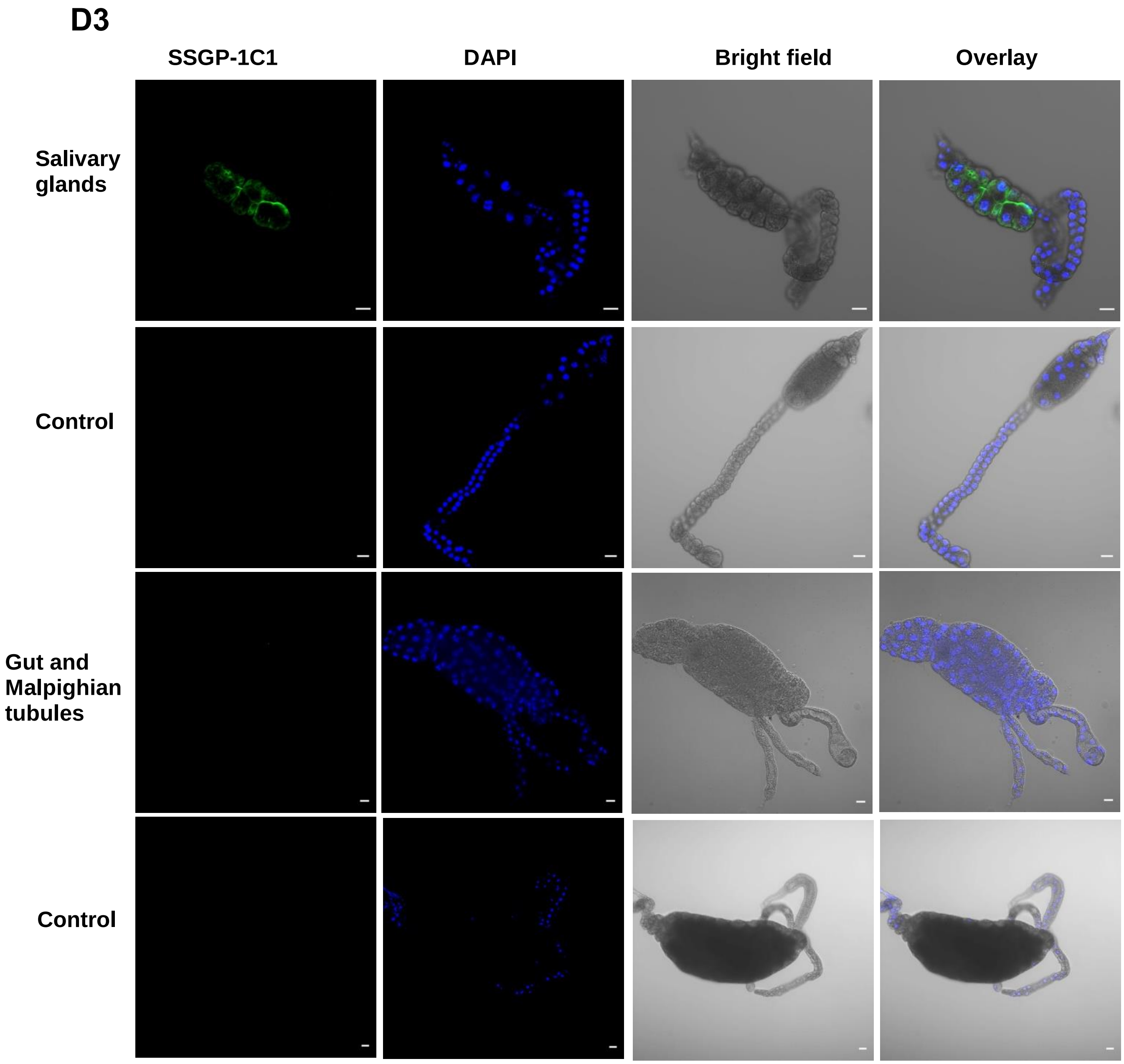
D3
SSGP-1C1
DAPI
Bright field
Overlay
Salivary glands
Control
Gut and Malpighian tubules
Control

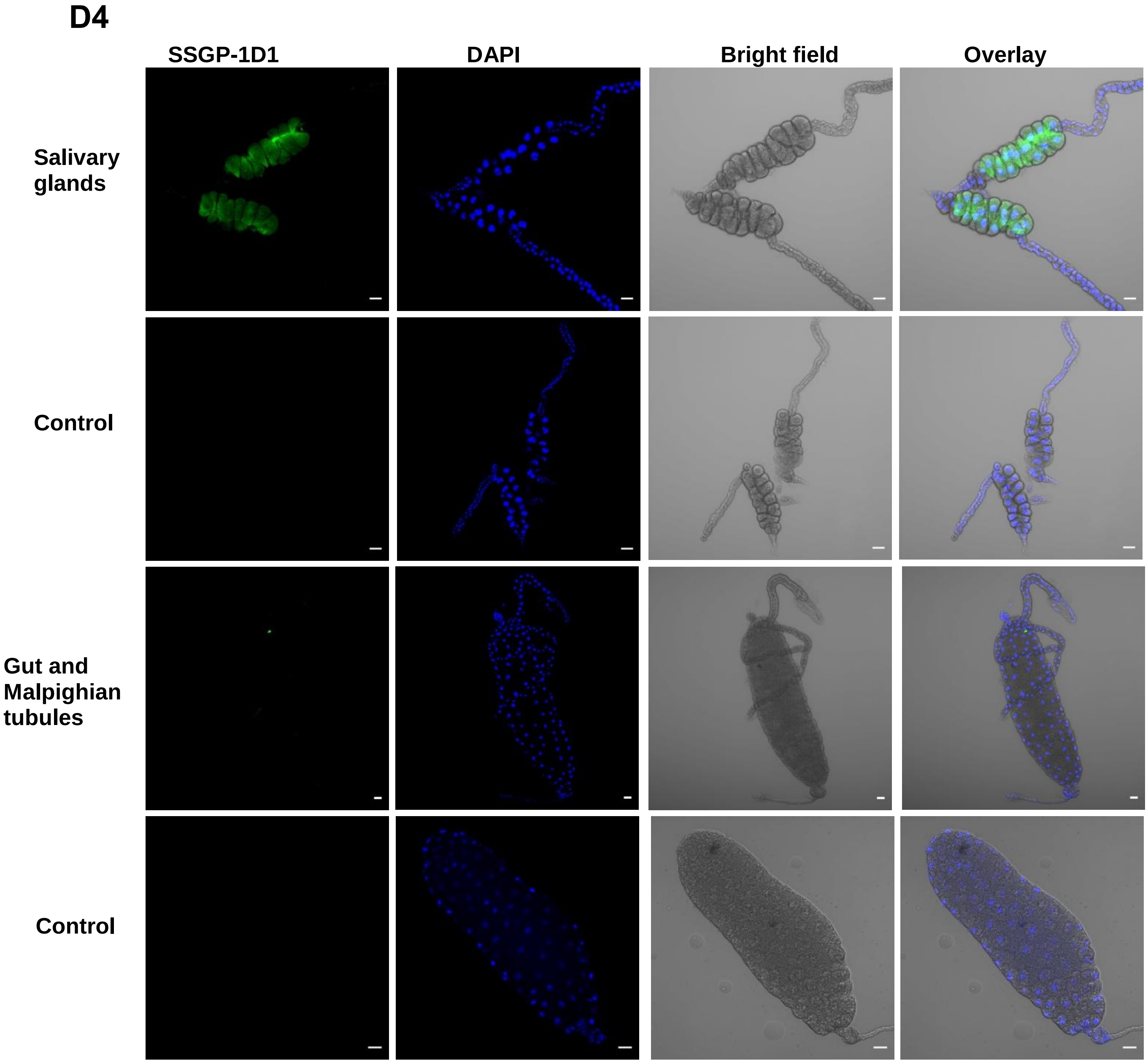
D4
SSGP-1D1
DAPI
Bright field
Overlay
Salivary glands
Control
Gut and Malpighian tubules
Control

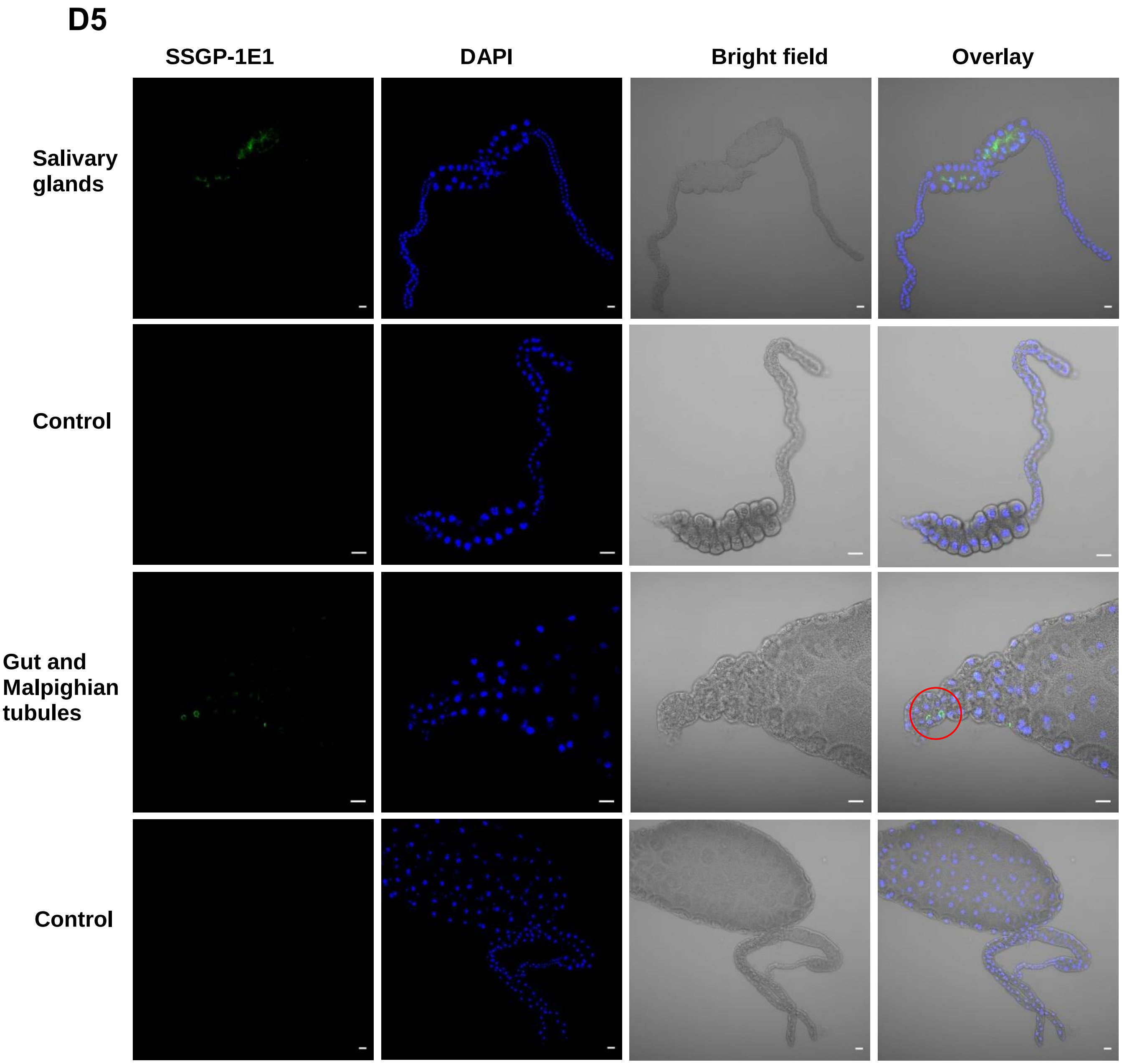
D5
SSGP-1E1
DAPI
Bright field
Overlay
Salivary glands
Control
Gut and Malpighian tubules
Control

S3,5: E1 to E5 are tissues staining from **ten days** old larvae with five different antibodies and their respective controls. Denotations are the same as S3,1.

E1

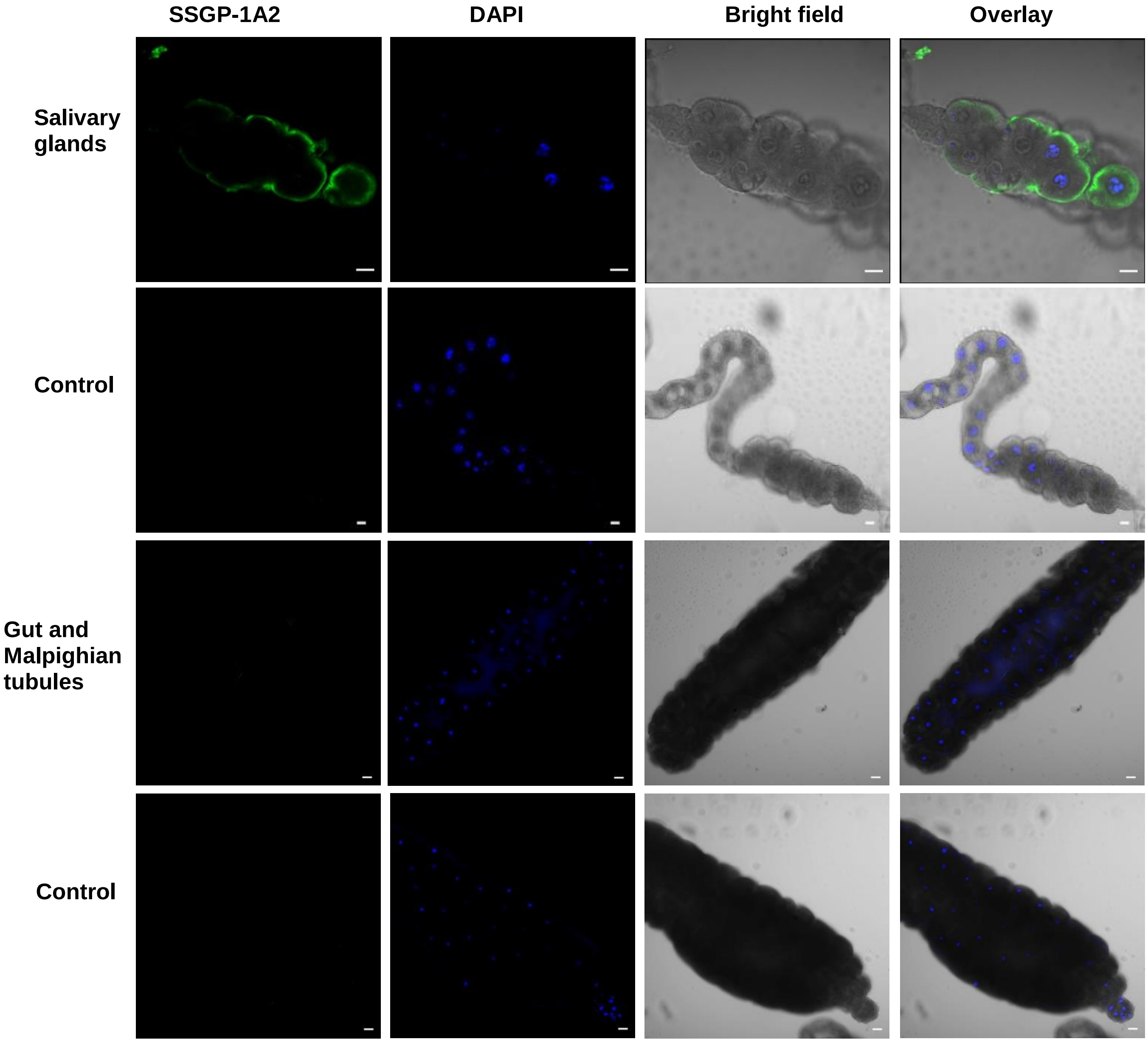

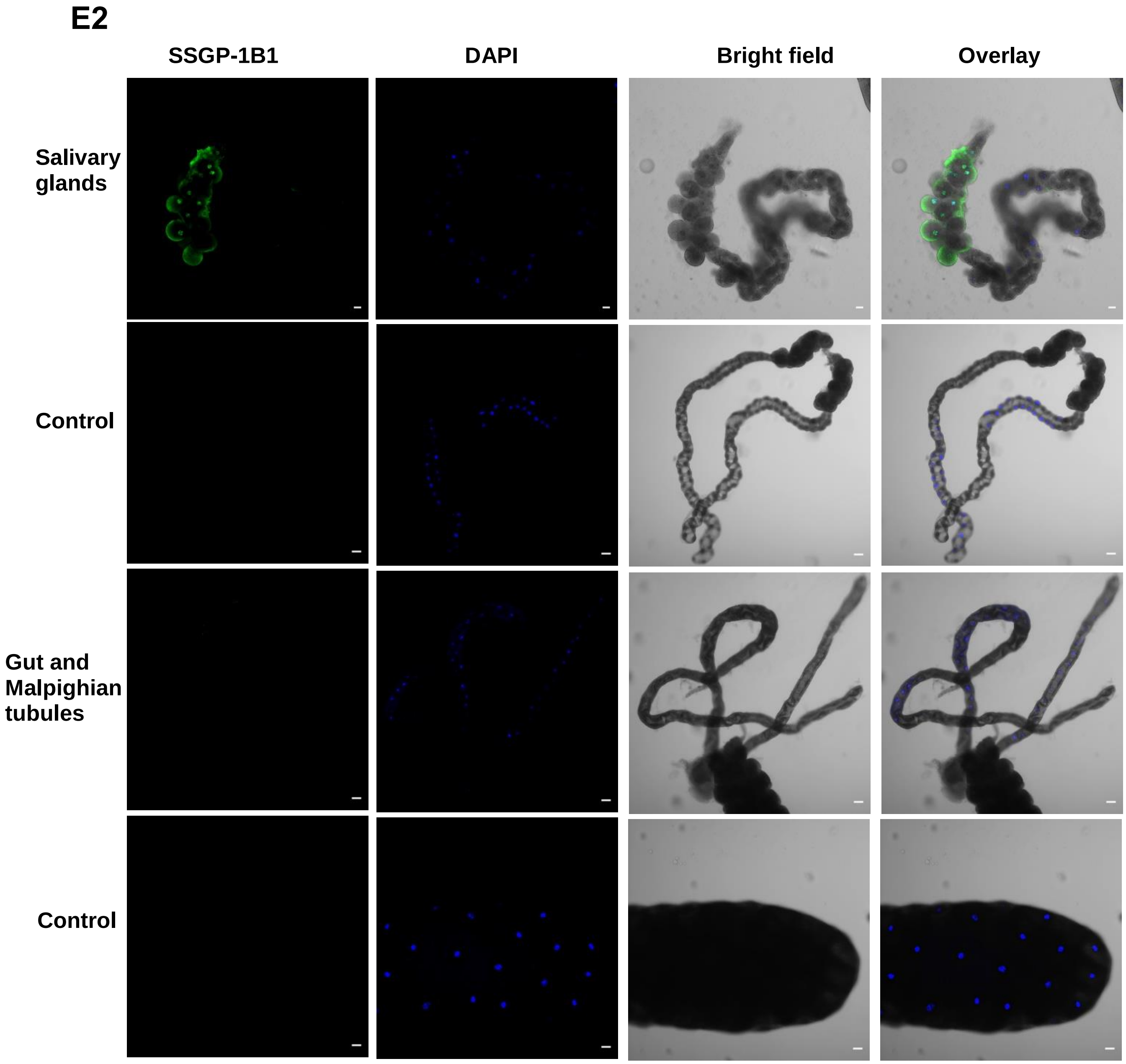

E2
SSGP-1B1
DAPI
Bright field
Overlay
Salivary glands
Control
Gut and Malpighian tubules
Control

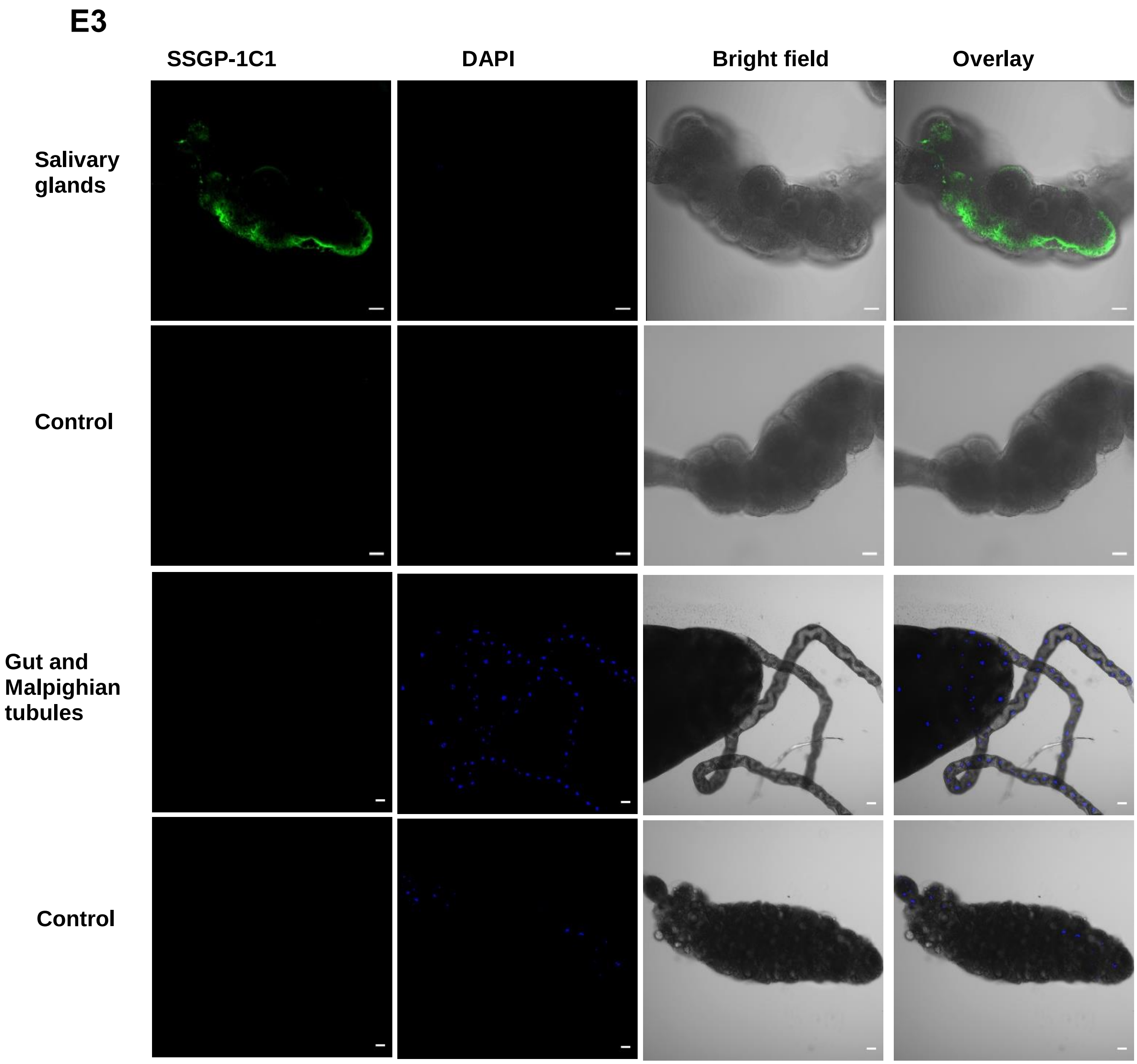
E3
SSGP-1C1
DAPI
Bright field
Overlay
Salivary glands
Control
Gut and Malpighian tubules
Control

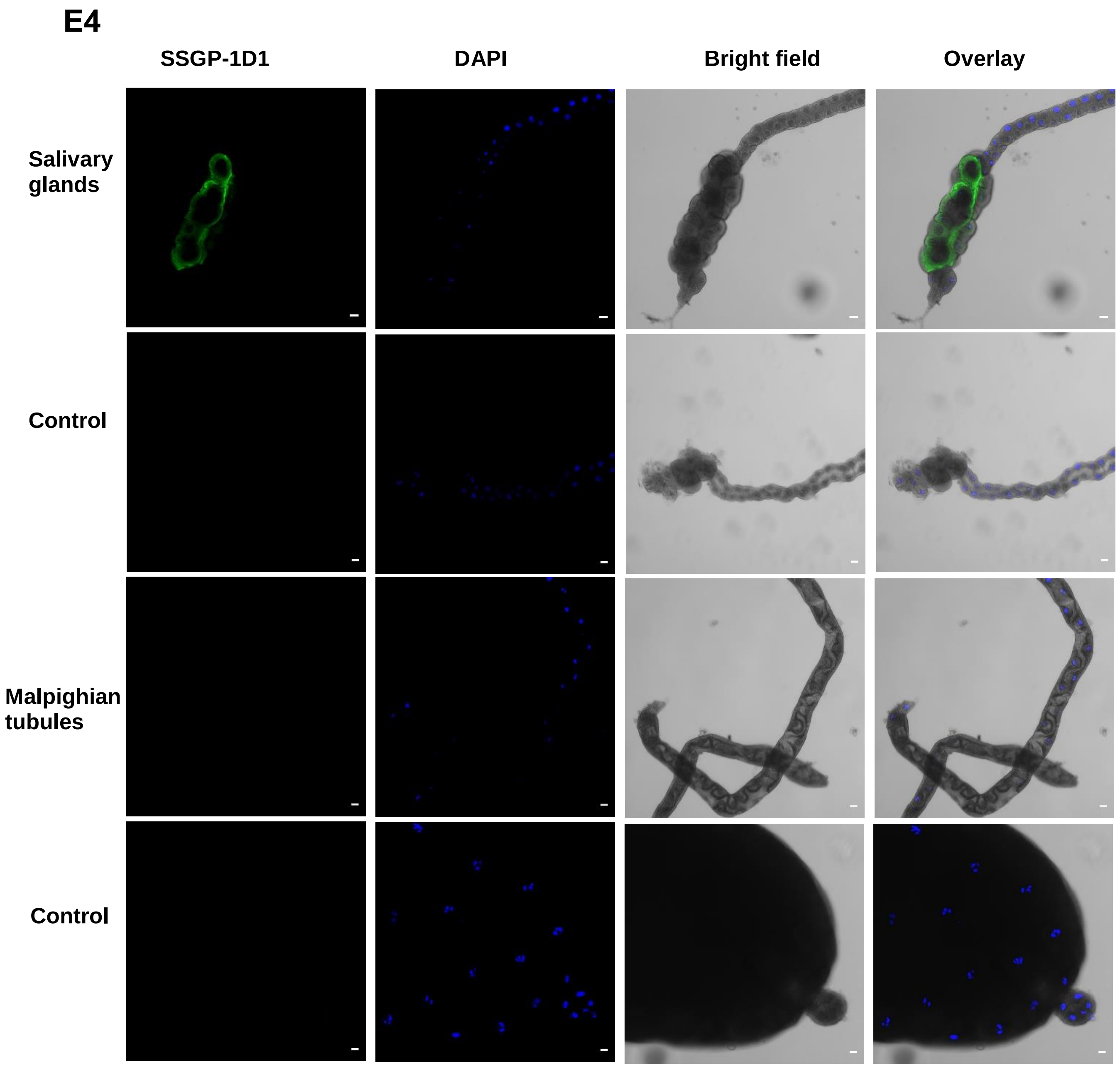

E4
SSGP-1D1
DAPI
Bright field
Overlay
Salivary glands
Control
Malpighian tubules
Control

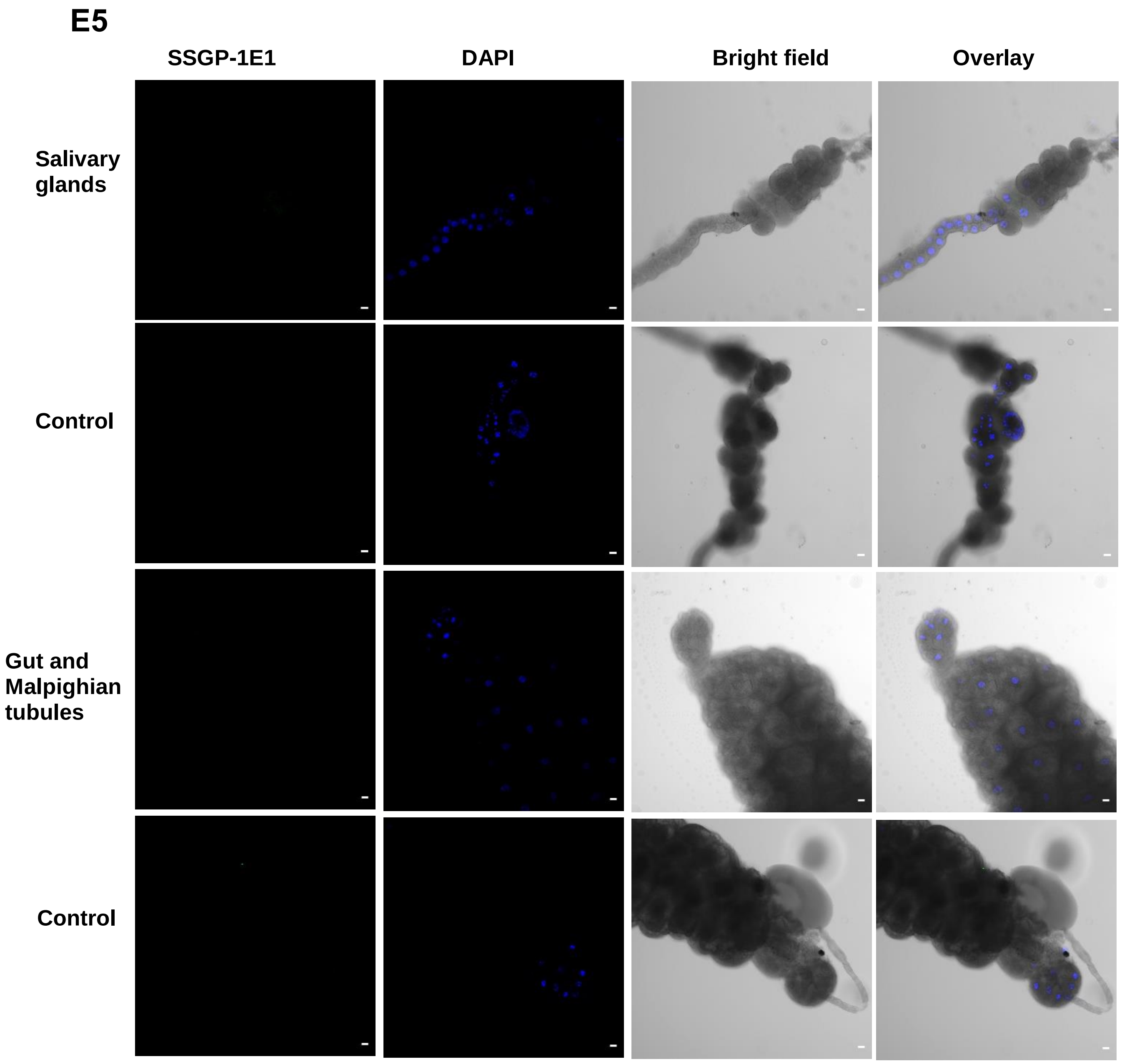
E5
SSGP-1E1
DAPI
Bright field
Overlay
Salivary glands
Control
Gut and Malpighian tubules
Control

Figure S4. Localization of Family-1 effectors in susceptible and resistant wheat tissues after three days of larvae feeding detected by immunostaining. Samples of wheat seedling with 2-3 mm length were cut at the feeding site, longitude paraffin molds were sectioned and stained with each of the five antibodies separately. Corresponding negative controls of non-infested susceptible and resistant wheat tissues are presented with each of the five antibodies. Magenta arrowheads are pointing to signal derived from the respective effector. A yellow arrowhead is pointing to the location of Hessian fly larva. White arrowheads point to dead cells. Bars, 20µm.

S4,1: Localization of Family-1 effectors in tissues from the susceptible cultivar 'Newton'. A1 to A4 indicate staining of four antibodies with their corresponding controls.

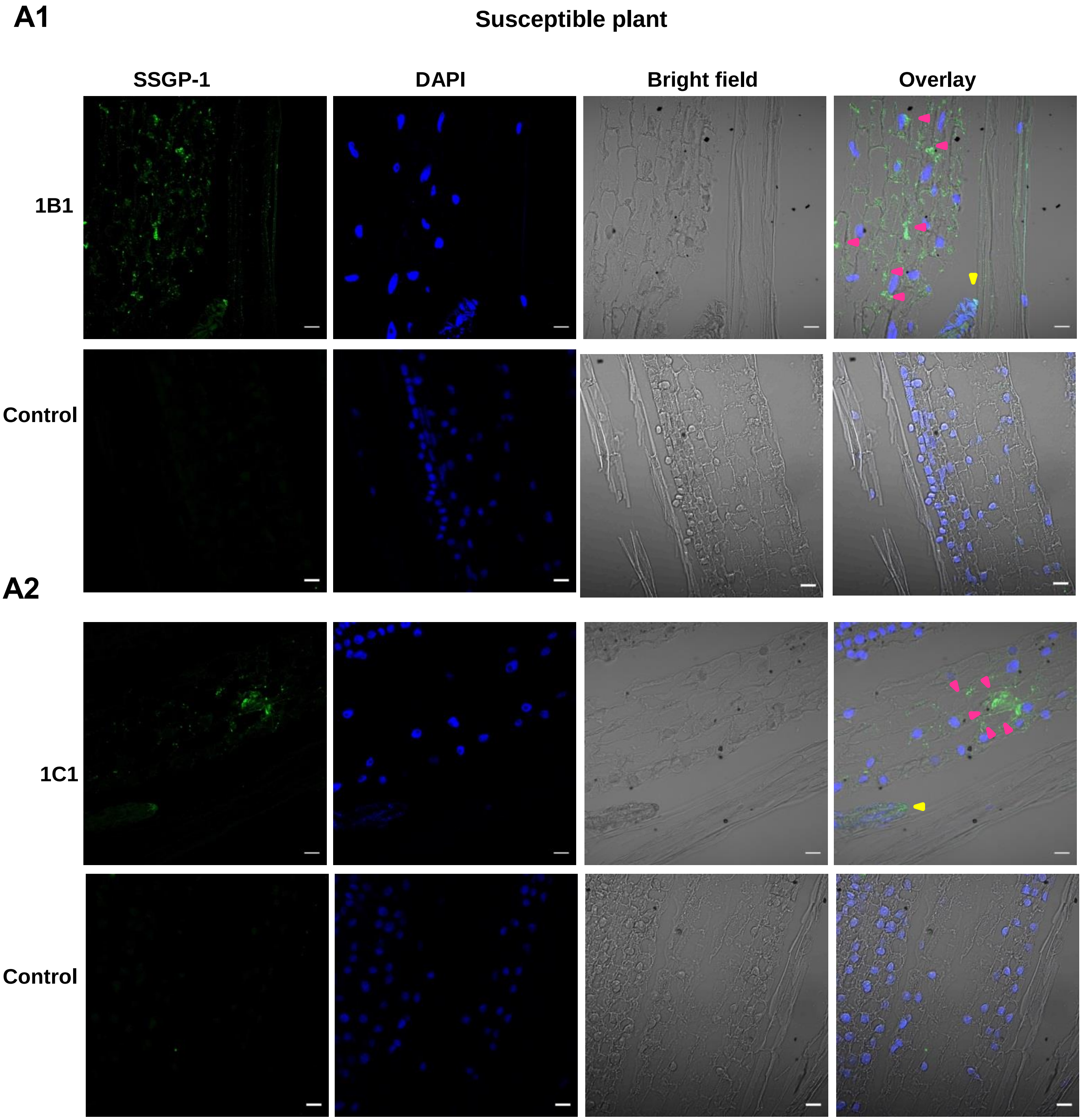

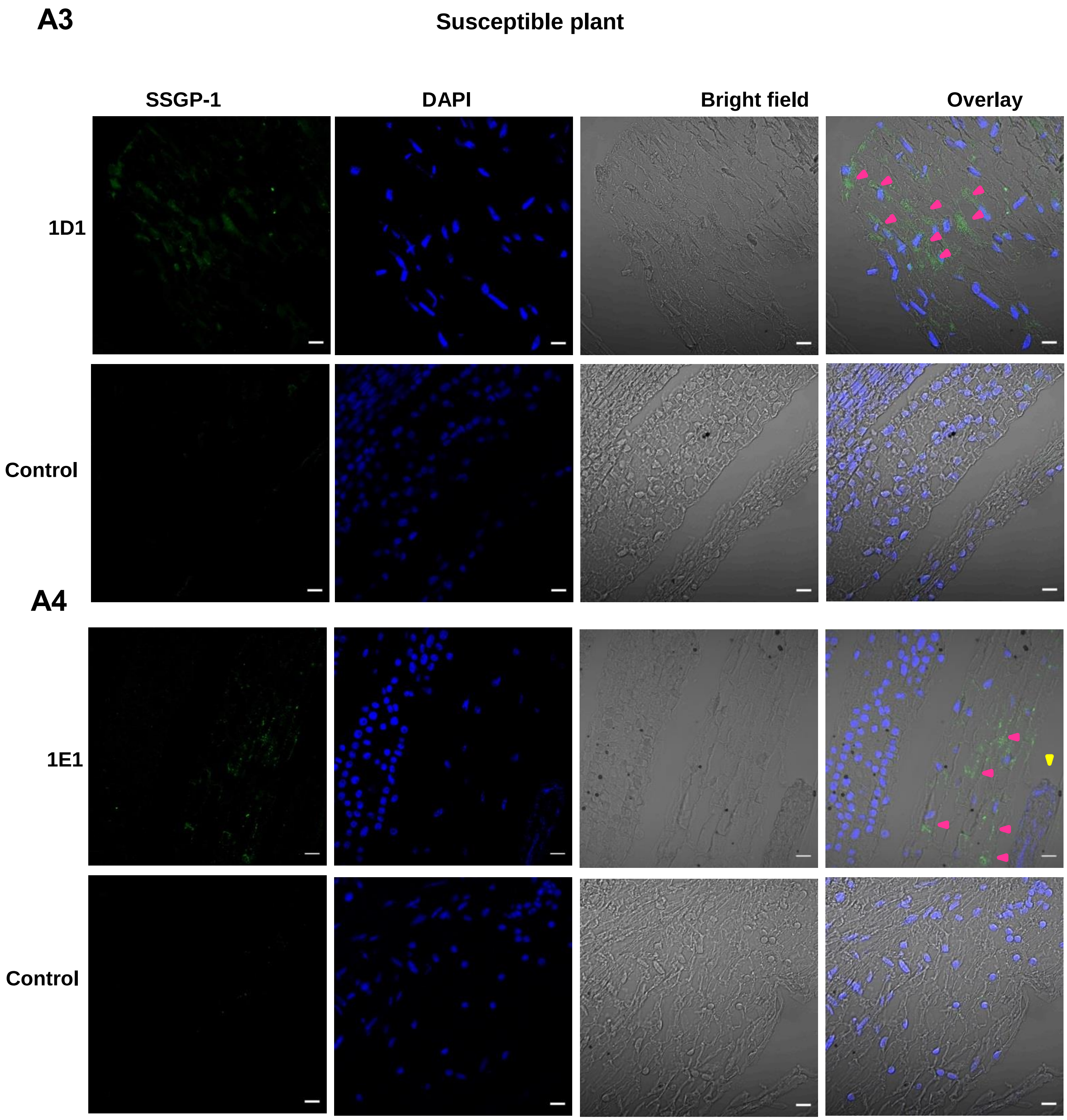
A3
Susceptible plant
SSGP-1
DAPI
Bright field
Overlay
1D1
Control
A4
1E1
Control

S4,2: Localization of Family-1 effectors in tissues from the resistant cultivar 'Molly' *H13*. B1 to B4 indicate staining with four antibodies with their corresponding controls.

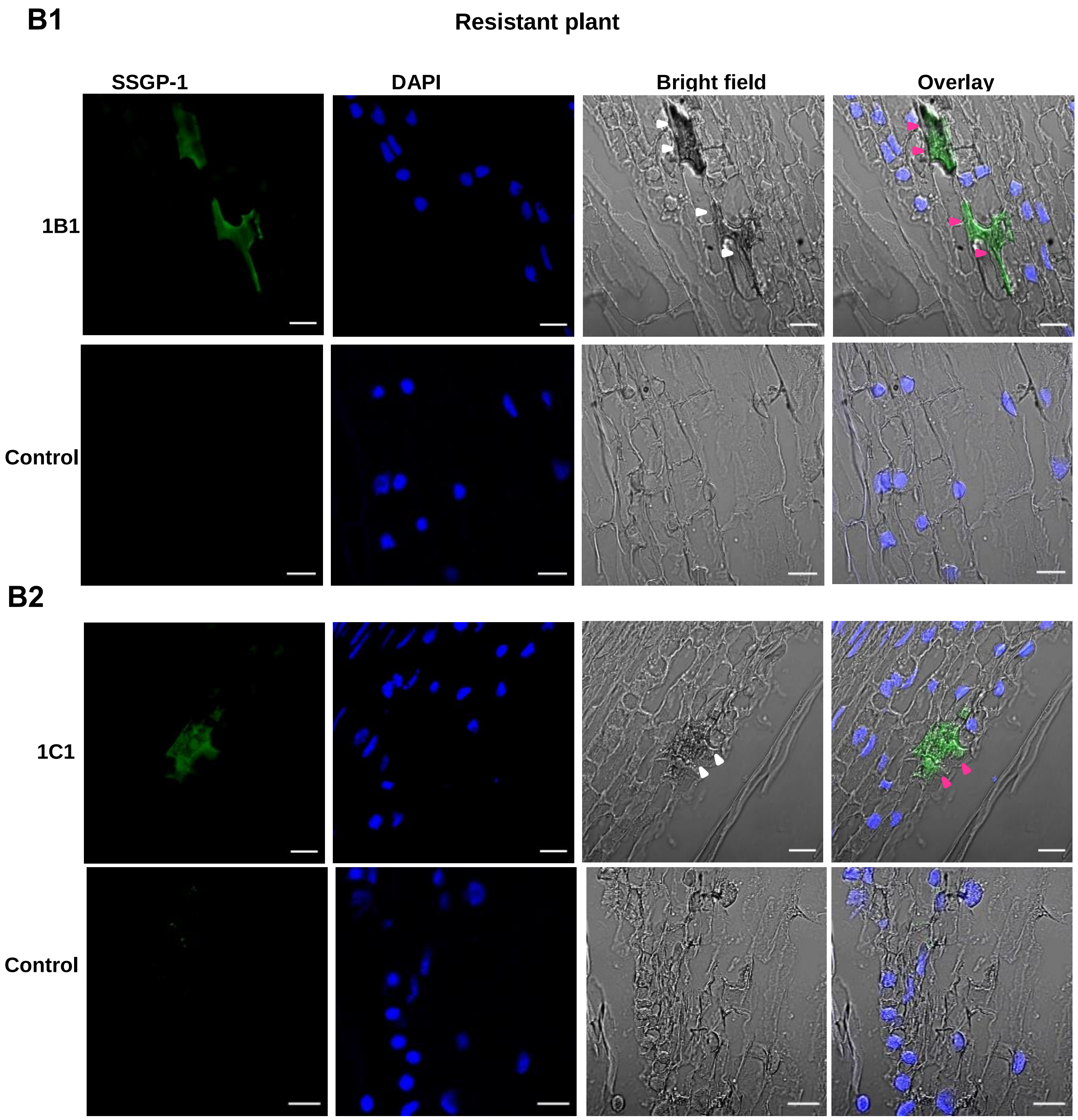

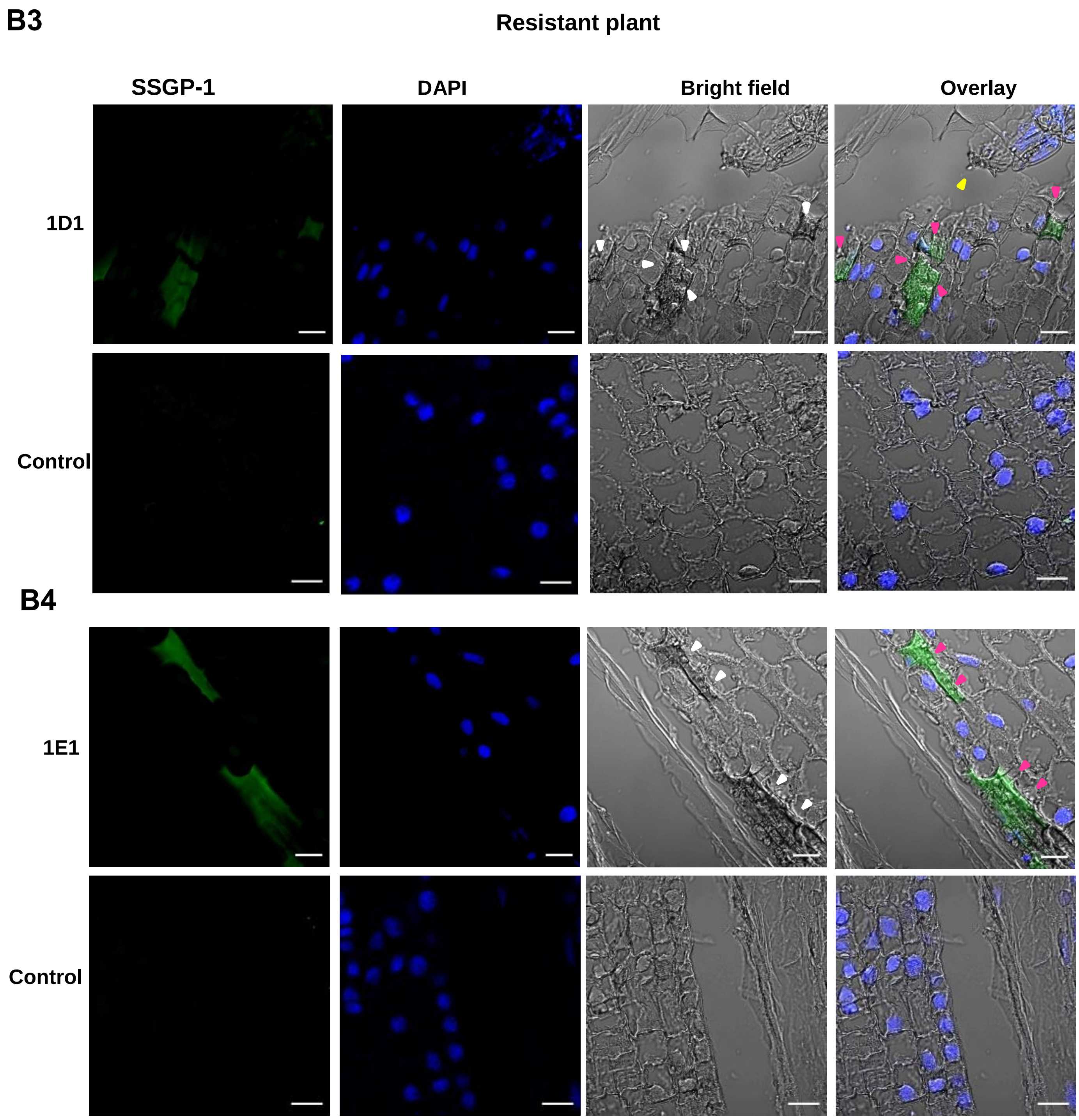
B3
Resistant plant
SSGP-1
DAPI
Bright field
Overlay
1D1
Control
B4
1E1
Control

S4,3: Localization of Family-1 effectors in tissues from the resistant cultivar 'H6'. C1 to C5 indicate staining with each of the five antibodies with their corresponding controls.

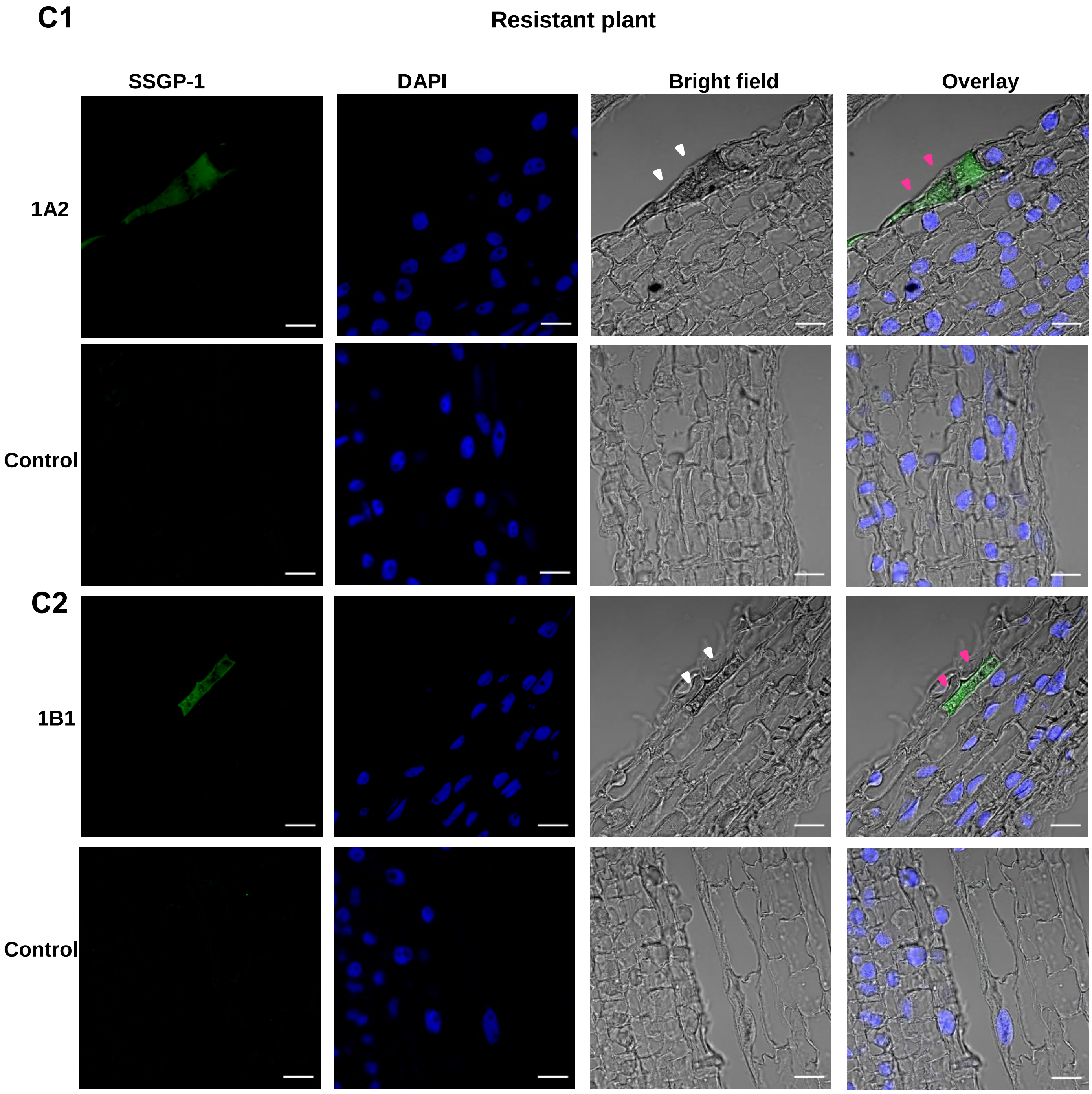

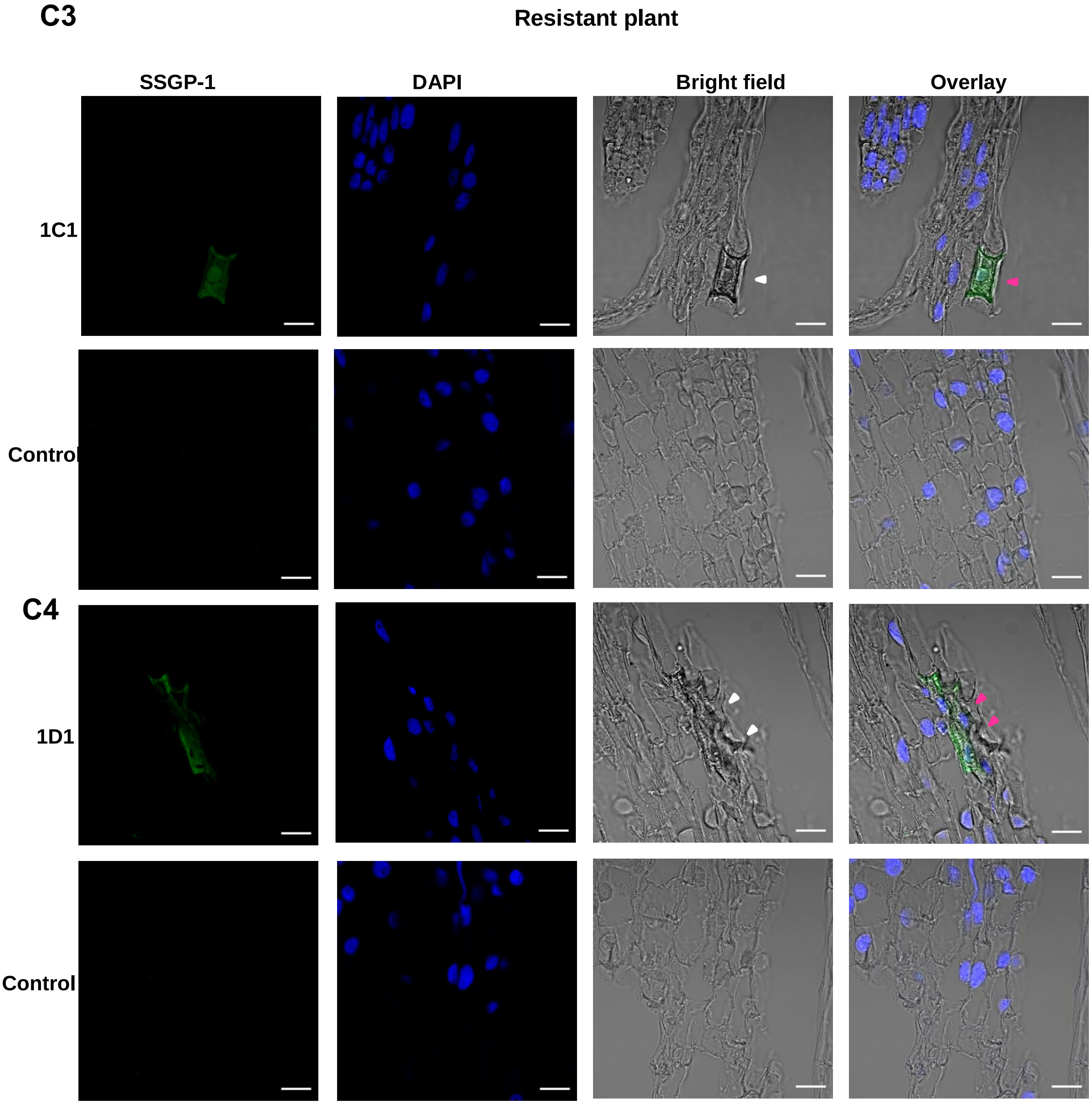
C3
Resistant plant
SSGP-1
DAPI
Bright field
Overlay
1C1
Control
C4
1D1
Control

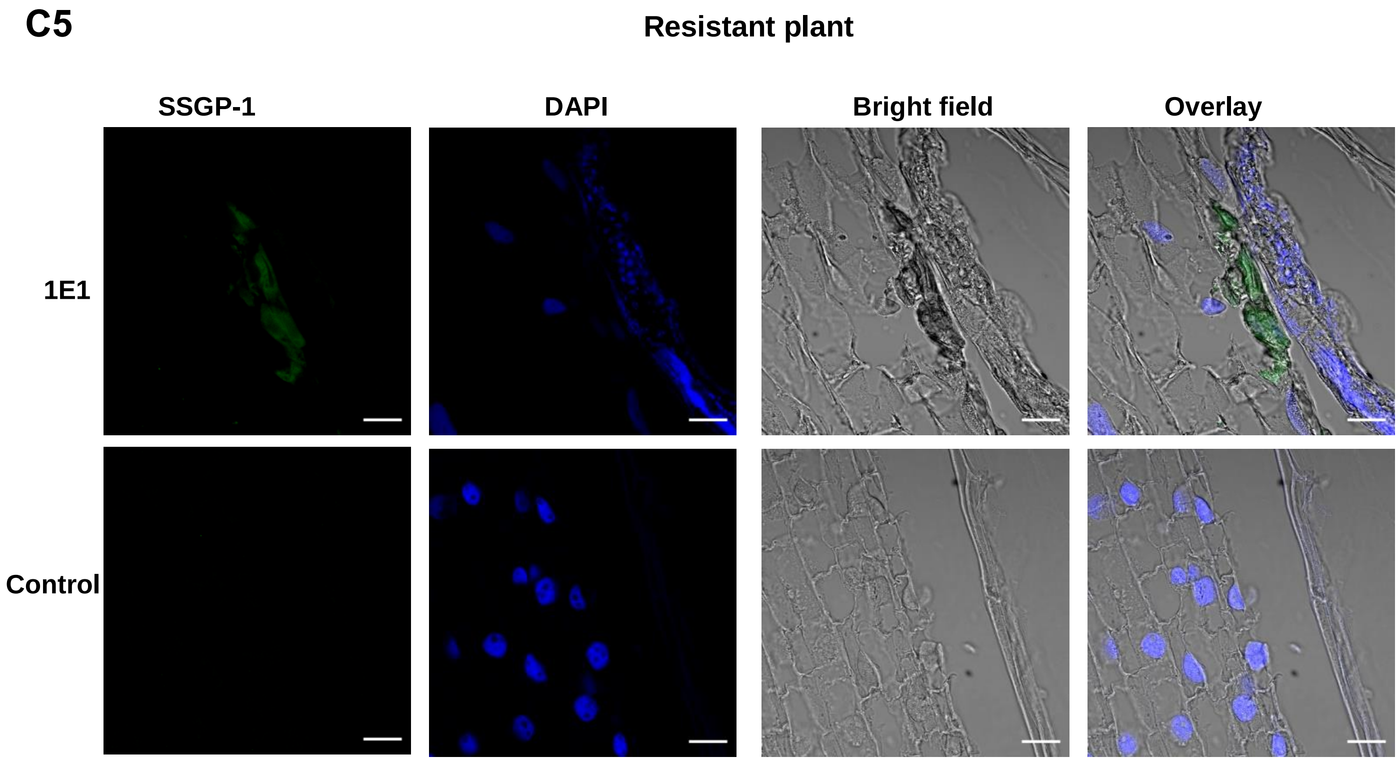
C5
Resistant plant
SSGP-1
DAPI
Bright field
Overlay
1E1
Control

S4,4: Localization of Family-1 effectors in tissues from the resistant cultivar ‘H21’. D1 to D5 indicate staining with each of the five antibodies with their corresponding controls.

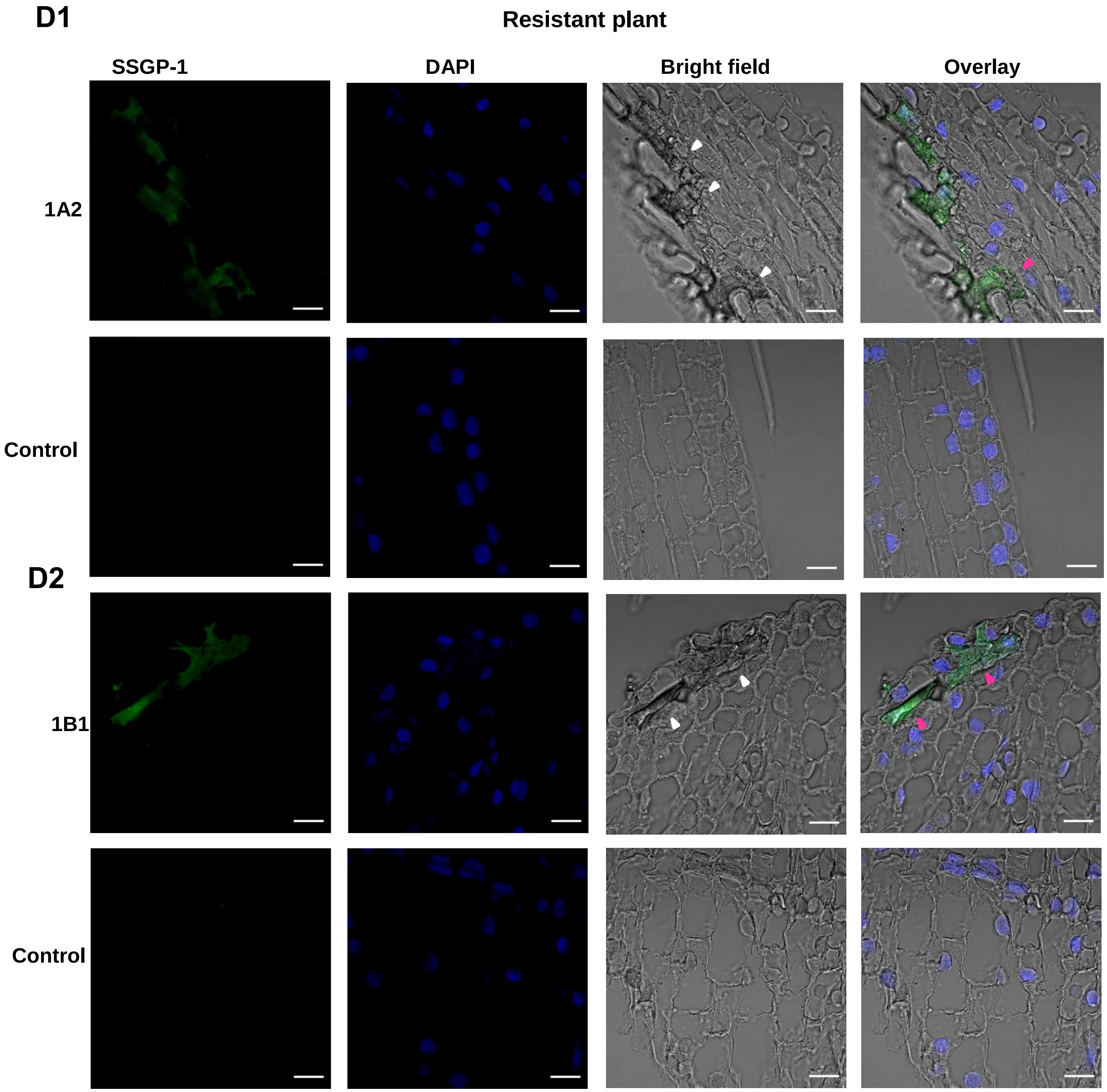

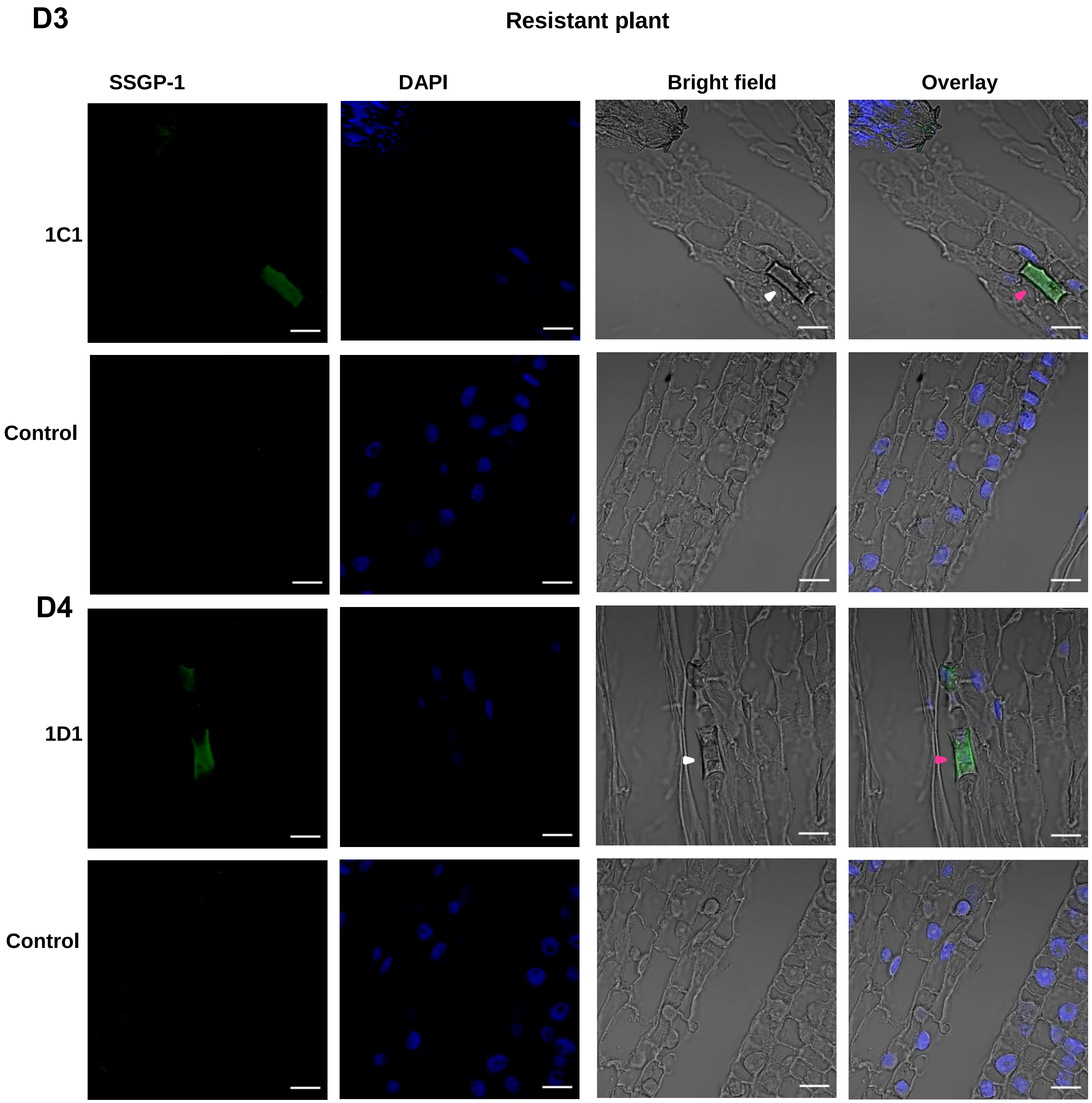
D3
Resistant plant
SSGP-1
DAPI
Bright field
Overlay
1C1
Control
D4
1D1
Control

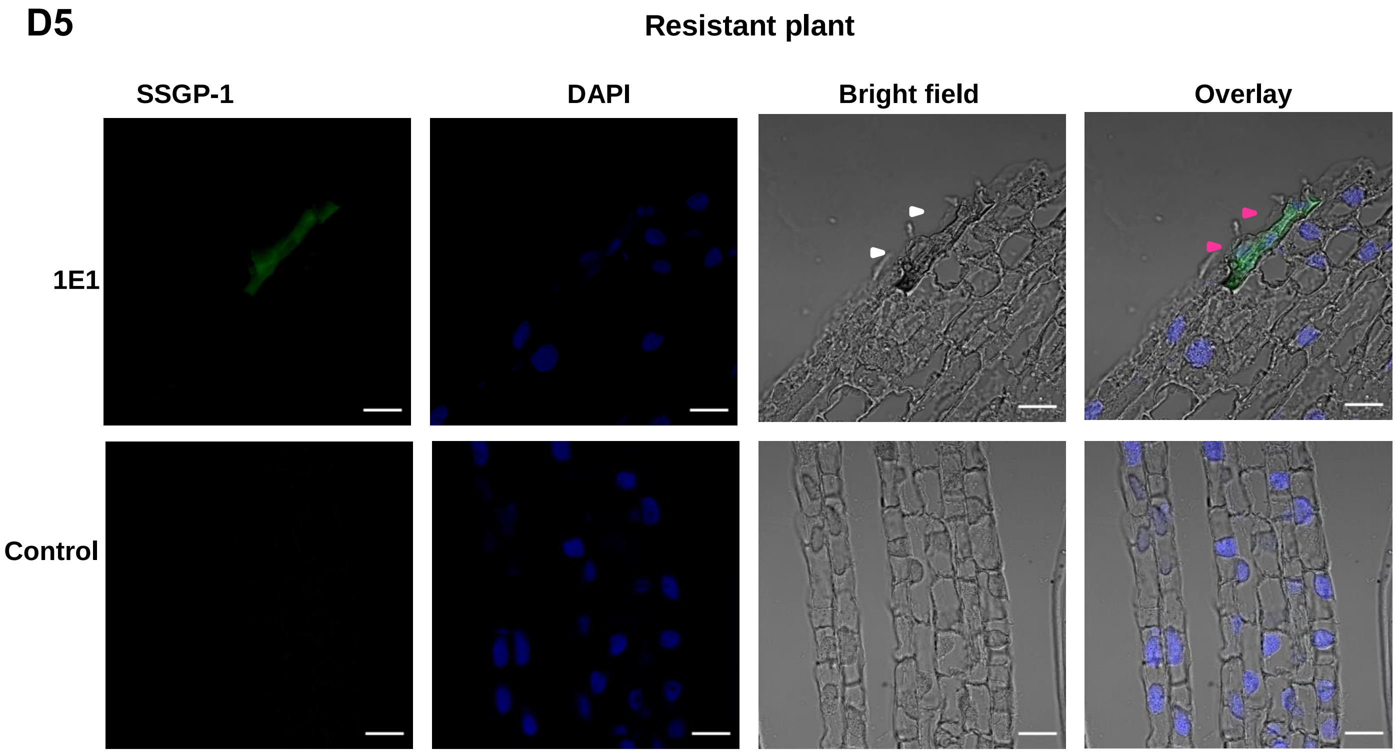
D5
Resistant plant
SSGP-1
DAPI
Bright field
Overlay
1E1
Control

S4,5: Localization of Family-1 effectors in tissues from the resistant cultivar 'H26'. E1 to E5 indicate staining with each of the five antibodies with their corresponding controls.

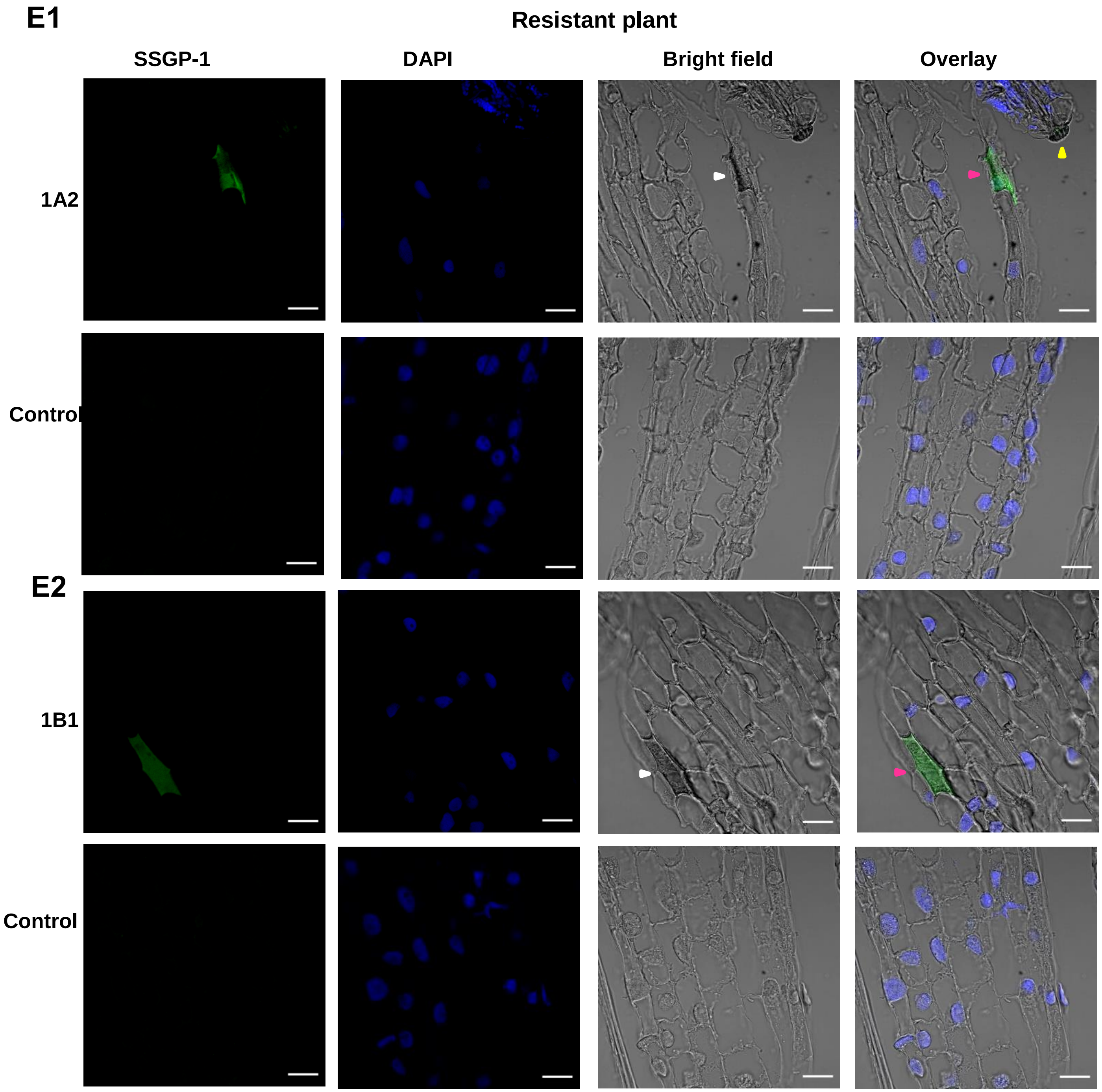

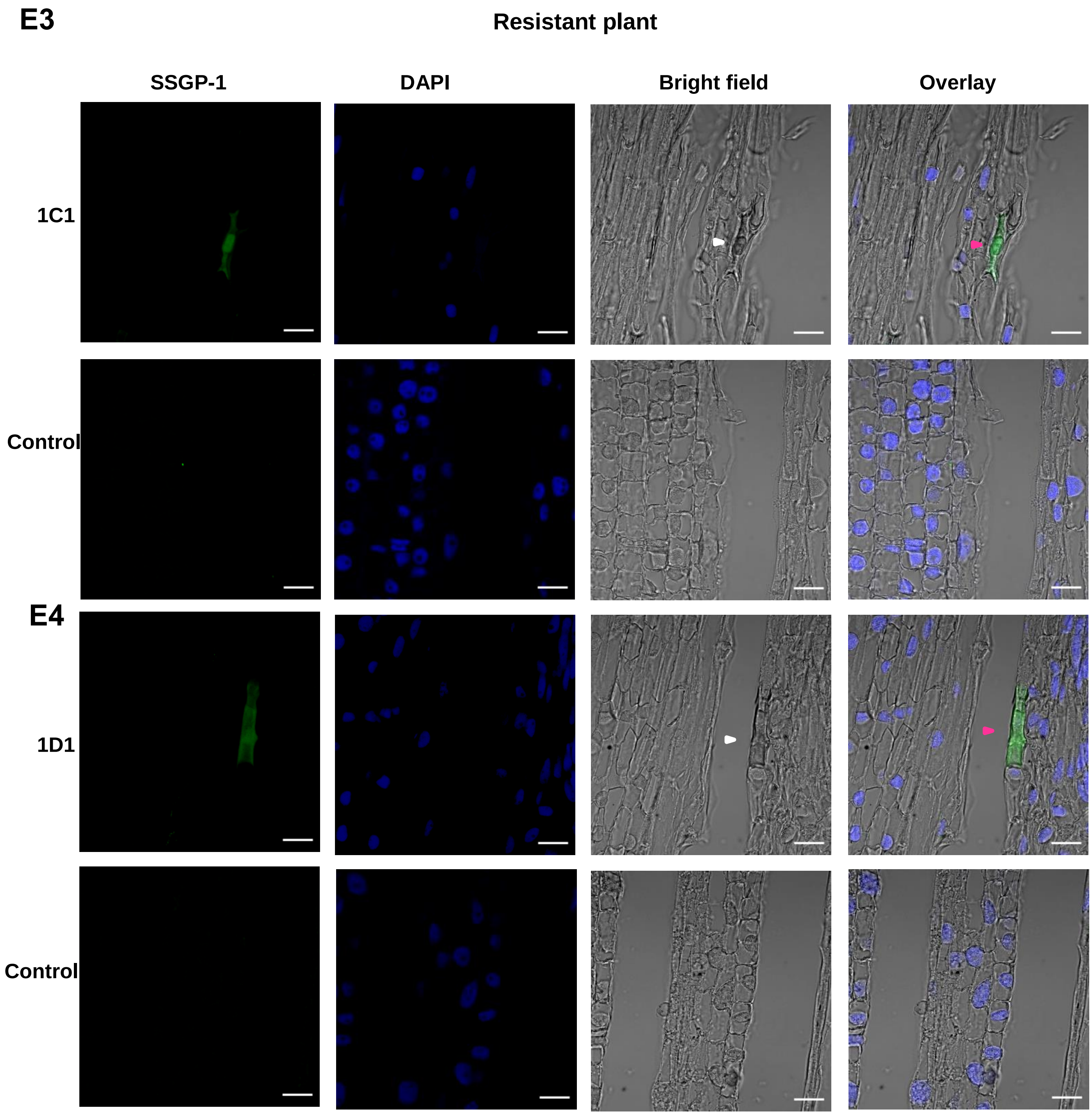
E3
Resistant plant
SSGP-1
DAPI
Bright field
Overlay
1C1
Control
E4
1D1
Control

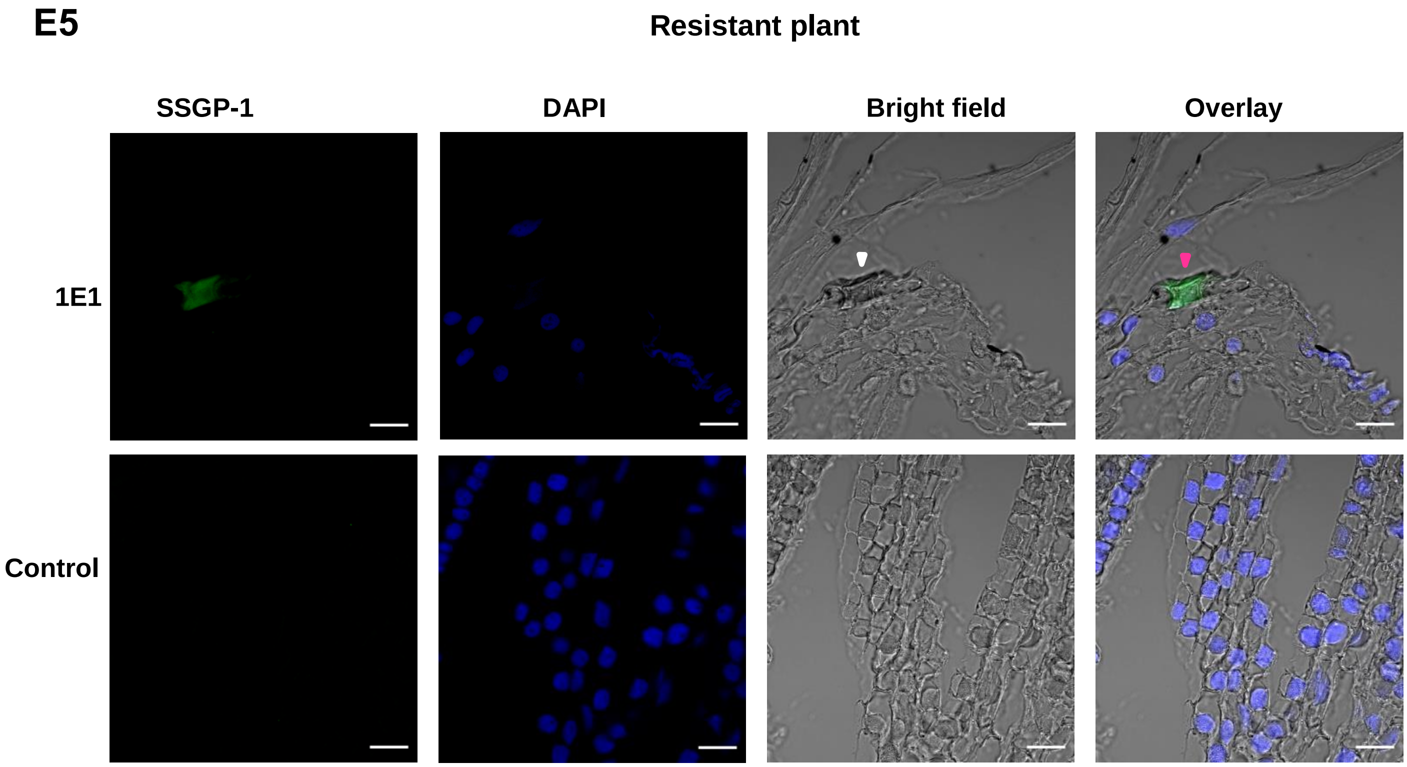
E5
Resistant plant
SSGP-1
DAPI
Bright field
Overlay
1E1
Control

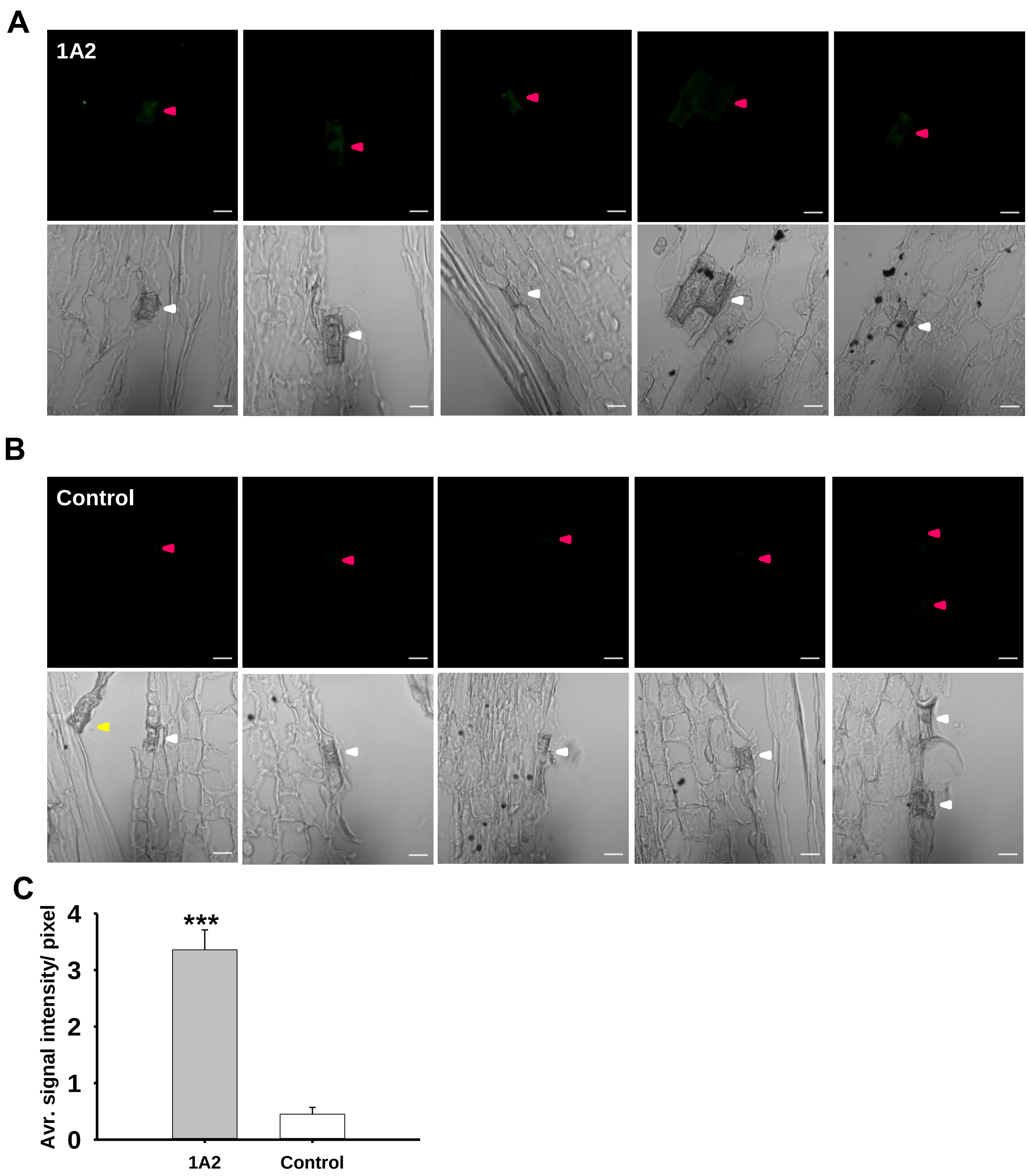

Figure S5. Signal intensity quantification of confocal images after quenching with Vector® TrueVIEW® Autofluorescence Quenching Kit. Paraffin-embedded sections were prepared as indicated in the Methods and stained with antibody against SSGP-1A2 **(A)** and pre-absorbed antibody with its respective antigen as a negative control **(B)**. Autofluorescence signal was observed in the damaged and dead cells in the resistant wheat sections infested with Hessian fly larvae. No autofluorescence background was found in samples from infested susceptible plants. Magenta arrowheads point to the signal of the respective effector. White arrowheads point to dead cells. A yellow arrowhead points to the location of the Hessian fly larva. Bar, 20µm. **C.** Signal intensity (in pixel) was quantified for 1A2 and the pre-absorbed control from representative images for each treatment. The bar graph represents means ± SE of five replicates. The asterisk indicates significant differences between the tested groups, P-value = 2.72033E-09 (P < 0.05, Student's t-test).

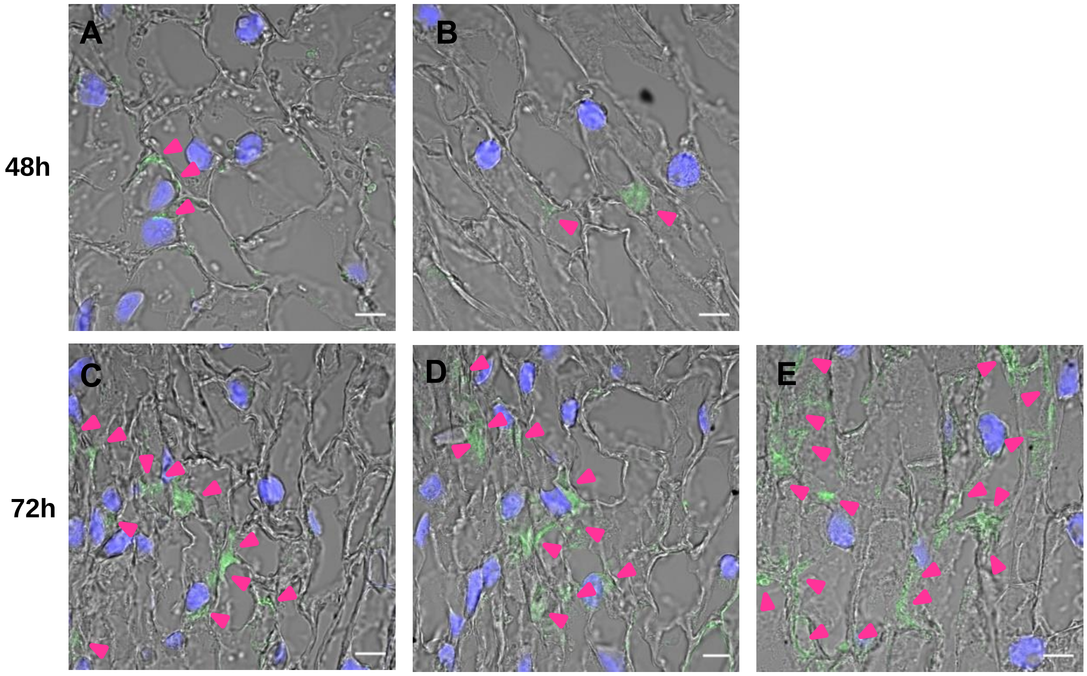

Figure S6. Indirect immunostaining of paraffin-embedded sections from susceptible plant tissues at the attack site with an antibody against the SSGP-1A2 effector was tested in a time course. No signal had been detected in sections collected from samples at 6h to 24h after the initial Hessian fly larval attack. Graphs **A** to **B** were samples collected 48h after the initial larval attack. Graphs **C** to **E** were samples collected 72 h after the initial attack. Magenta arrowheads point to the effector signal in cells at the attacked site. The confocal images show no changes in signal patterns from susceptible plants at time points after three days. Bars, 5µm. Immunostaining assays were independently repeated three times.

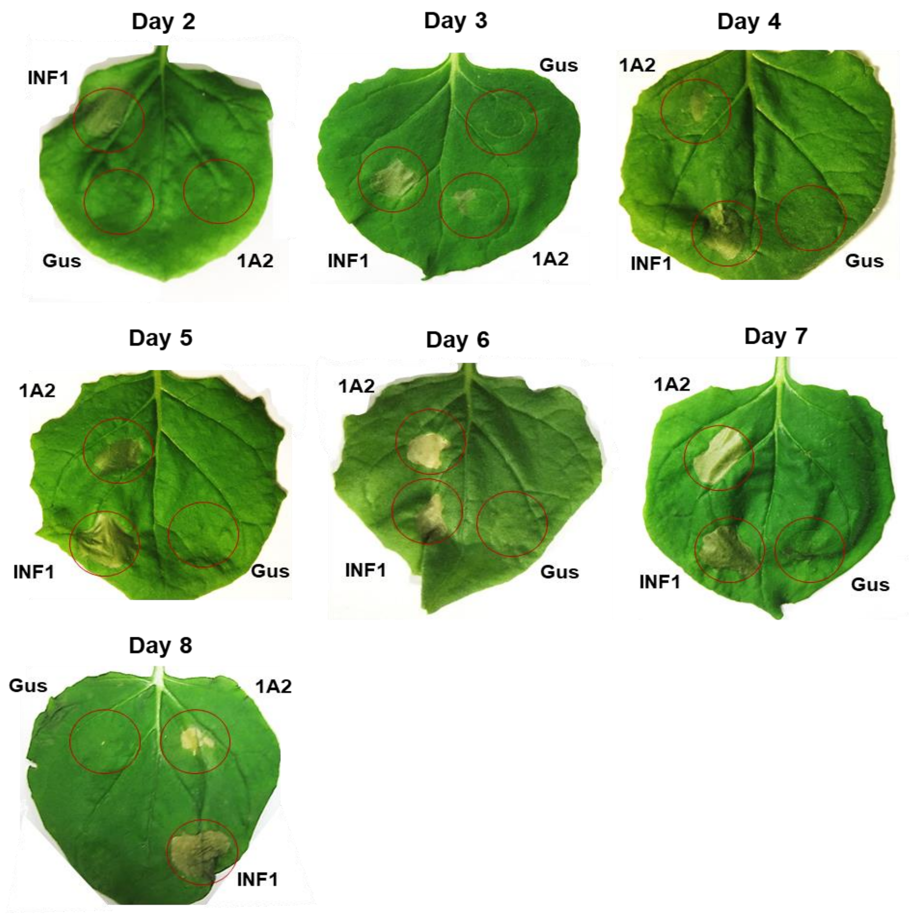

Figure S7. Development of cell death symptoms during the infiltration of *Nicotiana benthamiana* leaves with *Agrobacterium tumefaciens* carrying Gus, INF1, and 1A2. 1A2 is Hessian fly candidate effector SSGP-1A2. INF1 is a positive control that induces HR in *N. benthamiana* leaves. Gus, B-glucuronidase, is a negative control did not induce HR in *N. benthamiana* leaves. Photos were captured on different days after bacteria inoculation. INF1, the positive control, induced symptoms of chlorosis 2 days after infiltration. The chlorosis spots turned into a darker brown color after 5 days and developed severe cell death symptoms after 8 days. Symptoms of chlorosis started to appear after 3 to 4 days after infiltration with Hessian fly effector SSGP-1A2. Cell death symptoms tend to appear as white necrotic lesions after 6-8 days. The infiltration assay was independently repeated three times, with 4 plant replicates.

Table S1. Primer sets used in this study. Small letters in yellow indicate added sequences for restriction enzyme recognition.

A. Primers for expression constructs

SSGP-1A2 F	catatgGCTGTAACTAAACATCCAGCAGG
SSGP-1A2 R	ggatccGGAAGAATGAAAATGGGGGAGATT
SSGP-1B1 F	catatgGCTAAACCTAAAAAAGGCAAAAAGCCC
SSGP-1B1 R	ggatccATGGATAAGAACGGGGGAGATTGTTCT
SSGP-1C1 F	catatgGTACACAGCAGCCATTCCA
SSGP-1C1 R	ggatccCTAAAGAGATTACCTATGGATGAATAAG
SSGP-1D1 F	gctagcGGAGAAACAGATCATTCAGCA
SSGP-1D1 R	ggatccGTTACACGACGTCTCCAAAT
SSGP-1E1 F	catatgGTACAGGAACCACAAGCATC
SSGP-1E1 R	ggatccCAATGGTGGAGATCGTTCTG
ag1A2-Sp F	TACTTTCAATATCCACTGGAACATCC
ag1A2-Sp R	CCTATGGAAGAATGAAAATGGGG
ag1A2 F	ATGGCTGTAACTAAACATCCAGCA
ag1A2 R	GGAAGAATGAAAATGGGGGAGATT

B. Gene-specific primers for qRT-PCR analyses

Accession#	Function based on BLAST analysis	Primer Sequence
AY561153	NADPH-dependent oxidase	F-5' TTGGTGACTGGACACGAGAG
		R-5' AATCCTGAGCAGGAGAACCA
AB078882	Alternative oxidase	F-5' TTCGCATCGGACGTGTACTA
		R-5' CATTTCCTCTGCCTTCCAAA
BE443711	Glycolate oxidases	F-5' TGGAAGGTACATGCACAGGA
		R-5' CTTGAACAACCGAACCCACT
M21962	Oxalate oxidases	F-5' CCTGTTCGCAATGCTGTTAC
		R-5' GCCAACTTGGACGAGAAGAG
BG905395	Amine oxidases	F-5' CTTGATCCAAAGCAACACGA
		R-5' AGATTTGCCGCACCTAGAGA
BQ161967	Class III peroxidase	F-5' CTCCGCTTGATCTGATGGTT
		R-5' CAACGACGATCGAAACCTCT
CK157328	Class III peroxidase	F-5' CGTTTTGTGGGGCTACTTTG
		R-5' CCCATCTCACGAGTGACAGA
CK198851	Class III peroxidase	F-5' CGCTGCGCGTTTGTCA
		R-5' TTGCGTATACCCACACGTAGGT
BQ170589	Class III peroxidase	F-5' GGCTTTCTTCAGGAGCTTTGC
		R-5' CGTGAGCGGCTTGATATTCC
CD373657	Class III peroxidase	F-5' GGTTCTTCGGCCAGTTTGC
		R-5' TTGTGGCCCCTGGAGATTC
EF514209	TaHIR1	F-5' ACAGGCTTAGCAACACCAGG
		R-5' TCGTACCCGTAGGCAGACA
EU908213	TaHIR3	F-5' CTTGAGCCAGGATGCCACT
		R-5' TGCGTCACTTTCTTTGCCA
AK330916	hsr203	F-5' ACCAAGGACCACCCATACAC
		R-5' GTTGATGAGCACCTCCACCT
BJ286329	Epicuticular wax synthesis CER1	F-5' ACCCGCTCTCAGATATGACC
		R-5' GCTCAACTTCGCTACCTTCG
CD910722	Xylanase inhibitor	F-5' AGTCACTGGGCAACAACCTC
		R-5' GAGCACGAAGTCCTCCATCT
CA665158	Xyloglucan fucosyltransferase	F-5' CGCCTGAGCTCCTGTACTTC
		R-5' GAGATGTCCCTCCAAACAGG
CK214572	Xyloglucan endotransglycosylase	F-5' CATGTCGTCCACCATGAAGA
		R-5' AACCCGTACACCCTGAACAC
BJ277428	Glucanase	F-5' AAGGTGTTGCTTCCATGCTT
		R-5' GTTGATGCCCTTGGATTTGT
CJ848656	60S ribosomal protein L21 (RPL21)	F-5' CCAGGTGCAACGAGGAGTTC

		R-5’TCCGCCTTCAGCTTGTCATT
JN247448	NbPR1	F-5’TGAGATGTGGGTCGATGAGA
		R-5’CGAGTTACGCCAAACCACTT
DQ206348	NbPR2	F-5’CAATGCATTAGCAGCAGCAG
		R-5’ATCTTTGGGCGGGTAGGTAT
NM001324627	NbPR3	F-5’TGGGGTTATTGCTGGCTTAG
		R-5’GGGTCATCCAAAACCAGAGA
XM016625792	NbPR4	F-5’GGCCAAGATTCCTGTGGTAGAT
		R-5’CACTGTTGTTTGAGTTCCTGTTCCT
